# Pixelated electrically driven Sb_2_Se_3_ phase-change metasurfaces

**DOI:** 10.1038/s41467-026-73199-6

**Published:** 2026-05-21

**Authors:** Siqing Zeng, Yuru Li, Luoyao Chu, Ruifeng Zhong, Annan Zhao, Yan Li, Shunyu Yao, Xiaojie Zeng, Xiaoqi He, Tao Zhang, Zhaohuan Ao, Zhihao Fu, Zhaohui Li, Chao Lu, Din Ping Tsai

**Affiliations:** 1 Guangdong Provincial Key Laboratory of Optoelectronic Information Processing Chips and Systems, School of Electrical and Information Technology, Sun Yat-sen University, Guangzhou, China; 2 School of Microelectronics Science and Technology, Sun Yat-sen University, Zhuhai, China; 3 Southern Marine Science and Engineering Guangdong Laboratory (Zhuhai), Zhuhai, China; 4 IV-VI PIC Technology Co., Ltd, Hangzhou, China; 5 Photonics Research Institute, Department of Electronic and Information Engineering, The Hong Kong Polytechnic University, Hong Kong, China; 6 Department of Electrical Engineering, City University of Hong Kong, Hong Kong, China; 7 Present Address: School of Optoelectronic Science and Engineering, South China Normal University, Guangzhou, China; 8 Present Address: State Key Laboratory of Photonics and Communications, Department of Electronic Engineering, Shanghai Jiao Tong University, Shanghai, China; 9 Present Address: Institute of Semiconductors, Guangdong Academy of Sciences, Guangzhou, China

**Keywords:** Optoelectronic devices and components, Metamaterials

## Abstract

Sb_2_Se_3_ has established itself as a leading phase-change material for reconfigurable photonics, exhibiting broadband transparency and reversible optical transitions. However, previous metasurface implementations remain constrained by offline thermal or optical control mechanisms. We develop an electrically driven platform featuring monolithic integration of Sb_2_Se_3_ nanostructures with addressable microheater arrays, achieving localized phase transitions at microsecond timescales. This hybrid architecture enables selective excitation of distinct coupled resonant modes in the near-infrared spectrum, delivering electrically controlled amplitude modulation exceeding 80 percent and phase modulation approaching 2π coverage. Advancing beyond unit-cell demonstrations, we implement a 6 × 6 electrically addressable metasurface array that yields an efficient spectral transmission matrix. Here, we show that integrating this system with neural-network-assisted computational methodologies achieves high-precision spectral reconstruction across a 500-nanometer short-wave infrared bandwidth, establishing a robust framework for computational spectroscopy and intelligent sensing in reconfigurable photonics.

## Abstract (web summary)

Phase-change materials cannot be easily integrated into electrically driven metasurfaces. Here, the authors develop a monolithically integrated Sb2Se3 phase-change metasurface composed of a 6 × 6 pixel array, enabling high-speed and broadband control over the phase and amplitude of light.

## Introduction

The precise and dynamic manipulation of spatial light fields underpins advancements across diverse fields, including optical communications, computational imaging, quantum information processing, and augmented/virtual reality. Driven by the growing demand for compact, lightweight, and high-performance integrated optical systems, active spatial light field modulation technologies, particularly those based on metasurfaces, have garnered substantial research interest. Dynamic spatial light field modulation using active metasurfaces has emerged as a focal point in optical and photonic research. While extensive studies have explored various active materials like liquid crystals (LCs)^1–5^, graphene^6–9^, metal oxides^10–13^, and phase-change materials (PCMs)^14–19^, each faces critical limitations for high-performance integrated modulation. LCs, despite mature electrical control and broadband transparency, suffer from low refractive index contrast (~0.1), restricting modulation depth, and relatively slow response speeds (kHz)^20–22^. Materials relying on carrier modulation, such as graphene and metal oxides, can achieve faster speeds (MHz range) but are constrained by limited modulation range and often exhibit considerable intrinsic optical absorption that compromises efficiency when strong light-matter interaction is pursued^23–33^.

Chalcogenide PCMs like Ge_2_Sb_2_Te_5_ (GST) have gained substantial attention for all-solid-state active metasurfaces due to their ultra-high optical contrast and rapid switching speeds^34–39^. This has spurred the development of electrically controlled phase-change metasurfaces, essential for integrated devices. Initial approaches utilized direct electrical control via Joule heating, optimized for speed and efficiency in conductive PCMs like GST^40^. However, this method faces limitations: it requires intrinsic material conductivity and suffers from spatial non-uniformity during crystallization, restricting pixel sizes to the sub-100 nm scale, which is insufficient for optical metasurfaces. To overcome this, indirect electrical control using integrated micro-heaters was developed. This approach has been successfully applied to various PCM, including GST^41,42^ and GSST^43,44^, and validated for both transmissive^43^ and reflective spatial light modulation^41,42,44^. This indirect electrical control approach has been further extended to two-dimensional, addressable phase-change metasurface arrays based on GSST, providing a promising route for pixelated spatial light modulation^45^. Despite these advances in electrical control methodologies, a critical gap remains. Low-loss PCMs, such as Sb_2_S_3_ and Sb_2_Se_3_ have attracted considerable attention due to their uniquely low optical loss in both amorphous and crystalline states, along with the inherent advantage of their binary composition, which suppresses phase separation. Although early studies indicated that their crystallization could suffer from spatial randomness, posing a challenge for large-area, uniform modulation, recent work on spatially confined, nucleation-guided crystallization has demonstrated more uniform phase switching in Sb_2_S_3_, renewing interest in these materials^46^. Nevertheless, their integration into electrically driven phase-change metasurfaces has thus far been relatively unexplored, and a practical, scalable platform for dynamically controlling Sb_2_S_3_ or Sb_2_Se_3_ metasurfaces via integrated electronics has not yet been demonstrated. Although Sb_2_Se_3_ phase switching via PIN heaters in integrated photonics and in metasurfaces as a tunable dielectric environment has been reported^47,48^, these approaches introduce high-resistance microheaters, micrometer-scale pixel constraints, and necessitate complex optical designs to accommodate the high-index Si substrate, severely limiting Sb_2_Se_3_/Sb_2_S_3_ metasurface scope and performance.

In this work, we demonstrate a pixelated electrically-driven Sb_2_Se_3_ phase-change metasurface array using integrated metal microthermal electrodes. This platform achieves versatile spatial light modulation in the near-infrared band. Our design features a hybrid reflective metasurface structure. The integrated metal microthermal electrodes provide excellent electrical and thermal conductivity, enabling rapid, localized Sb_2_Se_3_ phase transitions. Crucially, the bottom metal layer acts as both an efficient heat spreader and an electromagnetic shield, decoupling the thermal/electrical driving layer from the optical functional layer. This decoupling is key, allowing independent optimization of electrode design and metasurface resonances, and enabling true pixel-level independent control across the array. Leveraging the high dielectric constant of Sb_2_Se_3_ and the flexible hybrid design, we excite diverse resonant modes, such as plasmonic and Mie modes, by tailoring unit cell dimensions, meeting broad light field modulation requirements. Experimentally, we realize a 6 × 6 pixel array. Exploiting the material’s high optical contrast and pixel independence, we generate multiple resonances enabling efficient spectral encoding over a wide bandwidth. This facilitates the construction of a high-dimensional, low-correlation spectral transmission matrix with a reduced number of pixels required. Combined with neural network algorithms, we successfully demonstrate integrated broadband spatial spectral reconstruction over a 600 nm effective wavelength range in the short-wave infrared band, showcasing the substantial potential of electrically controlled Sb_2_Se_3_ metasurface arrays for advanced integrated photonics.

## Results

### Electrically driven control of Sb_2_Se_3_ phase-change pixels

**Fig. 1:**
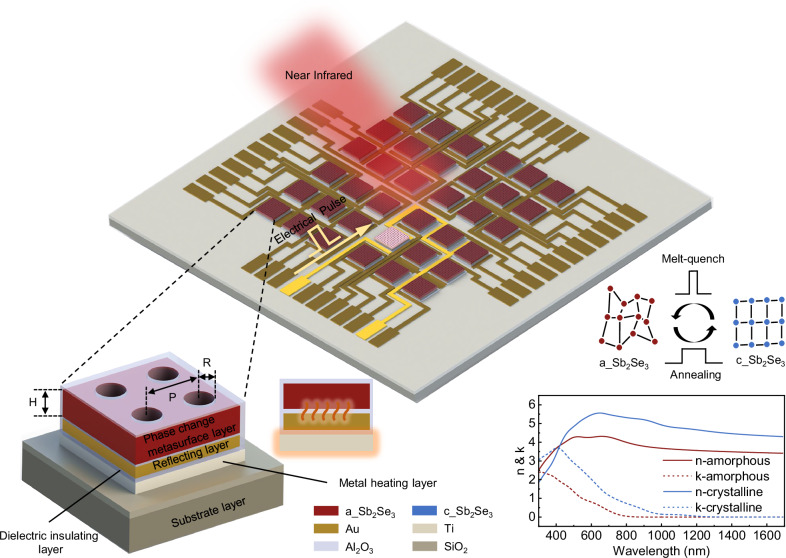
Schematic diagram of the 6×6 Sb_2_Se_3_ phase-change metasurface array. Each metasurface pixel consists of an upper phase-change metasurface layer and a lower metallic microheater electrode layer, with a 10 nm thick cladding layer of aluminum oxide (Al_2_O_3_) inserted in between for electrical insulation. The phase-change metasurface unit is designed as a hybrid metasurface comprising Sb_2_Se_3_ micropores array and a gold (Au) reflective layer. The lower microheater electrode is composed of a titanium (Ti) heating layer and gold electrode leads. Each metasurface pixel in the array is connected to peripheral electrode pads via two gold wires and is electrically connected to the external control circuit through wire bonding. The phase transition of the top metasurface layer, between amorphous and crystalline states, is driven by the heat released from the titanium electrode, which is controlled by externally applied electrical pulses. The inset provides the measured refractive index and extinction coefficient of the Sb_2_Se_3_ films prepared in this work at both amorphous and crystalline states.

The design concept of the two-dimensional electrically driven Sb_2_Se_3_ phase-change metasurface array is shown in Fig. 1. Each metasurface pixel comprises an independent lower electrical control section and an upper phase-change metasurface region. The electrical control of the current phase-change material can generally be divided into direct heating and indirect heating methods. Direct heating operates by passing an electrical current directly through the phase-change material itself via external electrodes, where the resulting Joule heating within the material drives the phase transition. This method is commonly employed for conductive phase-change compounds, such as GST, but is evidently unsuitable for near-insulating materials like Sb_2_Se_3_^40^. Therefore, this work employs the indirect heating method, which involves integrating microheater structures into the lower layer of the metasurface array to drive the phase transition.

Considering the electrical control capability of the pixel and the diverse optical field modulation requirements of the phase-change metasurfaces, this work selects the mature metal microheater combined with a hybrid phase-change metasurface design. The lower microheater part uses the classic thermal electrode material Ti paired with highly conductive Au as the lead, ensuring effective heating in the electrode area while minimizing additional power consumption introduced by the lead. The upper hybrid phase-change metasurface consists of a gold reflective layer, an Al_2_O_3_ dielectric layer, and an Sb_2_Se_3_ micropore array. Instead of utilizing the top gold optical reflector directly as a heating element, in our design, we integrated a dedicated Ti/Au micro-heater beneath it. This architectural choice was driven by two key considerations: thermal stability and the need for spatially localized power dissipation. First, the thermal stability requirement stems from the high transient temperatures needed for the phase-change cycle. Titanium, with its melting point (~1668 °C) much higher than that of gold (~1064 °C), offers superior robustness. This is critical for the re-amorphization (“reset”) process of Sb_2_Se_3_, which demands that the heater briefly exceed 600 °C^49^. The use of Ti thus ensures reliable operation over repeated switching cycles. Second, and more fundamentally, this bilayer design enables efficient spatial confinement of Joule heating. We leverage the pronounced resistivity contrast between Ti and Au: the narrow, micron-scale interconnects are made of low-resistivity Au, while the broader, tens-of-micron heating pad is made of high-resistivity Ti. This configuration ensures that the majority of the heat is generated within the Ti pad directly beneath the phase-change metasurface, where it is needed. A monolithic Au heater would suffer from substantial parasitic heating along the elongated, narrow interconnect traces, drastically reducing the heating efficiency at the target pixel. Supporting finite-element simulations, provided in the Supplementary Note 1 confirm this rationale. They demonstrate that our Ti/Au design effectively confines the temperature rise to the Ti pad region during both crystallization and amorphization pulses, minimizing wasted power and validating the chosen approach. By effectively shielding the electromagnetic field with the gold reflective layer, the optical response of the metasurface is completely decoupled from the microheater structure, ensuring that the electrically-driven Sb_2_Se_3_ metasurface array has the capability to meet diverse optical field modulation requirements. It should be noted that this is also the main reason why this work focuses on the low-loss Sb_2_Se_3_. Previous works on electrically-driven phase-change metasurfaces based on GST and GSST materials have been widely verified^49^. However, limited by the intrinsic absorption characteristics of GST materials in the optical band and the introduction of electrode structures, these works have introduced considerable intrinsic absorption during the design stage, restricting the application scope of the metasurfaces from the very beginning. The current design can effectively avoid the potential impact of the electrode structure on the optical response of the metasurface, fully leveraging the potential of the Sb_2_Se_3_ hybrid metasurface. By exciting various types of resonance modes, such as plasmonic and dielectric resonance modes, the Sb_2_Se_3_ metasurfaces can achieve a wide range of optical field modulation functions, including but not limited to amplitude-dominant and phase-dominant modulation.

Based on a single electrically-driven metasurface pixel, this work further explores the possibility of two-dimensional array extension and demonstrates a 6 × 6 electrically driven Sb_2_Se_3_ metasurface array with independently controllable pixels. Pixelated integrated optical field modulation technology in the optical band has always been a key pursuit in the field of active metasurfaces. Early works on LCs, metal oxides, and other active materials have demonstrated the feasibility of arrayed active metasurfaces. These active materials rely on voltage-driven mechanisms. Consequently, in the arraying process, the packaging scheme of vertically stacked row and column electrodes, commonly used in LC spatial light modulators, can be readily adapted^50^. In previous works, similar arraying schemes have also been used for pixelated electrical control of vanadium oxide active structures^51^. However, the row-column addressing scheme for driving non-volatile PCMs, such as Sb_2_Se_3_, Sb_2_S_3_, and GST remains largely unexplored. In this work, inspired by previously reported vertically stacked row–column electrode architectures, we numerically validate the feasibility of the proposed scheme for selectively driving Sb_2_Se_3_ metasurfaces within a small-scale array through electro-thermal simulations (Please refer to Supplementary Note 2 for detailed simulation results). Combined with its inherent compatibility with high fill factors and straightforward array scalability, the row-column addressing approach presents a promising route toward realizing electrically controlled phase-change metasurface arrays. Nevertheless, considering the fabrication complexity associated with vertically integrated row–column electrode stacks, the present work ultimately adopts a more readily fabricable alternative based on planar-wired micro-heaters. This work adjusts the layout of the planar array electrodes to provide each pixel with an independent control electrode, thereby demonstrating a 6 × 6 electrically-driven phase-change metasurface array with random access capability. The leads of each pixel are drawn out from the pixel gaps and connected to the peripheral electrodes through wire bonding. Clearly, this leads to a layout that severely limits the effective fill factor of the metasurface array. To address this, this work effectively reduces the overall number of required leads by sharing the ground line among pixels and optimizes the lead layout, demonstrating a two-dimensional random-access phase-change metasurface array with a pixel effective size of 75 µm and a pixel pitch of 150 µm, achieving an effective fill factor of nearly 50%.

We emphasize that the choice of a 6×6 array represents a deliberate practical balance for this proof-of-concept study. This scale provides a transmission matrix with sufficient dimensionality to validate the principle of broadband spatial-spectral reconstruction, while remaining manageable for the fabrication and characterization processes. It does not reflect a fundamental physical or architectural limit of the phase-change material or metasurface paradigm itself. The primary limitation on scalability in the current implementation stems from the planar wiring scheme used for individual pixel addressing. As noted, this design entails an inherent compromise between array size and fill factor: scaling the pixel count necessitates more interconnecting leads, which in turn reduces the usable active area. In contrast, the row-column addressed electrode architecture discussed in the Supplementary Note 2 is designed to be compatible with both a high fill factor and straightforward array scaling. By vertically stacking the addressing lines and sharing them across rows and columns, this approach presents a promising and practical pathway for future research toward large-scale, densely integrated phase-change metasurface arrays.

**Fig. 2:**
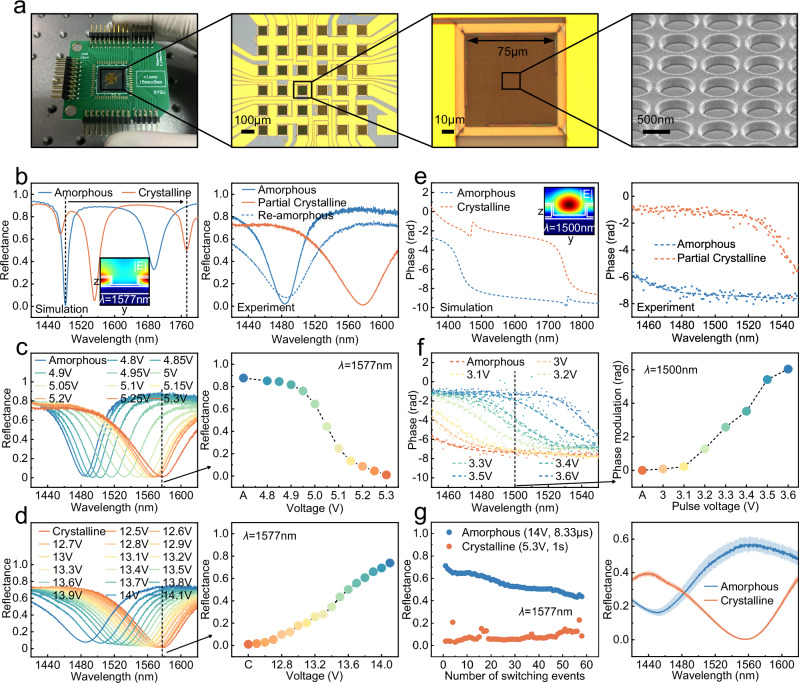
Single-pixel dynamic optical modulation enabled by electrically-driven phase transition of Sb_2_Se_3_ metasurfaces. **a** Microscope image of the fabricated electrically-driven Sb_2_Se_3_ phase-change metasurface array and scanning electron microscope (SEM) image of the micropore array within the metasurface. **b** Simulated (amorphous and fully crystalline) and experimentally measured (amorphous and electrically switched, partially crystallized) reflectance spectra of the under-coupled resonant metasurface designed based on the Sb_2_Se_3_ micropore array. The inset shows the simulated electric field distribution in the amorphous micropore structure supporting the under-coupled resonance mode. **c** Experimentally measured reflectance spectrum of the Sb_2_Se_3_ metasurface under crystallization pulses with different voltages, demonstrating the multi-stage amplitude modulation effect at 1577 nm due to multi-stage crystallization. **d** Experimentally measured reflectance spectrum of the Sb_2_Se_3_ metasurface under amorphization pulses with different voltages, demonstrating the multi-stage amplitude modulation effect at 1577 nm based on multi-stage amorphization. **e** Simulated (amorphous and fully crystalline) and experimentally measured (amorphous and electrically switched, partially crystallized) reflective phase spectra of the over-coupled resonant metasurface designed based on the Sb_2_Se_3_ micropore array. The inset shows the simulated electric field distribution in the amorphous micropore structure when the over-coupled resonance mode is excited. **f** Experimentally measured reflective phase spectrum of the Sb_2_Se_3_ micropore array under crystallization pulses with different voltages, with the reflective phase modulation at 1500 nm during the multi-stage crystallization process highlighted. **g** Experimentally measured changes in the reflectance during approximately 60 phase-transition cycles, with the varying spectrum during 10 selected phase-change cycles shown in detail. Crystallization was driven by a 5.3 V, 1 s pulse, and amorphization was driven by a 14 V, 8.33 µs pulse.

The fabricated electrically driven Sb_2_Se_3_ phase-change metasurface array is shown in Fig. 2a. For detailed fabrication processes, please refer to Supplementary Note 3. Notably, in this work, we chose to construct a periodically arranged negative structure array, the micropores, rather than the commonly used positive structure array, to demonstrate the dynamically tunable optical response. During the Sb_2_Se_3_ metasurface design, we selectively engineered resonant modes into either the under-coupled or over-coupled regime. Under-coupled modes occur when the radiation coupling rate is much smaller than the internal dissipation rate. These modes exhibit strong localized absorption, making them highly effective for achieving high-contrast amplitude modulation. In contrast, over-coupled modes arise when the radiation coupling rate substantially exceeds the internal dissipation rate. They deliver near-complete 2π phase coverage with minimal absorption loss, enabling efficient phase modulation^52,53^. By harnessing both regimes within our hybrid platform, we demonstrate powerful dynamic spatial light field modulation capabilities, encompassing both amplitude-dominant and phase-dominant control, thereby showcasing the versatile application potential of the electrically controlled Sb_2_Se_3_ phase-change metasurface.

Firstly, taking the short-wave infrared band as an example, this work experimentally demonstrated the electrically driven amplitude modulation based on the under-coupled resonant metasurface, as shown in Fig. 2b. By strongly confining the resonance mode within the PCM region between the micropores, the intrinsic absorption of the surrounding metallic reflective layer in the short-wave infrared band results in a pronounced resonant absorption peak at the center wavelength. As the material undergoes a phase transition, the resonance mode experiences a pronounced frequency shift, thereby achieving tunable amplitude modulation at the target wavelength. For detailed design of the phase-change meta-atom, please refer to Supplementary Note 4. In designing our metasurface, we selected a continuous, hole-based (“negative”) pattern over an isolated pillar (“positive”) architecture. This choice was motivated by considerations of phase-change kinetics at the nanoscale. Previous studies on similar systems suggest that the probability of crystallization under a given thermal stimulus can depend on feature size, with smaller, isolated volumes presenting fewer nucleation sites^46^. A continuous network, by comparison, offers a larger effective volume for nucleation per optical unit. While this does not alter the fundamental crystallization temperature of Sb_2_Se_3_, it may reduce the practical thermal energy required to reliably initiate and complete the transition within the metasurface lattice. This design aspect, combined with the low resistance of our integrated micro-heater, contributes to the relatively low driving voltages observed in our platform. A single 5.3 V pulse lasting 1 s can achieve approximately 40% crystallization (please refer to Supplementary Note 4 for crystallization level estimation); whereas a single 14.1 V pulse lasting 8.33 μs can achieve nearly complete amorphization, restoring the reflectance spectrum to a state close to the initial amorphous state. A noticeable decrease in off-resonance reflectivity can be observed in the re-amorphized state compared with the initial amorphous state in the experimental spectra of Fig. 2b. In this work, this reduction is primarily attributed to incomplete re-amorphization, in which residual crystalline nuclei within the Sb_2_Se_3_ film enhance random scattering. This scattering effect can be equivalently described as an effective increase in the material’s intrinsic absorption. Such spectral deviation is consistent with our earlier study on Sb_2_Se_3_ metasurfaces, where the discrepancy between simulated and experimental reflection spectra was accurately reproduced by introducing an additional extinction coefficient into the material’s dielectric constant^54^. Accordingly, this work deliberately operated the phase-change material within a weakly crystallized regime. This approach refers to the active selection and maintenance of a low crystallization fraction, approximately 40% state as mentioned above, by applying electrical pulses with reduced amplitude. While the inherent stochasticity of nucleation in Sb_2_Se_3_ cannot be eliminated, restricting the overall crystallized volume helps mitigate the optical impact of spatially non-uniform crystallization within the unit cell. This strategy reduces the sensitivity of the optical response to such randomness at the targeted operating point, while also lowering the energy required per switching event. Leveraging the substantial optical contrast of Sb_2_Se_3_ between its amorphous and partially crystalline states, this approach enabled an amplitude modulation efficiency of nearly 80%.

In addition, Sb_2_Se_3_ is considered to have the advantage of quasi-continuous phase change control and has been widely validated in waveguide-based photonic devices^47^. Here, by controlling the voltage of the crystallization and amorphization pulses, we experimentally demonstrated multi-stage electrically driven phase change modulation in both the crystallization and amorphization processes. As shown in Fig. 2c, d, the center wavelength of the resonance mode gradually changes with the varying voltage of the crystallization or amorphization pulse, manifesting as multi-stage controllable amplitude modulation at the target wavelength (achieving 12-stage amplitude modulation during crystallization and 18-stage amplitude modulation during amorphization). It is worth noting that current results indicate a more easily achieved multi-level amorphization process compared to the crystallization process. This observation, however, does not imply that amorphization is inherently more controllable than crystallization. In fact, crystallization typically proceeds more gradually, and extensive prior work has successfully demonstrated multi-level crystallization in Sb_2_Se_3_ by carefully tuning electrical pulse parameters^47^. Yet, the characteristic nucleation-and-growth mechanism of Sb_2_Se_3_ introduces inherent spatial stochasticity, making precise and repeatable control over intermediate crystalline states challenging^55^. In contrast, the amorphization (“reset”) process, although more abrupt, is governed by a sharper thermal threshold, requiring the material to briefly exceed its melting point. The absence of a stochastic nucleation step in this regime offers a distinct pathway for multi-level control. Previous studies have shown that by progressively increasing the amorphization pulse amplitude, multiple distinct re-amorphization levels can be achieved, sometimes yielding finer gradation than crystallization under similar conditions^49^. Our own experimental data align with this trend, demonstrating multi-level amorphization with a finer apparent gradation than the crystallization levels obtained under our specific driving scheme. Nevertheless, it is important to emphasize that neither our results nor existing literature conclusively prove that one process is fundamentally more controllable than the other. The observed behavior is highly dependent on material composition, device geometry, and driving conditions. The prevailing view in the field maintains that achieving truly precise and deterministic intermediate states will likely require active feedback mechanisms, such as in situ optical or electrical monitoring, to complement and guide both crystallization and amorphization pulses in a coordinated manner^49^.

Leveraging the low absorption characteristics of Sb_2_Se_3_ at near-infrared, our work further experimentally demonstrated active phase modulation based on the strongly over-coupled resonance. As shown in Fig. 2e, by adjusting the geometric size of the Sb_2_Se_3_ micropore array, the electromagnetic field of the resonance mode was artificially confined within the low-dielectric-constant environment inside the micropores, away from the underlying metallic reflective layer of the metasurface. This effectively reduced the intrinsic absorption of the metasurface, achieving the excitation of a strongly over-coupled resonance mode by ensuring that the resonant scattering loss far exceeded the absorption loss^47^. The strongly over-coupled resonance offers a resonant phase covering a 2π range while maintaining high reflectance near the center wavelength, and is thus commonly used for high-efficiency phase modulation. The experimentally measured reflective phase spectra of the Sb_2_Se_3_ metasurface in the initial amorphous state and after crystallization verify the excitation of the over-coupled resonance mode and confirm that the resonance is maintained after crystallization, thereby providing the necessary conditions for active phase modulation, as shown in Fig. 2e. Detailed experimental protocols are provided in Supplementary Note 5. It should be noted that, in the phase modulation process, we also deliberately used a mild crystallization pulse with a voltage of 3.6 V and a duration of 2 s to keep Sb_2_Se_3_ within a weakly crystallized range of about 40%, to mitigate the impact of random lattice distribution on the phase modulation. By increasing the voltage of the crystallization pulse while keeping the pulse width constant, we also presented multi-stage phase modulation with a modulation range covering nearly 6 radians at a wavelength of 1500 nm, as shown in Fig. 2f, verifying the application potential of the proposed Sb_2_Se_3_ hybrid metasurface in spatial light field modulation. In addition, we systematically evaluated the cycling endurance of the electrically driven Sb_2_Se_3_ metasurface. Under higher driving voltages (5.3 V, 1 s for crystallization; 14 V, 8.33 µs for amorphization), stable phase switching was achieved for approximately 60 cycles before failure (see Fig. 2g). This failure was primarily mechanical, resulting from thermomechanical stress at the Au-Ti electrode interface, as evidenced by post-cycling microscopy (Supplementary Note 6). Post-failure laser-assisted switching confirmed that the phase-change capability of the metasurface material itself remained intact, indicating that the material was not the fundamental limit. Subsequently, we employed lower driving voltages (4.7 V and 13 V). Under these conditions, the device sustained over 850 switching cycles without electrode damage, although a gradual decay in optical modulation depth was observed (Please refer to Supplementary Note 6). Spectral analysis attributes this decay primarily to reduced amorphization efficiency, which we link to cumulative thermal non-uniformity (localized hot spots) in the micro-heater over repeated cycling. It is important to note that these cycle counts do not represent a fundamental limit of the Sb_2_Se_3_ material. Prior work utilizing integrated photonic platforms has demonstrated stable electrothermal cycling exceeding 10^4^ times for Sb_2_Se_3_^49^. Therefore, we anticipate that the cycling endurance of our electrically controlled metasurface can be substantially enhanced by future engineering optimizations focused on improving the mechanical robustness of the heater interface and the thermal uniformity of the micro-heater design.

**Fig. 3:**
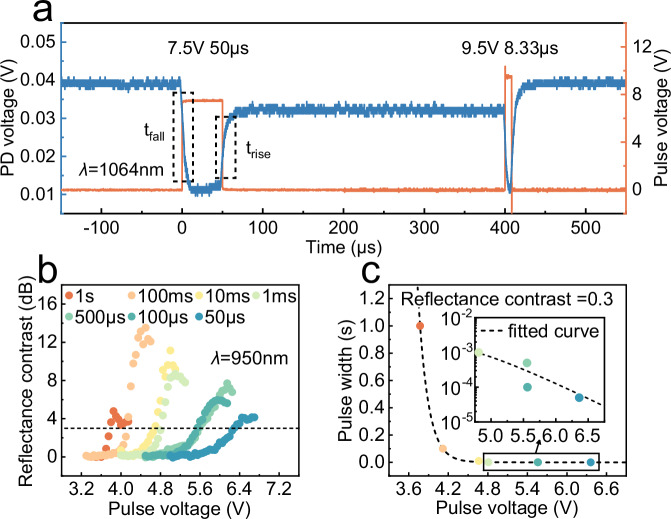
Experimental validation of high-speed electrically-driven phase transition. **a** Time-resolved reflectance evolution during crystallization and amorphization processes. **b** Reflectance contrast at 950 nm as a function of crystallization voltage under different pulse widths. **c** Pulse-width dependence of threshold voltage required for achieving 3-dB reflectance contrast.

In practical implementations, crystallization pulses on the order of seconds inherently impose severe limitations on the response speed of Sb_2_Se_3_ phase-change metasurfaces. Extensive studies have demonstrated that Sb_2_Se_3_ materials exhibit superior high-speed switching characteristics compared to conventional LC systems. Notably, prior research leveraging waveguide-based photonic device platforms has achieved electrically driven Sb_2_Se_3_ phase transitions using ultrashort electrical pulses with a width of 500 ns, validating the feasibility of sub-microsecond switching in integrated optoelectronic systems^56^. Building upon this foundation, this work systematically investigates the high-speed phase-switching performance of the proposed electrically-driven Sb_2_Se_3_ metasurface architecture. While previous works typically utilized the minimum electrical pulse width required for crystallization as the primary metric for device speed assessment, the actual phase transition duration often markedly undercuts the applied pulse width. As illustrated in Fig. 3a, we employ high-speed photodetectors to capture real-time optical response evolution of Sb_2_Se_3_ metasurfaces during electrically-driven crystallization and amorphization processes. The experimental results confirm that 50 μs pulses suffice to induce effective crystallization. Notably, the transient photocurrent profile during crystallization reveals concurrent volatile and non-volatile modulation mechanisms. The volatile component likely originates from the combined effects of intrinsic thermo-optic response and transient structural deformation of the metasurface under thermal stress, while the non-volatile modulation naturally stems from crystallization-induced refractive index changes. Temporal overlap between these two modulation mechanisms complicates individual response time characterization. Nevertheless, the rapid stabilization of the photocurrent observed during the pulse indicates that the crystallization process completes much faster than the 50 µs duration of the applied electrical pulse, consistent with a swift phase-transition kinetics. Post-pulse cooling analysis reveals rapid photocurrent recovery within 10.75 μs, demonstrating efficient thermal dissipation crucial for reversible phase cycling. Subsequent application of a 9.5 V/8.33 μs pulse achieves successful amorphization, with comparatively faster response times despite similar dual modulation characteristics. The crystallization process, typically requiring longer durations than amorphization, remains the primary speed-limiting factor in phase-change photonic devices. Therefore, in this work, we further explore crystallization pulse width requirements through systematic voltage-dependent characterization.

As shown in Fig. 3b, c, our work has characterized the variation of the crystallization level of the Sb_2_Se_3_ metasurface with pulse voltage under different pulse width conditions. The crystallization level is evaluated by the reflectance contrast at 950 nm before and after crystallization (Please refer to Supplementary Note 7 for detailed computational methodology of the reflectance contrast). Apparently, by increasing the pulse voltage to over 5 V, this study has successfully reduced the required pulse width for crystallization to 50 μs. This switching speed is comparable to that of conventional high-speed spatial light modulators, such as LC devices and digital micromirror devices, with its overall speed primarily constrained by the thermal time constant of the integrated micro-heater. However, the inherent phase-change kinetics of Sb_2_Se_3_ holds the potential for much faster modulation. Prior studies using direct optical excitation have demonstrated full crystallization–amorphization cycles within ~12 µs, suggesting a fundamental material response capable of reaching the hundreds of kHz regime^57^. Therefore, while the present device speed is on par with established technologies, future optimization of the driving architecture could unlock substantially higher switching rates, leveraging the intrinsic fast phase-transition capability of Sb_2_Se_3_ for applications demanding non-volatile, high-speed optical modulation. However, it is worth noting that as the pulse width is reduced, the maximum optical contrast achievable during the crystallization process continues to decline. In other words, for the proposed Sb_2_Se_3_ phase-change metasurface, the crystallization response speed and the achievable degree of crystallization are mutually restrictive. It is believed in this study that the main reason for the limited crystallization level during rapid crystallization is the inevitable non-uniform heating of the underlying thermal electrode during the heating process. Especially for metasurface pixels built on large-footprint heaters, crystallization pulses with narrow width and high voltage tend to create pronounced local hot spots, which in turn limit the overall crystallization uniformity across the pixel. This thermal gradient stems directly from the extended area of the Au/Ti micro-heater and is clearly illustrated in the accompanying electro-thermal simulations (Please refer to Supplementary Note 1). We regard such heater-induced temperature non-uniformity as a key factor constraining the controllability of the phase transition in the current geometry. Previous works have shown that optimizing the heater morphology can effectively mitigate this issue, pointing toward a promising direction for further improving the performance of electrically driven phase-change metasurfaces^44^. Furthermore, the crystallization pulse voltage required to achieve a 3 dB reflectance contrast under different pulse width conditions is presented in Fig. 3c. From the trend of pulse voltage variation, it can be intuitively inferred that by further increasing the pulse voltage, the required pulse width for crystallization could be further reduced, potentially approaching the high-speed driving effect at the 500 ns level demonstrated by waveguide-based integrated devices. However, in the actual testing process, when the pulse width is reduced to 25 μs and below, the crystallization process with high-voltage pulses is highly likely to cause damage to the micro-thermal electrode structure or the top metasurface layer. The failure of narrow-pulse crystallization in this study is mainly attributed to the structural stress caused by the inevitable material expansion in the multi-layer metasurface structure during the heating process. Therefore, in practical applications, it is necessary to comprehensively consider the device’s requirements for optical contrast and response speed to select the appropriate phase-change pulse width.

### Spectral reconstruction using pixelated Sb_2_Se_3_ arrays

Based on the proposed Sb_2_Se_3_ metasurface pixels, this work constructs a two-dimensional, electrically driven, phase-change metasurface array, achieving an array size of 6×6. However, as previously discussed, PCMs are power-driven active materials. During the construction of the array, the electrode leads of the central pixels inevitably limit the duty cycle to only 50%. While such an array size and duty cycle are insufficient to meet the requirements of practical light field modulation, the diverse spectral characteristics of the phase-change hybrid metasurface confer unique advantages in the field of computational spatial-spectral reconstruction.

Spatial-spectral reconstruction via metasurface arrays fundamentally relies on constructing a high-dimensional spectral transmission matrix with distinct spectral responses. While conventional approaches require large-scale static arrays to ensure sampling accuracy, this inevitably compromises spatial resolution and impedes applications in spectral imaging^58,59^. To overcome this limitation, we propose and implement a strategy that utilizes the proposed tunable phase-change metasurfaces array. The core concept is to expand the effective dimensionality of the transmission matrix not by increasing the number of physical pixels, but by leveraging the giant optical contrast between the amorphous and crystalline states of a single pixel, thereby preserving a compact form factor and high spatial resolution.

To systematically evaluate the potential of active tuning, we established a comparative framework designed to decouple the contributions of physical array scale from those of spectral reconfigurability. This framework involves constructing two transmission matrices of identical order (36 spectral responses) via fundamentally different strategies, one static and one dynamic, and then comparing their performance in spectral reconstruction. The static transmission matrix is constructed directly from the amorphous-state spectral responses of all 36 pixels in our fabricated 6 × 6 Sb_2_Se_3_ metasurface array. The geometric parameters (period and hole radius) of these units were varied such that their resonance wavelengths differ and collectively span the target near-infrared band. It is important to note that this set of 36 designs was not extensively optimized for ultimate reconstruction performance; its primary purpose is to provide a baseline matrix generated purely through spatial multiplexing of fixed spectral features. The dynamic transmission matrix is built using only 18 physically distinct pixels selected from the same 6 × 6 array. The selection criterion was to ensure comprehensive spectral coverage: we chose pixels whose amorphous-state resonances are located primarily in the short-to-middle wavelength regions. When electrically switched to their crystalline state, these same pixels exhibit substantial resonance redshifts, thereby covering the middle-to-long wavelength regions of the band. Thus, by utilizing both the amorphous and crystalline states of this subset, we generate a complete set of 36 distinct spectral responses. This selection was not exhaustively optimized for parameters, such as mutual correlation but was deliberately designed to validate the core premise: that phase-change-induced spectral shifting can functionally expand the dimensionality of a transmission matrix without increasing the number of physical pixels.

**Fig. 4:**
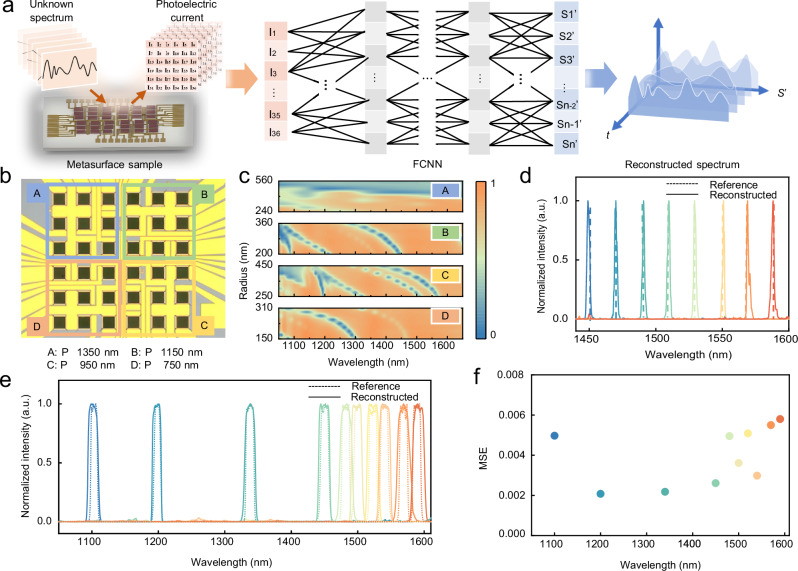
Experimental validation of spatial spectral reconstruction using the 6×6 Sb_2_Se_3_ metasurface array. **a** Schematic illustration of the spatial spectral reconstruction process based on the metasurface array. **b** Microscopic image of the fabricated metasurface array for spatial spectral reconstruction. **c** Experimentally measured spectral transmission matrix constructed from the reflection spectra of the metasurface array. **d** Performance validation of single-wavelength spectral reconstruction within the wavelength range of 1450–1590 nm. **e** Reconstruction of the transmission spectra of narrowband filters within the wavelength range of 1100–1590 nm. **f** Mean squared error (MSE) of the reconstructed transmission spectra of narrowband filters with different central wavelengths.

Our experimental demonstration begins by validating the spectral reconstruction performance using a static metasurface array. The detailed spectral reconstruction process is shown in Fig. 4a. First, the reflection spectra ${T}_{i}\left(\lambda \right){|}_{i=\{1,36\}}$ of each pixel in the metasurface array in the short-wave infrared band is individually calibrated to construct the spectral transmission matrix. Subsequently, the spectral transmission matrix is used to spectrally sample the incident unknown spectrum $S(\lambda )$, resulting in the reflected photocurrent of the metasurface array ${I}_{i}={\int }_{{\lambda }_{1}}^{{\lambda }_{2}}T\left(\lambda \right)\eta \left(\lambda \right)S\left(\lambda \right)d\lambda$, where $\eta \left(\lambda \right)$ represents the quantum efficiency of the photodetector. In the spectral recovery stage, this work establishes a fully connected neural network (FCNN) and completes the training using a randomly generated dataset to establish the mapping relationship between the photocurrent ${I}_{i}$ and the predicted spectrum ${S}^{{\prime} }(\lambda )$ within the target band. Based on this neural network, the incident spectrum is recovered according to the experimentally calibrated photocurrent. For detailed information on the neural network training process and the experimental spectral recovery process, please refer to Supplementary Note 8. The spectral transmission matrix of the current metasurface array is presented in Fig. 4b, c. To construct a weakly correlated transmission matrix with broad spectral coverage, during the metasurface design process, the array pixels are divided into four groups. By changing the radius of the micropore structure within each group and the period of the micropores array between different groups, pronounced resonant features are ensured across the 1050–1600 nm wavelength range, effectively reducing the correlation of the spectral transmission matrix. For a detailed discussion on the cross-correlation of the spectral transmission matrices, please refer to Supplementary Note 9. Based on this transmission matrix, this work demonstrates high-precision single-wavelength detection and broadband spectral recovery in the short-wave infrared band. Here, a swept-narrow-linewidth laser (Keysight, 8164B) is used to generate eight groups of ultra-narrow-linewidth lasers with equally spaced wavelengths covering the range of 1450–1590 nm to evaluate the single-wavelength identification. The experimental results in Fig. 4d clearly indicate high-precision single-wavelength detection, with an average wavelength detection error of only 0.787 nm. Using multiple commercial bandpass filters with center wavelengths distributed in the 1100–1590 nm range, we further evaluate the spectral recovery performance across a broad spectral range. As shown in Fig. 4e, f, the spectral recovery results of all involved bandpass filter transmission spectra are highly consistent with the calibration results from a commercial spectrometer, and the MSE of all filter spectral reconstruction remains below 0.006, fully demonstrating the applicability of the 36-channel spectral transmission matrix constructed by the current metasurface array across a broad spectral range.

**Fig. 5:**
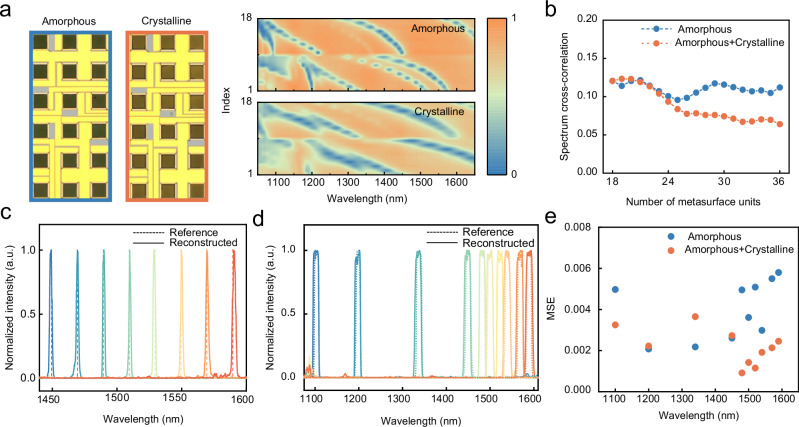
Validation of spatial spectral reconstruction performance using a reduced-scale array considering phase-change spectral modulation. **a** Microscope images of the selected 18 Sb_2_Se_3_ metasurface pixels before and after crystallization, and the 36-channel spectral transmission matrix constructed using the reflectance spectra at amorphous and crystalline states. **b** Evolution of the spectral cross-correlation coefficients of the transmission matrices for static and phase-change metasurface arrays with the increase of the size of the spectral transmission matrix. **c** Single-wavelength detection performance within the 1450-1590 nm wavelength range using the phase-change spectral transmission matrix. **d** Spectral reconstruction of transmission spectra for band-pass filters within the 1100-1590 nm range using the phase-change spectral transmission matrix. **e** Comparison of the MSE for broadband spectral reconstruction between static and phase-change metasurface arrays.

Following the validation of the 36-channel transmission matrix constructed from the static metasurface array, which demonstrates effective spectral reconstruction across the 1100–1590 nm band, we next use its performance as a baseline for comparison. We then evaluate the reconstruction capability of a second 36-channel matrix built from only 18 physical pixels by leveraging both their amorphous and crystalline states. This direct comparison within the identical spectral window allows us to assess the effectiveness of using phase-change tuning to expand the dimensionality and sampling capacity of a compact metasurface array. The reflection spectra of 18 selected metasurface pixels in both the amorphous and crystalline states are used to construct a 36-channel spectral transmission matrix, as shown in Fig. 5a. It can be observed that the crystallization of Sb_2_Se_3_ causes a frequency shift in the resonance modes, and the line shapes of the resonant spectra also change markedly before and after the phase change, thereby enriching the spectral features of the transmission matrix. Here, we introduce and apply a spectral cross-correlation coefficient as a quantitative metric to evaluate and compare the spectral sampling performance of the two 36-channel transmission matrices. In essence, the spectral cross-correlation coefficient evaluates the broadband sampling capability by measuring the average changes in the transmission matrix at different wavelengths within the target spectral range (Detailed calculation process is provided in the Supplementary Note 9). The variation in spectral cross-correlation with increasing matrix size during the construction of the two types of spectral transmission matrices is shown in Fig. 5b. Specifically, we treated the 18 amorphous-state spectra common to both matrices as a shared baseline. Starting from this baseline (size = 18), we then simulated the expansion of the transmission matrix in the two distinct ways and tracked the corresponding evolution of the spectral cross-correlation coefficient. This methodological choice is precisely why the horizontal axis in Fig. 5b begins at 18. The blue curve shows the trend when the matrix is expanded by sequentially incorporating the spectral responses of new static metasurface designs from the remaining pool. The red curve illustrates the trend when the matrix is expanded by instead incorporating the spectral responses obtained from the phase change (crystalline state) of the same 18 selected pixels. The data indicates that both methods can expand the matrix while maintaining a relatively low mutual correlation. Notably, the phase-change-based expansion appears to yield a slightly lower mutual correlation coefficient as the matrix size increases, suggesting a potentially more efficient spectral sampling capability. As illustrated in Fig. 5c, d, e, the current work experimentally validates high-precision single-wavelength identification and broadband spectral reconstruction in the short-wave infrared band using the phase-change spectral transmission matrix. The average error for single-wavelength identification is as low as 0.325 nm, while the MSE for broadband spectral reconstruction is maintained below 0.004. Both metrics surpass the reconstruction accuracy achievable with a static spectral transmission matrix, thereby underscoring the distinct advantages of the phase-change metasurface array in spatial spectral reconstruction.

The central purpose of this comparative experiment is not to prove that expanding the matrix via phase-change modulation necessarily yields superior reconstruction performance compared to incorporating additional, distinct metasurface units. Rather, it robustly demonstrates that utilizing the dynamic spectral modulation of phase-change metasurfaces provides an effective and viable pathway to expand a transmission matrix. This validation underscores the strong potential of tunable metasurface arrays for achieving high-performance spectral reconstruction with a compact, small-scale physical footprint.

## Discussion

In summary, the current work, leveraging the low-loss phase-change material Sb_2_Se_3_, experimentally demonstrates an electrically controlled two-dimensional phase-change metasurface array. To fully exploit the advantages of Sb_2_Se_3_, including its low loss and high optical contrast in the short-wave infrared band, we propose an electrically controlled metasurface pixel design architecture that combines metallic microheater electrodes with hybrid phase-change metasurfaces. This design achieves complete decoupling between the electrically induced phase change and the optical response of the metasurface. By selectively confining the electromagnetic fields of the metasurface resonance modes within the phase-change microstructures or the surrounding dielectric environment, the current work experimentally demonstrates the excitation of both under-coupled and over-coupled resonance modes. In the short-wave infrared band, we achieve amplitude-dominant modulation with an efficiency of 80% and phase-dominant modulation covering nearly 6 radians, thereby verifying the multifunctional characteristics of the electrically controlled metasurface pixels. Moreover, benefiting from the low resistance and high thermal conductivity of the metallic microheater electrodes, the current work showcases low-threshold-voltage and high-speed (crystallization pulse time of 50 μs and amorphization pulse time of 8.33 μs) electrically induced phase change control on 75-µm Sb_2_Se_3_ metasurface pixels. This indicates the considerable potential of the current metasurface pixel design for high-speed spatial light field modulation and other applications. Building on this, the current work further demonstrates a two-dimensional electrically controlled phase-change metasurface array, with an array size of 6×6 and an effective fill factor close to 50%. Using integrated spatial spectral reconstruction as an example, we highlight the multifaceted application value of the two-dimensional electrically controlled phase-change metasurface array. By leveraging the densely packed multi-resonance modes excited by the Sb_2_Se_3_ hybrid phase-change metasurface and combining phase-change spectral modulation, the current work achieves broadband, high-precision spatial spectral reconstruction in the short-wave infrared band with an effective bandwidth coverage of nearly 500 nm (1100–1590 nm) using only 18 metasurface pixels. Overall, our work holds considerable importance for promoting the development of integrated phase-change spatial light field modulation technologies and devices, as well as for expanding the applications of phase-change spatial light field modulation technologies in sensing, imaging, computing, and other fields.

## Methods

### Material synthesis

Sb_2_Se_3_ thin films were fabricated by thermal evaporation from a single Sb_2_Se_3_ source. The precursor material was synthesized by the conventional melt-quench method with high-purity (5 N) constituent elements. Film deposition was conducted in a custom-built high-vacuum thermal evaporation chamber. The as-deposited films were amorphous and were subsequently transformed into the crystalline phase by rapid thermal annealing at 220 °C for 5 min under a nitrogen atmosphere. The optical constants (refractive index n and extinction coefficient k) of both amorphous and crystalline Sb_2_Se_3_ films were determined by spectroscopic ellipsometry (J.A. Woollam Co.), as shown in Fig. 1.

### Device fabrication

The pixelated electrically driven Sb_2_Se_3_ metasurfaces were fabricated through multi-step electron-beam lithography (EBL) and thin-film deposition. Initially, the micro-heaters (Ti/Au/Ti) and Al_2_O_3_ dielectric layers were patterned on a quartz substrate using EBL, followed by electron-beam evaporation. Subsequently, the functional Sb_2_Se_3_ layer was deposited via vacuum thermal evaporation, followed by the deposition of an Al_2_O_3_ capping layer. The metasurface nanostructures (meta-atoms) were then defined by a second EBL step and transferred into the Sb_2_Se_3_ layer using reactive ion etching (RIE). This integration enables precise spatial overlap between the thermal bias and the phase-change pixels. A comprehensive description of the fabrication protocols and specific parameters is provided in Supplementary Note 3.

### Optical simulation

The optical performance of the proposed metasurface was numerically simulated using the finite-difference time-domain (FDTD) method, implemented in the commercial software Lumerical FDTD. The simulation model incorporated a single meta-atom under periodic boundary conditions along the *x* and *y* directions to represent the periodic metasurface structure. Perfectly matched layers (PML) were applied at the top and bottom boundaries to emulate an open space environment. The optical constants of Sb_2_Se_3_ were defined using experimentally measured ellipsometry data, while the material parameters for Au, Ti, Al_2_O_3_, and Si were taken from Lumerical’s built-in database. The intermediate phase of Sb_2_Se_3_ was approximated as a homogeneous medium with a dielectric function interpolated between the amorphous and crystalline states using the Lorentz–Lorenz effective medium approximation. Further details of the simulation setup are provided in Supplementary Note 4.

### Tunable metasurface characterization

In this work, the unit cell size of the Sb_2_Se_3_ metasurface array is 75 × 75 μm. After wire bonding to an external printed circuit board (PCB) using Au wires, the electrical resistance of a single pixel was measured to be approximately 12 Ω. The reflection and phase spectra of the metasurface were characterized using a home-built near-infrared micro-spectroscopy system combined with a coherent phase detection setup developed in the author’s laboratory. Detailed descriptions of the optical system can be found in Supplementary Note 5. During testing, electrical pulse signals - generated by an arbitrary waveform generator (Keysight) and amplified by a power amplifier (Aigtek) - were applied to the wire-bonded metasurface device to trigger phase transition. This setup enabled in-situ measurement of the optical response of the phase-change metasurface under active electrical control.

## Supplementary information

Supplementary Information

Transparent Peer Review file

## Data Availability

The source data generated in this study have been deposited in the Figshare database. Source Data file has been deposited in Figshare under accession code 10.6084/m9.figshare.30093232^60^. The processed data underlying Figs. 1–5 are provided with this paper. All other data supporting the findings of this study are available from the corresponding authors upon request.

## Code availability

The custom code used in this study has been deposited in Code Ocean and are split into two parts for clarity. The code for phase retrieval is available at 10.24433/CO.8670907.v1. The code for spectrum reconstruction is available at 10.24433/CO.0109178.v1.

## References

[1] Kang, D. et al. Liquid crystal-integrated metasurfaces for an active photonic platform. *Opto-Electron. Adv.* **7**, 230216 (2024).

[2] Wu J. et al. Liquid crystal programmable metasurface for terahertz beam steering. *Appl. Phys. Lett.* **116**, 131104 (2020).

[3] Zhuang, X. et al. Active terahertz beam steering based on mechanical deformation of liquid crystal elastomer metasurface. *Light. Sci. Appl.* **12**, 14 (2023). 36596761 10.1038/s41377-022-01046-6 PMC9810742

[4] Lan, F. et al. Real-time programmable metasurface for terahertz multifunctional wave front engineering. *Light. Sci. Appl.* **12**, 191 (2023). 37550383 10.1038/s41377-023-01228-w PMC10406829

[5] Li W. et al. Dual-color terahertz spatial light modulator for single-pixel imaging. *Light Sci. Appl.* **11**, 191 (2022). 10.1038/s41377-022-00879-5 PMC9225988 35739086

[6] Almawgani, A. H. M. et al. A graphene-metasurface-inspired optical sensor for the heavy metals detection for efficient and rapid water treatment. *Photonics* **10**, 56 (2023).

[7] Zhou, R. et al. Label-free terahertz microfluidic biosensor for sensitive DNA detection using graphene-metasurface hybrid structures. *Biosens. Bioelectron.* **188**, 113336 (2021). 34022719 10.1016/j.bios.2021.113336

[8] Yang, D. et al. Efficiency tunable broadband terahertz graphene metasurface for circular polarization anomalous reflection and plane focusing effect. *Diam. Relat. Mater.* **131**, 109605 (2023).

[9] Luo, W. et al. Electrically switchable and tunable infrared light modulator based on functional graphene metasurface. *Nanophotonics* **12**, 1797–1807 (2023). 39634112 10.1515/nanoph-2023-0048 PMC11501403

[10] Chang, W. J. et al. Plasmonic metal oxide nanocrystals as building blocks for infrared metasurfaces. *Acc. Mater. Res.* **6**, 104–113 (2024).

[11] Venkatesh, S. et al. A high-speed programmable and scalable terahertz holographic metasurface based on tiled CMOS chips. *Nat. Electron.* **3**, 785–793 (2020).

[12] Sokhoyan, R. et al. Electrically tunable conducting oxide metasurfaces for high power applications. *Nanophotonics* **12**, 239–253 (2023). 39634855 10.1515/nanoph-2022-0594 PMC11501733

[13] Sun K. et al. Wafer-scale 200 mm metal oxide infrared metasurface with tailored differential emissivity response in the atmospheric windows. *Adv. Opt. Mater.* **10**, 2200452 (2022).

[14] Folland, T. G. et al. Reconfigurable infrared hyperbolic metasurfaces using phase change materials. *Nat. Commun.* **9**, 4371 (2018). 30349033 10.1038/s41467-018-06858-y PMC6197242

[15] Michel A. K. U. et al. The potential of combining thermal scanning probes and phase-change materials for tunable metasurfaces. *Adv. Opti. Mater.* **9**, 2001243 (2020).

[16] Ruiz De Galarreta, C. et al. Tunable optical metasurfaces enabled by chalcogenide phase-change materials: from the visible to the THz. *J. Opt.* **22**, 114001 (2020).

[17] Cotrufo, M. et al. Reconfigurable image processing metasurfaces with phase-change materials. *Nat. Commun.* **15**, 4483 (2024). 38802353 10.1038/s41467-024-48783-3 PMC11130277

[18] Zhuo S. et al. Dynamic transmissive metasurface for broadband phase-only modulation based on phase-change materials. *Laser Photonics Rev.* **17**, 2200403 (2022).

[19] Li C. et al. Phase change materials-based bilayer metasurfaces for near-infrared photonic routing. *Adv. Funct. Mater.* **34**, 2310626 (2023).

[20] Yang, Y., Forbes, A. & Cao, L. A review of liquid crystal spatial light modulators: devices and applications. *Opto-Electron. Sci.* **2**, 230026 (2023).

[21] Khonina S. N., Butt M. A., Kazanskiy N. L. A review on reconfigurable metalenses revolutionizing flat optics. *Adv. Opt. Mater.,* 12, 2302794 (2023).

[22] Chang, X. et al. Electrically tuned active metasurface towards metasurface-integrated liquid crystal on silicon (meta-LCoS) devices. *Opt. Express* **31**, 5378 (2023). 36823819 10.1364/OE.483452

[23] Xiao, S. et al. Active metamaterials and metadevices: a review. *J. Phys. D: Appl. Phys.* **53**, 503002 (2020).

[24] Tsilipakos O. et al. Toward intelligent metasurfaces: the progress from globally tunable metasurfaces to software-defined metasurfaces with an embedded network of controllers. *Adv. Opt. Mater.* **8**, 2000783 (2020).

[25] Shalaginov, M. Y. et al. Design for quality: reconfigurable flat optics based on active metasurfaces. *Nanophotonics* **9**, 3505–3534 (2020).

[26] Abdelraouf, O. A. M. et al. Recent advances in tunable metasurfaces: materials, design, and applications. *ACS Nano* **16**, 13339–13369 (2022). 35976219 10.1021/acsnano.2c04628

[27] Bin-Alam, M. S. et al. Ultra-high-Q resonances in plasmonic metasurfaces. *Nat. Commun.* **12**, 974 (2021). 33579934 10.1038/s41467-021-21196-2 PMC7881010

[28] John-Herpin A. et al. Metasurface-enhanced infrared spectroscopy: an abundance of materials and functionalities. *Adv. Mater.* **35**, 2110163 (2022). 10.1002/adma.202110163 35638248

[29] Xu J. et al. Resonant scattering manipulation of dielectric nanoparticles. *Adv. Opt. Mater.* **9**, 2100112 (2021).

[30] Jeong, J. et al. High quality factor toroidal resonances in dielectric metasurfaces. *ACS Photonics* **7**, 1699–1707 (2020).

[31] Euvrard, J., Yan, Y. & Mitzi, D. B. Electrical doping in halide perovskites. *Nat. Rev. Mater.* **6**, 531–549 (2021).

[32] Dang P. et al. Recent advances in chromium-doped near-infrared luminescent materials: fundamentals, optimization strategies, and applications. *Adv. Opt. Mater.* **11**, 2201739 (2022).

[33] Chen X. et al. Graphene hybrid structures for integrated and flexible optoelectronics. *Adv. Mater.* **32**, 1902039 (2019). 10.1002/adma.201902039 31282020

[34] Jiang, H. et al. Design and simulation of a GST-based metasurface with strong and switchable circular dichroism. *Opt. Lett.* **47**, 1907 (2022). 35363766 10.1364/OL.448177

[35] Ding, X. et al. Tunable GST metasurfaces for chromatic aberration compensation in the mid-infrared. *Opt. Mater.* **109**, 110284 (2020).

[36] Qu, Y. et al. Thermal camouflage based on the phase-changing material GST. *Light. Sci. Appl.* **7**, 26 (2018). 30839556 10.1038/s41377-018-0038-5 PMC6107009

[37] Du, K.-K. et al. Control over emissivity of zero-static-power thermal emitters based on phase-changing material GST. *Light. Sci. Appl.* **6**, e16194-e (2016). 10.1038/lsa.2016.194 PMC6061892 30167194

[38] Li C. et al. Strong and omnidirectional light absorption from ultraviolet to near-infrared using GST metasurface. *Laser Photonics Rev.* **17**, 2200364 (2022).

[39] Chu, C. H. et al. Active dielectric metasurface based on phase-change medium. *Laser Photonics Rev.* **10**, 986–994 (2016).

[40] Hosseini, P., Wright, C. D. & Bhaskaran, H. An optoelectronic framework enabled by low-dimensional phase-change films. *Nature* **511**, 206–211 (2014). 25008527 10.1038/nature13487

[41] Wang, Y. et al. Electrical tuning of phase-change antennas and metasurfaces. *Nat. Nanotechnol.* **16**, 667–672 (2021). 33875869 10.1038/s41565-021-00882-8

[42] Hemmatyar O. et al. Electrically tunable phase-change metasurfaces using transparent conductive oxide microheaters[C]. CLEO: QELS_Fundamental Science. (Optica Publishing Group, 2021).

[43] Popescu, C. C. et al. Electrically reconfigurable phase-change transmissive metasurface. *Adv. Mater.* **36**, 2400627 (2024). 10.1002/adma.202400627 38724020

[44] Zhang, Y. et al. Electrically reconfigurable non-volatile metasurface using low-loss optical phase-change material. *Nat. Nanotechnol.* **16**, 661–666 (2021). 33875868 10.1038/s41565-021-00881-9

[45] Popescu C. C. et al. 2D Addressable Mid-infrared Metasurface Spatial Light Modulator. arXiv preprint arXiv:2511.03583, (2025).

[46] Bentata, F. et al. Spatially-controlled planar guided crystallization of low-loss phase change materials for programmable photonics. *Adv. Mater.* **38**, e06609 (2026). 41074657 10.1002/adma.202506609 PMC12879290

[47] Wei, M. et al. Monolithic back-end-of-line integration of phase change materials into foundry-manufactured silicon photonics. *Nat. Commun.* **15**, 2786 (2024). 38555287 10.1038/s41467-024-47206-7 PMC10981744

[48] Fang, Z. et al. Nonvolatile phase-only transmissive spatial light modulator with electrical addressability of individual pixels. *ACS nano* **18**, 11245–11256 (2024). 38639708 10.1021/acsnano.4c00340

[49] Yang, X. et al. Non-volatile optical switch element enabled by low-loss phase change material. *Adv. Funct. Mater.* **33**, 2304601 (2023).

[50] Li, S. Q. et al. Phase-only transmissive spatial light modulator based on tunable dielectric metasurface. *Science* **364**, 1087–1090 (2019). 31197013 10.1126/science.aaw6747

[51] Guo, T. et al. Durable and programmable ultrafast nanophotonic matrix of spectral pixels. *Nat. Nanotechnol.* **19**, 1635–1643 (2024). 39134690 10.1038/s41565-024-01756-5 PMC11567887

[52] Kim, J. Y. et al. Full 2π tunable phase modulation using avoided crossing of resonances. *Nat. Commun.* **13**, 2103 (2022). 35440594 10.1038/s41467-022-29721-7 PMC9018797

[53] Zhang, J. C. et al. Electromagnetic wave tailoring: from one dimension to multiple dimensions. *Electromagn. Sci.* **1**, 1–21 (2023).

[54] Li Y. R. et al. Lossless phase-change material enabled wideband high-efficiency spatial light phase modulation at near-infrared. *Laser Photonics Rev.* **18**, 400293 (2024).

[55] Huang, Y. S. et al. Optical phase change materials. *Annu. Rev. Mater. Res.* **55**, 255–283 (2025).

[56] Wei, M. et al. Electrically programmable phase-change photonic memory for optical neural networks with nanoseconds in situ training capability. *Adv. Photonics* **5**, 046004 (2023).

[57] Alam, M. S. et al. Fast cycling speed with multimillion cycling endurance of ultra-low loss phase change material (Sb2Se3) by engineered laser pulse irradiation. *Adv. Funct. Mater.* **34**, 2310306 (2024).

[58] Wen, J. et al. Deep learning-based miniaturized all-dielectric ultracompact film spectrometer. *Acs Photonics* **10**, 225–233 (2022).

[59] Zhang, H. et al. Snapshot computational spectroscopy enabled by deep learning. *Nanophotonics* **13**, 4159–4168 (2024). 39635447 10.1515/nanoph-2024-0328 PMC11501049

[60] Zeng S. et al. Source data for pixelated electrically driven Sb2Se3 phase-change metasurfaces. *Figshare*10.6084/m9.figshare.30093232 (2026). 10.1038/s41467-026-73199-6 PMC13402750 42168188

