# Supplementary material for: Pixelated electrically driven Sb2Se3 phase-change metasurfaces
Source: Nat Commun. 2026 May 21;17:7298. doi: 10.1038/s41467-026-73199-6 (PMC13402750; doi:10.1038/s41467-026-73199-6)
Supplement: Supplementary file 1 — Supplementary Information [file 41467_2026_73199_MOESM1_ESM.pdf]

Supplementary Information

Pixelated Electrically Driven Sb_2Se_3 Phase-Change Metasurfaces

Authors: Siqing Zeng[1], Yuru Li[1,7], Luoyao Chu[1,8], Ruifeng Zhong[1], Annan Zhao[1], Yan Li[2]*, Shunyu Yao[1,9], Xiaojie Zeng[1], Xiaoqi He[1], Tao Zhang[1], Zhaohuan Ao[1], Zhihao Fu[1], Zhaohui Li[1,3,4]*, Chao Lu[5] and Din Ping Tsai[6*]

[1]Guangdong Provincial Key Laboratory of Optoelectronic Information Processing Chips and Systems, School of Electrical and Information Technology, Sun Yat-sen University, Guangzhou 510275, China
[2]School of Microelectronics Science and Technology, Sun Yat-sen University, Zhuhai 519000, China
[3]Southern Marine Science and Engineering Guangdong Laboratory (Zhuhai), Zhuhai 519000, China
[4]IV-VI PIC Technology Co., Ltd, Hangzhou 311400, China
[5]Photonics Research Institute, Department of Electronic and Information Engineering, The Hong Kong Polytechnic University, Hong Kong, SAR, China
[6]Department of Electrical Engineering, City University of Hong Kong, Hong Kong 999077, China
[7]Present address: School of Optoelectronic Science and Engineering, South China Normal University, Guangzhou, China
[8]Present address: State Key Laboratory of Photonics and Communications, Department of Electronic Engineering, Shanghai Jiao Tong University, Shanghai 200240, China
[9]Present address: Institute of Semiconductors, Guangdong Academy of Sciences, Guangzhou 510075, China

These authors contributed equally: Siqing Zeng, Yuru Li, Luoyao Chu.

Correspondence to Yan Li (liyan329@mail.sysu.edu.cn), Zhaohui Li (lzhh88@mail.sysu.edu.cn) or Din Ping Tsai (dptsai@cityu.edu.hk).

Supplementary Note 1. Thermal simulation comparison between all-Au and Ti/Au heater designs

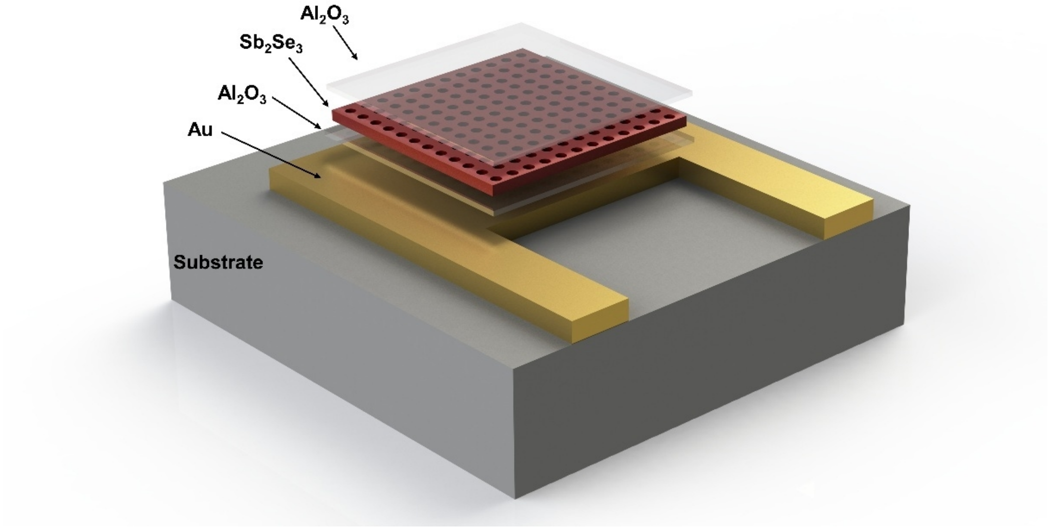

Supplementary Figure 1. 3D schematic of the all-Au micro-heater integrated with the Sb_2Se_3 metasurface pixel.

In this section, we present a comparative electro-thermal analysis of the Au/Ti bilayer micro-heater employed in our work and a conventional all-Au heater. Finite-element simulations were performed using the COMSOL Multiphysics software suite to evaluate the temperature distribution within a single, representative pixel during electrical crystallization and amorphization pulses. While the simulation models an individual pixel rather than the full array, it provides sufficient insight into the fundamental heating efficiency and thermal profiles of the two designs. For a fair comparison, the pixel dimensions were kept consistent: a central rectangular heater area of 85 µm × 150 µm, with 32-µm-wide extending leads, supporting a 75 µm × 75 µm Sb_2Se_3 metasurface atop its central region (structural schematic in Supplementary Figure 1).

The simulated temperature distributions under applied electrical pulses are shown in Supplementary Figure 2. For the Au/Ti bilayer design (Supplementary Figure 2a, b), the substantial resistivity contrast between the Au leads and the Ti pad effectively confines Joule heating to the central Ti region directly beneath the metasurface. The lead regions exhibit negligible temperature rise, confirming that electrical power is localized efficiently to drive the phase change. In contrast, for the all-Au heater (Supplementary Figure 2c, d), the narrow lead

width relative to the broad heater pad results in substantial Joule heating along the interconnect traces rather than within the target region beneath the metasurface. As a result, a substantial portion of the electrical power is dissipated wastefully in the leads. Although simulations confirm that a 2.5 V, 50 µs pulse can elevate the metasurface region to the crystallization temperature (~473.15 K), the lead temperature simultaneously approaches the melting point of gold (~1064 °C), posing a serious risk of thermal damage during operation. This issue becomes even more pronounced during the amorphization process: the simulation reveals that the lead temperature already nears the gold melting threshold before the metasurface region reaches the required amorphization temperature (~893.15 K) [1]. Consequently, the all-Au design fails to provide a reliable and efficient pathway for reversible phase switching, particularly for the amorphization operation. These findings demonstrate that the all-Au heater suffers from both prohibitive power inefficiency and fundamental limitations in achieving controlled amorphization. Therefore, the Au–Ti bilayer architecture was adopted in this work to ensure effective thermal localization, improved energy efficiency, and reliable phase-change switching.

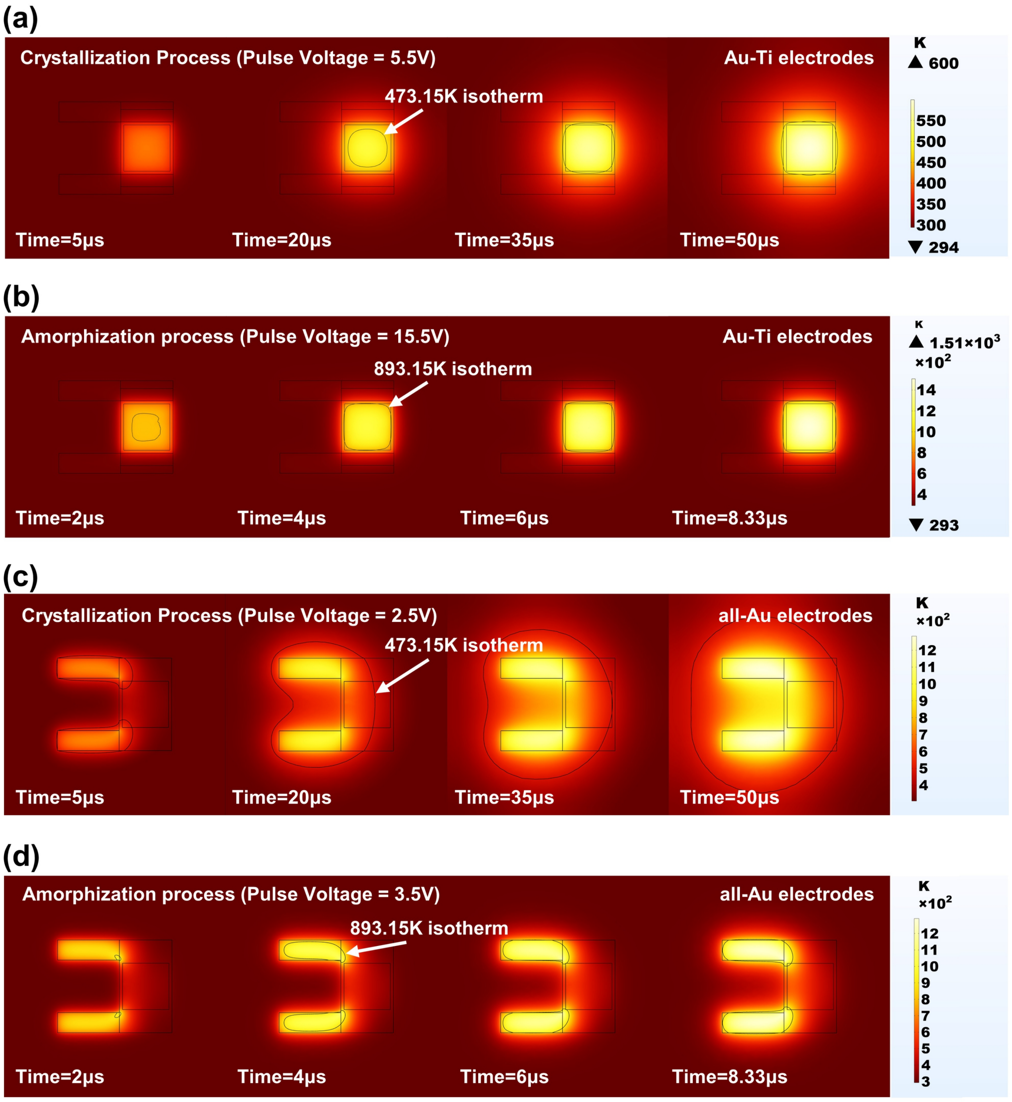

Supplementary Figure 2. Comparative thermal simulations of the Ti/Au and all-Au micro-heaters. Temperature distributions during (a) crystallization and (b) amorphization for the Ti/Au design, and during (c) crystallization and (d) amorphization for the all-Au design.

It is also important to note a key limitation of our current Au/Ti design: substantial thermal non-uniformity, or a central "hot spot," is observed within the Ti pad (Supplementary Figure 2a, b). This inhomogeneity is particularly pronounced during the high-power, short-duration amorphization pulse. For instance, under a 15.5 V, 8.33 μs amorphization pulse, while the entire Ti pad exceeds the required amorphization temperature of Sb_2Se_3 (~893.15 K), the central region reaches nearly 1500 K. This thermal gradient is a critical factor limiting the uniformity and controllability of the phase transition in the Sb_2Se_3 metasurface. Prior studies have proposed geometric optimization of heater shapes to improve thermal uniformity [2], which represents a promising direction for future refinement of our platform.

Supplementary Note 2. Design and thermal simulation of the row-column addressed heater array

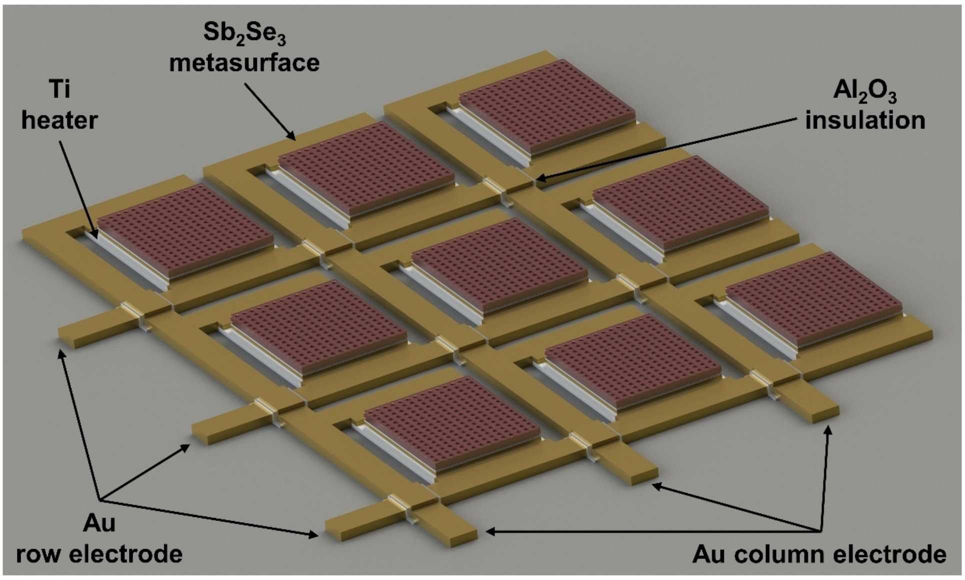

Supplementary Figure 3. Schematic of the stacked architecture: a row–column addressed micro-heater integrated with a Sb_2Se_3 metasurface array.

As illustrated in Supplementary Figure 3, we designed a conceptual stack employing a Ti/Au scheme analogous to our current work: Ti as the heating pad and Au as the interconnect lines. This row-column architecture consists of a bottom Ti electrode layer, an intermediate row/column electrode layer (insulated by a 10-nm Al_2O_3 layer between the row and column electrode layer), and a top Al_2O_3 insulation layer, over which the hybrid Sb_2Se_3 metasurface is placed.

To assess the feasibility of the proposed vertically stacked, row-column addressing scheme for electrically driving Sb_2Se_3 metasurface arrays, we performed numerical electro-thermal simulations using COMSOL Multiphysics. Although a modest 3×3 array was modeled for computational efficiency, the architecture inherently supports straightforward scaling to larger formats. The simulation investigated both a central and a corner pixel to account for potential positional variations in thermal response.

As shown in Supplementary Figure 4, when appropriate row and column lines are selected and driven with optimized voltage pulses, such as a 7.5 V, 50 μs pulse for crystallization and a 15 V, 8.33 μs pulse for amorphization, the temperature of the targeted pixel, whether central or corner, rises sufficiently to reach the required crystallization (~473.15 K) and amorphization (~893.15 K) thresholds, with heating highly localized to the addressed site [1]. The temperature increase in neighboring pixels arises primarily from thermal diffusion and remains well below the crystallization point in both switching regimes. Specifically, during crystallization pulses, adjacent pixel temperatures stay well below 473.15 K. During the higher-power amorphization pulses, although neighboring pixels experience some heating via thermal spreading, their temperatures

also remain below the crystallization threshold, thereby preserving their phase states. These results confirm that the row-column addressed heater can selectively switch individual pixels without unintentionally affecting their neighbors, demonstrating its fundamental suitability for pixel-independent phase control.

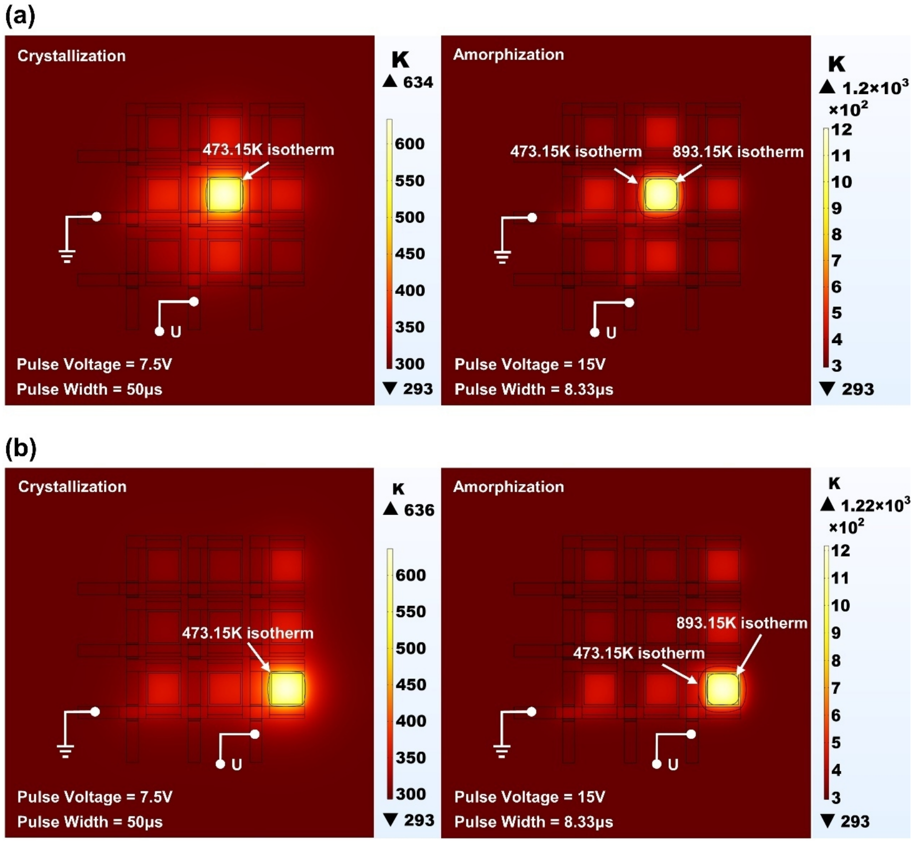

Supplementary Figure 4. Independent thermal addressing of pixels in a 3×3 row-column array. Simulated temperature profiles when applying (a) crystallization (7.5 V, 50 μs) and amorphization (15 V, 8.33 μs) pulses to the center pixel, and (b) the same pulses applied to a corner pixel.

Moreover, compared to the planar-wiring scheme used in our current experimental array, the row-column architecture decouples fill factor from array size. In our simulated design, a fill factor of ~50% was readily achieved without dedicated optimization, and further improvement is attainable through layout refinement. This addresses the critical trade-off between fill factor and scalability inherent in planar routing.

Overall, our simulations validate the row-column addressing scheme as a promising route toward scalable, high-fill-factor, and independently controllable phase-change metasurface arrays. However, we also acknowledge that the row-column configuration involves a more complex, multi-layer fabrication process (requiring multiple lithography and alignment steps) compared to the single-layer planar wiring used in our present proof-of-concept demonstration. Therefore, while the row-column approach holds great promise for future high-density, large-scale arrays, we employed the more readily fabricated planar wiring in this initial work to successfully demonstrate the core principle of randomly accessible, electrically driven phase-change metasurfaces.

Supplementary Note 3. Complete fabrication process flow of the electrically driven Sb_2Se_3 phase-change metasurface array

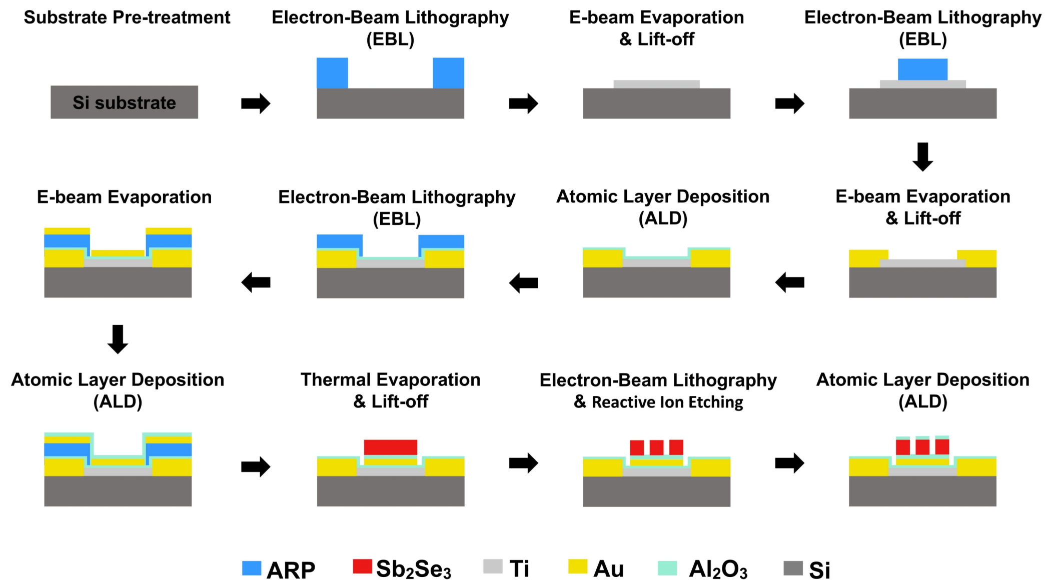

Supplementary Figure 5. Schematic of the fabrication process for electrically driven Sb_2Se_3 phase-change metasurface array.

The fabrication process flow of electrically driven Sb_2Se_3 phase-change metasurface array is shown in Supplementary Figure 5. First, the silicon substrate was pre-cleaned and treated by sequentially immersing in acetone, isopropanol, and deionized water, followed by ultrasonic cleaning at room temperature for 30 minutes to remove any existing impurities. The substrate was then dried using a hot plate. Next, a spin coater was used to apply photoresist onto the substrate, which was subsequently exposed to electron beam lithography (EBL), followed by development. Afterward, a 70 nm thick titanium layer was deposited via electron beam evaporation, and the unwanted titanium was removed using a liff-off process, forming the titanium heating layer. Subsequently, a similar stripping process was employed to form the electrode wire, including spin coating of photoresist, electron beam exposure, and development of the photoresist, followed by the deposition of a 900 nm thick gold layer via electron beam evaporation and lift-off to remove the unwanted photoresist. A 10 nm thick aluminum oxide (Al_2O_3) was then deposited on top of the electrode for electrical insulation.

The fabrication of the top-layer hybrid phase-change metasurface relies on the combination of multilayer lift-off and the reactive ion etching of Sb_2Se_3 microstructures. Initially, the pattern of the metasurface pixels was defined on the top of the titanium electrode using EBL and development. Subsequently, a 50-nm-thick gold reflective layer, a thin Al_2O_3 dielectric insulating layer, and an Sb_2Se_3 film were sequentially deposited. The residual photoresist in the surrounding areas was then removed through a lift-off process. Next, the pattern corresponding to the micropores array was defined on the Sb_2Se_3 film using EBL, followed by plasma etching and the

removal of residual photoresist using an oxygen plasma treatment, to complete the fabrication of the phase-change micropores array. Finally, a 30-nm-thick Al_2O_3 protective layer was deposited on the fabricated metasurface array via atomic layer deposition to mitigate potential deformations in the Sb_2Se_3 microstructures during the phase-change process.

Supplementary Note 4. Design and simulation of the phase-change meta-atom

This section presents the design and simulated optical response of under-coupled and over-coupled phase-change metasurface based on Sb_2Se_3 micropores array. The commercial simulation software Lumerical FDTD was employed to compute the reflection spectra and the electromagnetic field distributions within the meta-atom. During the simulation, the dielectric constants of Si, Al_2O_3, and Au were sourced from the software's built-in database: Si-Palik, Al_2O_3-Palik, and Au-CRC. The dielectric constants of amorphous and crystalline Sb_2Se_3 were obtained from self-measured data in our laboratory. During the phase transition, the intermediate phase state of Sb_2Se_3 was treated as a homogeneous material with a dielectric constant between the amorphous and crystalline states, commonly represented by the Lorentz-Lorenz equation, given as [2]

$$\frac{\epsilon_{eff}(\lambda)-1}{\epsilon_{eff}(\lambda)+2}=m\times\frac{\epsilon_c(\lambda)-1}{\epsilon_c(\lambda)+2}+(1-m)\times\frac{\epsilon_a(\lambda)-1}{\epsilon_a(\lambda)+2} \quad (1)$$

Here, $\epsilon_a(\lambda)$ and $\epsilon_a(\lambda)$ denote the complex permittivity of the amorphous and crystalline Sb_2Se_3 at wavelength λ, m denotes the crystallization ratio, and $\epsilon_{eff}(\lambda)$ is the effective complex permittivity corresponding to an intermediate phase state.

The simulated reflection and phase spectra of the undercoupled Sb_2Se_3 micropore-array phase-change metasurface during the phase-transition process are shown in Supplementary Figure 6a and 6b. In the amorphous state, the Sb_2Se_3 micropore structure exhibits pronounced resonant absorption near 1480 nm, accompanied by a phase jump, indicating that the current resonant mode is in an undercoupled state. As the crystallization ratio increases, the resonant mode experiences pronounced redshift in frequency, and the reflectance at the resonance center wavelength remains close to zero, indicating that the resonant mode maintains an undercoupled state near critical coupling throughout the phase transition process. This phenomenon is attributed to the strong confinement of the resonant mode's electric field within the gaps of the adjacent micropore structures, as shown in Supplementary Figure 6c and 6d. Due to the intrinsic absorption characteristics of the underlying gold reflective layer in the near-infrared band, the internal dissipation rate γ_d remains exceeds radiation coupling rate γ_s. The frequency shift of the undercoupled resonant mode introduces amplitude modulation across the scanned wavelength range. However, it is important to note that, due to the pronounced optical contrast of the phase-change material, adjacent higher-order resonant modes also sweep through the operating wavelength during the phase transition, resulting in the nonlinear amplitude modulation shown in Supplementary Figure 6e. To address this issue, during the experimental process, the phase-change material was intentionally maintained within a weakly crystallized range by controlling the crystallization pulse voltage to avoid the influence of adjacent resonant modes. Moreover, prior studies have shown that a lower degree of crystallization also helps to improve the durability of the phase-change material-[3]. By comparing the center wavelengths of the resonant modes after electrically driven crystallization of the metasurface, it can be found that, as shown in Supplementary Figure 6f, the crystallization ratio m reaches approximately 0.4.

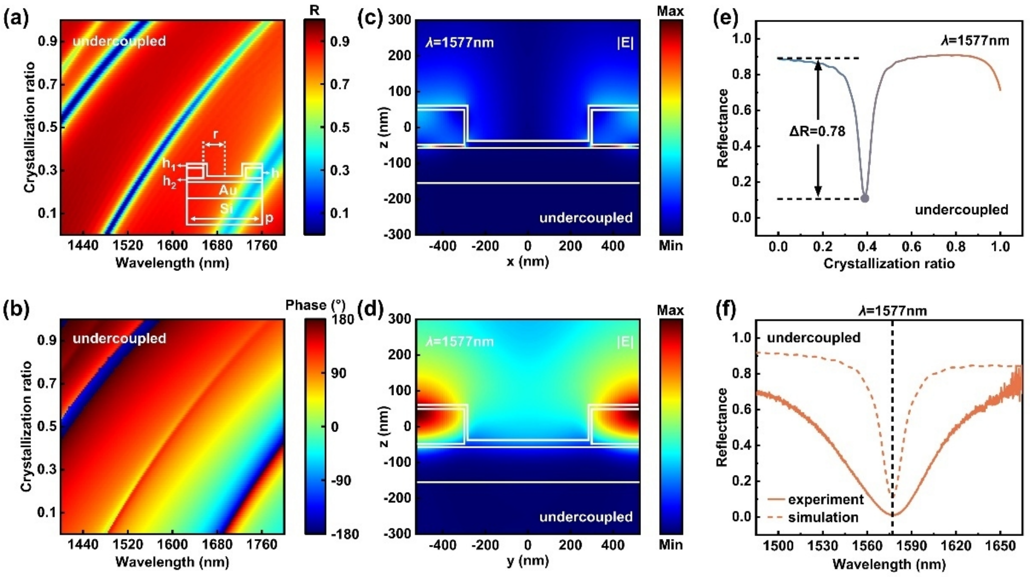

Supplementary Figure 6. Simulated optical response of the undercoupled Sb_2Se_3 metasurface. a, Simulated reflection spectra of the undercoupled Sb_2Se_3 metasurface at different crystallization ratios. The inset illustrates the geometric parameters of the meta-atom. Here, $r = 300$ nm denotes the diameter of the micropore, $h = 100$ nm is the height of the micropore, $h_1 = 10$ nm is the thickness of the Al_2O_3 protective layer on top of the micropore, $h_2 = 5$ nm is the thickness of the Al_2O_3 isolation layer between the phase-change micropore and the gold reflective layer, and $p = 1050$ nm is the period of the micropore array. b, Simulated reflection phase spectra of the undercoupled metasurface as a function of crystallization ratio. c, Distribution of the electric field intensity of the resonance mode within the x-z cross-section of the meta-atom at the resonant center wavelength. d, Distribution of the electric field intensity of the resonance mode within the y-z cross-section of the meta-atom. e, Simulated amplitude modulation at the wavelength of 1577 nm as a function of crystallization ratio. f, Comparison between the simulated reflection spectrum of the metasurface at $m = 0.4$ and the experimentally measured reflection spectrum under weakly crystallized conditions.

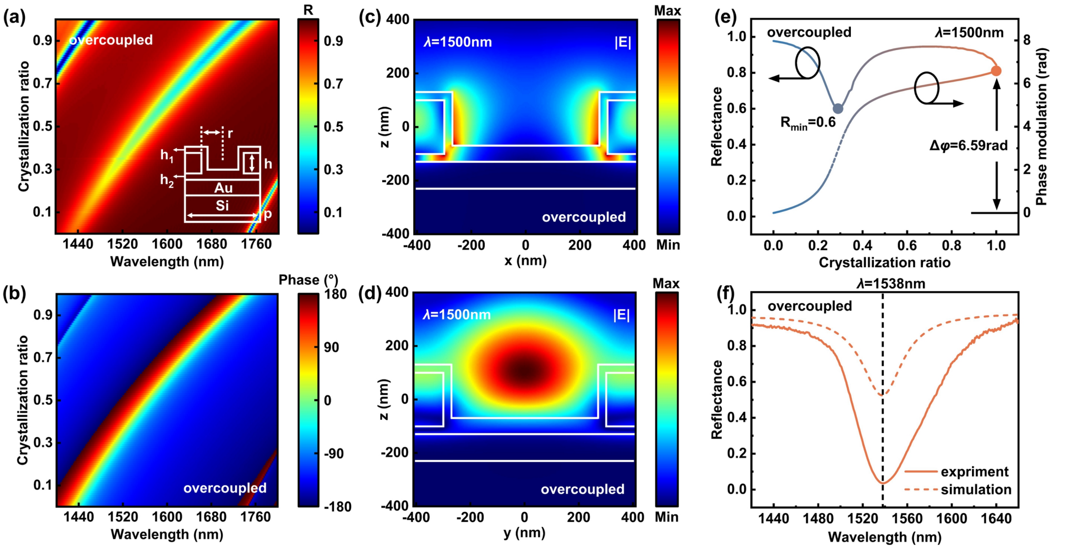

Supplementary Figure 7. Simulated optical response of the overcoupled Sb_2Se_3 metasurface. a, Simulated reflection spectra of the overcoupled Sb_2Se_3 metasurface at different crystallization ratios. The inset illustrates the geometric parameters of the meta-atom. Here, $r = 300$ nm denotes the diameter of the micropore, $h = 200$ nm is the height of the micropore, $h_1 = 30$ nm is the thickness of the Al_2O_3 protective layer on top of the micropore, $h_2 = 30$ nm is the thickness of the Al_2O_3 isolation layer between the phase-change micropore and the gold reflective layer, and $p = 820$ nm is the period of the micropore array. b, Simulated reflection phase spectra of the overcoupled metasurface as a function of crystallization ratio. c, Distribution of the electric field intensity of the resonance mode within the x-z and y-z (d) cross-section of the meta-atom at the resonant center wavelength. e, Simulated amplitude and phase variations at the wavelength of 1500 nm as a function of crystallization ratio. f, Comparison between the simulated reflection spectrum of the metasurface at $m = 0.28$ and the experimentally measured reflection spectrum under weakly crystallized conditions.

The meta-atom design and simulated optical response of the strongly overcoupled phase-change metasurface are shown in Supplementary Figure 7a and 7b. The current resonance mode introduces an additive phase modulation covering over a full 2π range around the center wavelength, accompanied by a modest variation in reflection amplitude, indicating a strongly overcoupled state. As shown in the electric field distributions of the resonance mode in Supplementary Figure 7c and 7d, the electric field is predominantly confined within the air environment at the center of the micropore, away from the underlying metallic layer. Thus, the intrinsic absorption loss of the resonance mode is much lower than the radiation loss, maintaining the strongly overcoupled state throughout the phase-change process. As the material undergoes phase transition, the frequency shift of the resonance mode provides phase dominant modulation across the swept wavelength range, as shown in Supplementary Figure 7e. It is important to note that, during the experiment, this overcoupled phase-change metasurface was also confined within a weakly crystallized range by controlling the crystallization pulse voltage. A comparison between the experimentally measured reflection spectra of the crystallized metasurface and the simulation results, as shown in Supplementary Figure 7f, reveals that the actual crystallization ratio of the metasurface is approximately 0.28. In addition to ensuring the cyclic characteristics of the

phase-change metasurface, previous studies have indicated that Sb_2Se_3 exhibits a certain degree of non-uniform refractive index distribution during crystallization. Strongly crystallized Sb_2Se_3 can induce unexpected energy scattering, thereby affecting the efficiency of phase modulation [4]. Therefore, although the restriction of the crystallization ratio would sacrifice a certain degree of phase modulation range, the current work still recommends a weakly crystallized phase-change working range.

Supplementary Note 5. Experimental setup for characterizing the reflection and phase spectra of phase-change metasurfaces

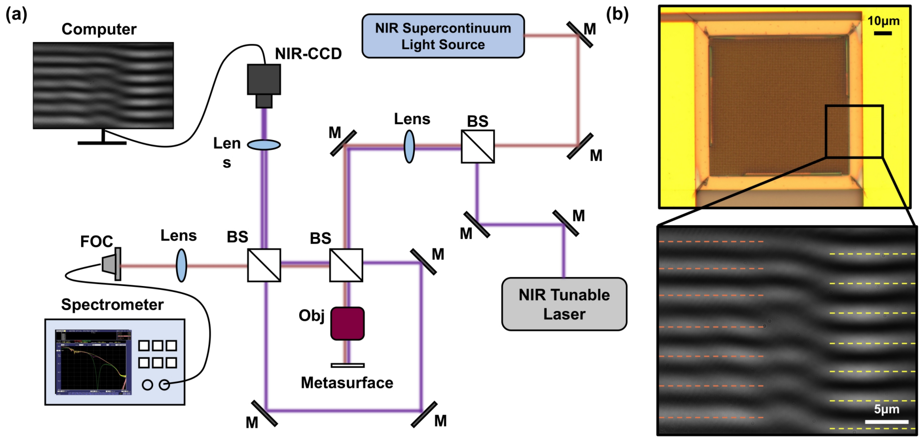

Supplementary Figure 8. Measurement system and method for reflection and phase spectra of phase-change metasurfaces. a, Schematic diagram of the experimental system for measuring the reflection spectrum and phase response. b, The interference patterns of the metasurface captured by the NIR camera during the phase calibration.

The optical performance of the Sb_2Se_3 phase-change metasurface was characterized using a customized NIR micro-spectral measurement system, as illustrated in Supplementary Figure 8. The system consists of two key modules: a reflection spectra measurement module and a phase response measurement module, providing a comprehensive analysis of the metasurface's optical properties. For the reflection spectrum measurement, a NIR supercontinuum light source (Fianium WhiteLase) was employed, with light focused onto the metasurface using a 20× objective lens. The metasurface sample was mounted vertically on an adjustable platform to ensure its surface was perpendicular to the incident light direction. Reflected signals were collected by the same objective lens and directed into a spectrometer (Yokogawa AQ6370D) for spectra analysis. For phase spectra measurement, a tunable laser (Keysight 8164B) served as the light source. Here, the measurement of the reflection phase of the Sb_2Se_3 metasurface during the phase transition refers to the interferometric method employed in previous works [5]. The incident tunable laser is split by a beam splitter cube. One portion of the light is focused onto the metasurface via an objective lens, reflected by the metasurface, and then collected by the same objective lens before being directed towards the CCD. The other portion of the light is directly reflected towards the camera, forming an interference pattern with the reflected beam from the metasurface. During the measurement, the laser irradiation area is confined to the edge of the metasurface, as shown in Supplementary Figure 8b. By comparing the relative displacement of the interference fringes between the metasurface and the surrounding electrode region, the relative phase difference between the metasurface and the surrounding electrode region, $\Delta\phi$, can be obtained. Evidently, the reflection phase of the surrounding electrode region remains stable during the phase transition. Therefore, the reflective phase modulation of the metasurface during the phase transition can be directly determined by measuring the variation in the phase difference $\Delta\phi$.

Supplementary Note 6. Extended analysis of cycling endurance in electrically driven Sb_2Se_3 metasurfaces

This section provides a detailed investigation into the cycling endurance of our electrically driven Sb_2Se_3 metasurface, extending the discussion from the initial results presented in Figure 2g of the main text. We examine the mechanisms limiting cycle life, beginning with the failure observed under high-voltage driving. As shown in Supplementary Figure 9, optical microscopy of the device after approximately 60 cycles under high driving voltages (5.3 V for crystallization, 14 V for amorphization) reveals a physical fracture at the Au-Ti electrode interface. This mechanical failure is attributed to thermomechanical stress induced by the high-power pulses.

To confirm that this failure was confined to the heater and did not reflect degradation of the phase-change material itself, we performed post-failure optical characterization using a laser-assisted switching platform, with the method detailed in our previous work [4]. The reflection spectra of the same metasurface pixel in different phase states are shown in Supplementary Figure 10. The black curve corresponds to the electrically crystallized state obtained prior to heater fracture, whereas the orange and blue curves represent the states achieved through subsequent laser-induced amorphization and re-crystallization. The successful reversal of the phase transition confirms that the Sb_2Se_3 metasurface retains its intrinsic functionality, isolating the heater as the point of failure.

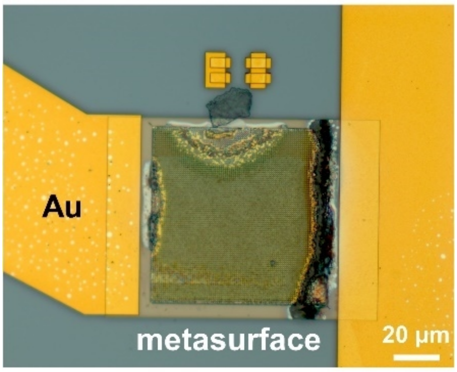

Supplementary Figure 9. Optical micrograph of the fractured micro-heater after 60 cycles.

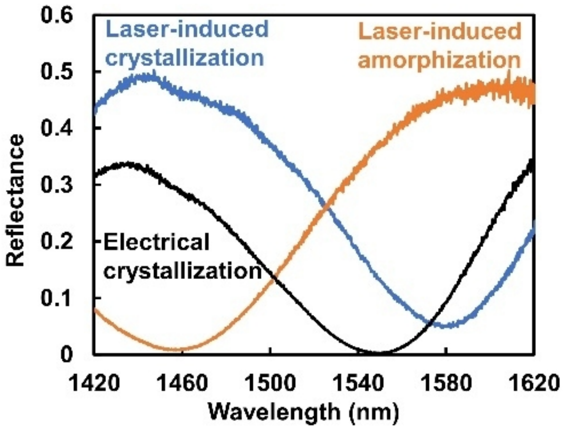

Supplementary Figure 10. Phase-change functionality before and after electrode

fracture. Reflection spectra after: electrical crystallization (before fracture, black); laser-induced amorphization (immediately after fracture, orange); and subsequent laser-induced crystallization (blue), demonstrating retained metasurface operability.

To probe the intrinsic cycling limits while avoiding mechanical failure, we evaluated a modified device under lower driving voltages (4.7 V for crystallization, 13 V for amorphization). The reflectance at 1412 nm was monitored over more than 850 switching cycles, as plotted in Supplementary Figure 11a. The device maintained reversibility without electrode damage, indicating a markedly improved operational lifetime under these conditions. However, a gradual decay in the optical modulation depth was observed. Further analysis of the spectral evolution offers insight into this decay. Supplementary Figure 11b and c compare the reflection spectra from the first 30 and the final 30 cycles, respectively. The data show a progressive redshift of the resonance in the re-amorphized state, while the crystalline-state resonance remains stable. This indicates a gradual reduction in amorphization efficiency. Such a decay in optical contrast is commonly observed in various phase-change photonic devices and has often been attributed to cumulative morphological changes in the active material during cycling [1]. However, we note that in our case, morphological change is unlikely to be the primary cause. The remarkable stability of the crystalline-state resonance throughout the test suggests that the overall metasurface geometry is preserved. The degradation is isolated to the amorphization process. This observation, combined with our electro-thermal simulations presented in Supporting Information S2, which reveal pronounced heating non-uniformity specifically under the short, high-power pulses required for amorphization, leads us to attribute the gradual decay primarily to increasingly incomplete re-amorphization. We hypothesize that localized hotspots progressively alter the phase-change kinetics over many cycles, reducing the completeness and uniformity of the amorphized state formed under a fixed electrical pulse.

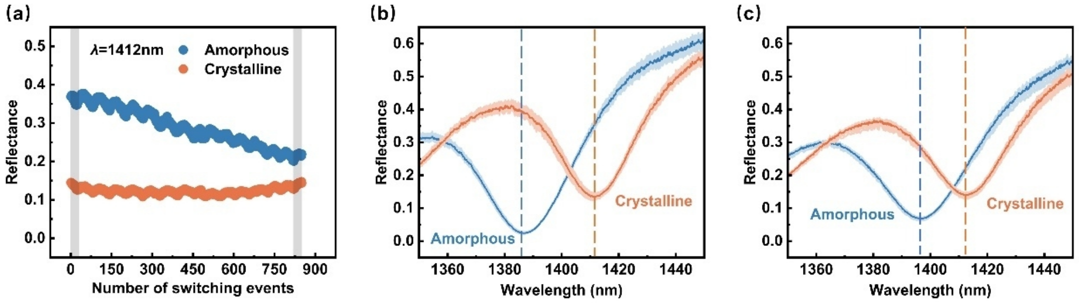

Supplementary Figure 11. Cycling endurance of the Sb_2Se_3 metasurface under low-voltage electrical driving. (a) Evolution of reflectance at 1412 nm over 850 switching cycles driven by 4.7 V, 1 s (crystallization) and 13 V, 8.33 μs (amorphization) pulses. Corresponding reflection spectra for (b) the first 30 cycles and (c) the last 30 cycles.

The cycle counts reported here are not fundamental limits of the Sb_2Se_3 material, as prior work in integrated photonic platforms has demonstrated stable electrothermal cycling exceeding 10^4 times for similar phase-change systems. The performance of our present platform is therefore constrained primarily by heater reliability and thermal management. Future optimizations of the heater geometry for improved mechanical robustness and temperature uniformity are expected to enable cycling performance that approaches the intrinsic endurance of the phase-change material.

Supplementary Note 7. Reflectance contrast calculation for phase-change metasurfaces during validation of high-speed electrically-driven phase transition

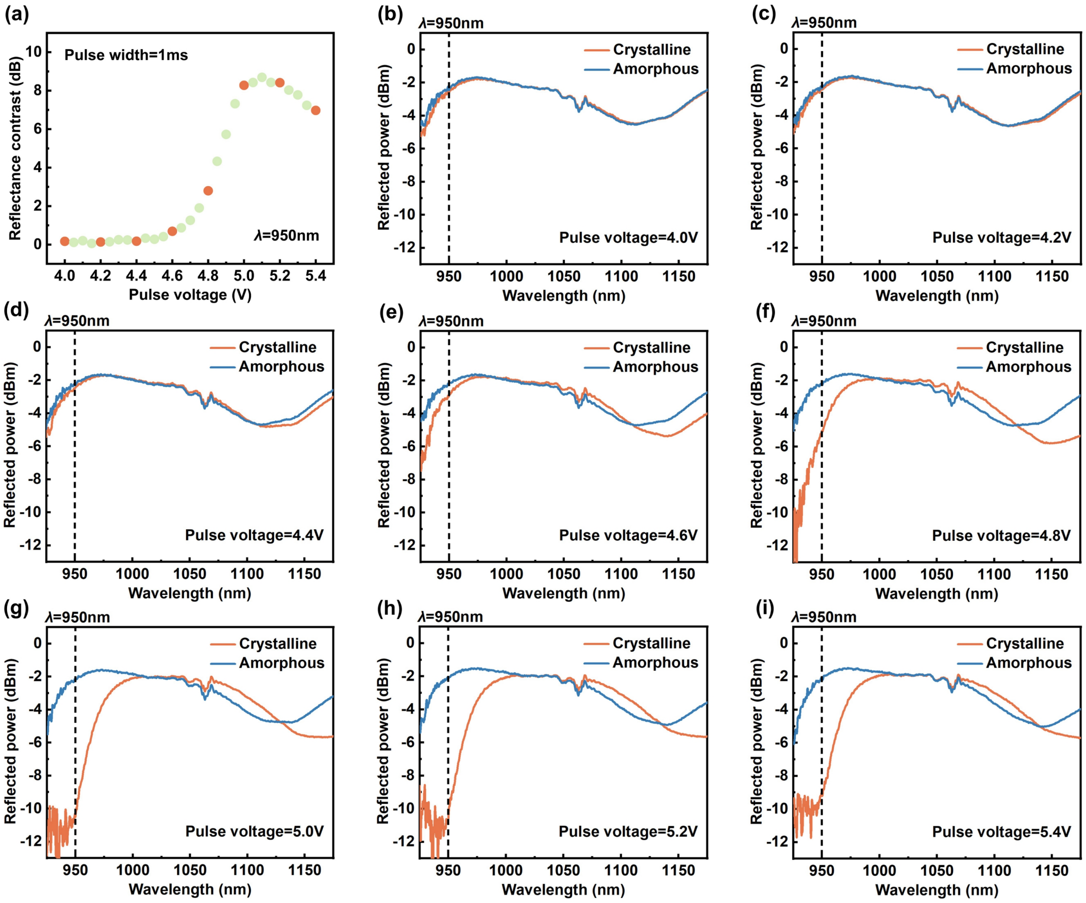

Supplementary Figure 12. Calculation of reflectance contrast to characterize the phase-chance ratio of metasurfaces. a, Variation of the metasurface reflectance contrast as a function of pulse voltage for crystallization at a fixed pulse width of 1 ms. b-i, Measured reflectance power spectra of the metasurface under different crystallization voltages compared with those in the amorphous state.

To investigate the response speed of the electrically-driven crystallization of Sb_2Se_3 metasurfaces, this study examined the crystallization behavior of the metasurfaces under various pulse widths. The degree of crystallization was quantified by the reflectance contrast at the wavelength of 950 nm, measured before and after the phase transition. As shown in Supplementary Figure 12, the reflectance power spectra of the metasurfaces before and after crystallization under different drive voltages, with a fixed pulse width of 1 ms, are presented as an example. With increasing crystallization pulse voltage, the reflectance power spectrum of the metasurfaces exhibits a gradual redshift in resonances, indicative of an increasing crystallization ratio of Sb_2Se_3. During the crystallization, one of the resonance modes' center wavelengths continuously approaches 950

nm, introducing an amplitude modulation effect that increases with the degree of crystallization. Consequently, the reflectance contrast at 950 nm before and after crystallization can intuitively represent the degree of crystallization of the phase-change material. Notably, as the degree of crystallization further increases, the reflectance contrast reaches a maximum value and then experiences a slow decline. However, the reflectance power spectrum reveals that the center wavelength of the resonance mode does not exhibit further redshift, indicating that the phase-change material has nearly fully crystallized and does not show further improvement with increasing voltage. This work attributes the reduction in modulation depth of the metasurfaces under high voltage to the possible degradation of the phase-change material. Therefore, in subsequent comparisons, a modest modulation depth of 3 dB was selected as the standard to investigate the crystallization behavior of the electro-controlled metasurfaces under different crystallization pulse widths.

Supplementary Note 8. Experimental process of spatial spectral reconstruction based on phase-change metasurface array

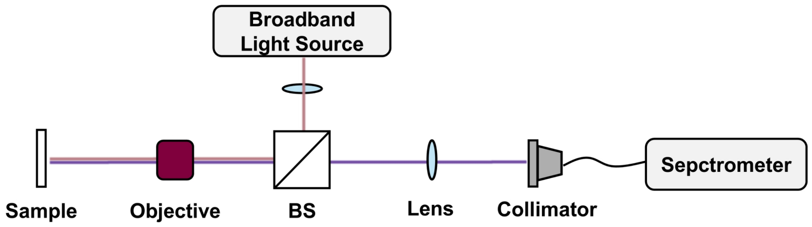

Supplementary Figure 13. Schematic of the metasurface array transmission matrix calibration system.

The complete spectral reconstruction process can be broadly divided into three steps. First, the spectral transmission matrix is calibrated to obtain the spectral response curves corresponding to different metasurface structure units over the entire working wavelength range. In this work, this is achieved by measuring the reflected power spectrum of each metasurface unit at different wavelengths and constructing a complete spectral transmission matrix, which provides the foundation for subsequent spectral reconstruction. Next, the neural network model is trained, using the compressed spectral response data as input to learn the mapping relationship between the reflected intensity of individual units in the metasurface array and the incident spectrum, ultimately resulting in a reconstruction model with generalization capability. Finally, the experimental spectral reconstruction and validation step involves illuminating the metasurface array with sample light source, using the model to predict the corresponding spectra, and comparing these predictions with results obtained from a commercial spectrometer to validate the reconstruction accuracy and system stability.

The first step involves calibrating the spectral transmission matrix $T_i(\lambda)$, and the experimental system is shown in Supplementary Figure 13. The broadband light source is reflected by a beam splitter and focused onto the metal electrode region of the metasurface array by an objective lens. The reflected light passes through the beam splitter again, is collimated, and then focused onto a commercial spectrometer to acquire the power spectrum under total reflection from the metallic layer as the reference. Subsequently, the broadband light source sequentially illuminates individual pixels of the metasurface array, and the reflected power spectrum of each pixel is captured. By dividing the reflected power spectrum of each metasurface unit by the reference spectrum, a complete spectral transmission matrix can be constructed.

Next, 200,000 sets of spectral data are numerically generated, with 10% used as the test set and 90% as the training set for the development of the neural network. A linear layer with fixed weights is used to compress the high-dimensional spectral data into 36 sets of photocurrent data I_i, which simulate the photocurrents actually measured in the experiment. These fixed weights are determined by the previously calibrated transmission matrix. The data is then passed through a weight prediction network and input into a six-layer fully connected network. The first four layers of the network use Dropout (p=0.01) and Batch Normalization to prevent overfitting and improve

training stability. The Dropout layers randomly drop a certain proportion of neurons (with a dropout probability of p=0.01), helping to prevent the neural network from becoming overly dependent on certain neurons, thus reducing the risk of overfitting. Batch Normalization standardizes the output of each layer, ensuring a mean of 0 and a variance of 1, which accelerates the training process, maintains stability in the inputs of each layer, and effectively mitigates issues such as gradient explosion or vanishing gradients. The activation function for each layer is LeakyReLU, providing nonlinear characteristics that enable the neural network to learn more complex features. The final layer of the network uses a Sigmoid activation function, which limits the output to a range between 0 and 1, normalizing the results and mapping them to a standardized range.

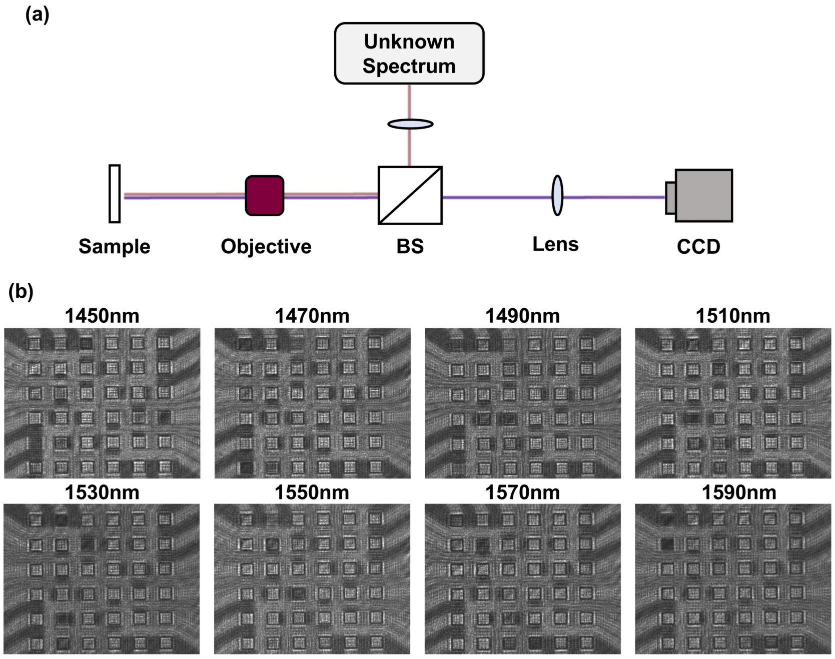

Supplementary Figure 14. Validation process of spatial spectral reconstruction. a, Schematic diagram of the experimental system for spatial spectral reconstruction verification. b, CCD-captured grayscale images of the metasurface array under single-wavelength illumination.

After the neural network is trained, spatial spectrum reconstruction experiments are conducted to verify its effectiveness. The experimental setup is shown in Supplementary Figure 14a. The overall structure is similar to the transmission matrix calibration system, with the main difference being the replacement of the light source with an unknown spectral source and the substitution of the commercial spectrometer with a CCD camera at the detection end. The grayscale images captured by this system, combined with the quantum efficiency of the CCD, can be mapped into the photocurrent signals I_i corresponding to the ith metasurface unit. Taking single-wavelength reconstruction as an example, as shown in Supplementary Figure 14b, under irradiation of incident light, each pixel in the metasurface array exhibits distinct reflected light intensity distributions. By inputting these reflective intensity patterns from the metasurface array into the trained neural network, accurate reconstruction of the incident light spectrum can be accomplished. This methodology remains equally effective for spectral reconstruction of broadband light sources.

Supplementary Note 9. Discussion on the cross-correlation of spectral transmission matrices

In spectral reconstruction, a lower correlation within the transmission matrix is beneficial for improving both reconstruction accuracy and system stability. Low correlation indicates that different metasurface structures exhibit highly distinct and complementary responses, providing richer spectral discrimination information. This reduces information redundancy and input ambiguity, thereby enhancing the neural network's ability to resolve diverse spectral features and ultimately improving the overall reconstruction performance. In previous works, the cross-correlation coefficient matrix is often employed to quantify the degree of correlation among the transmission responses of different metasurface units [6-8]. Assume the transmission matrix $T_{m\times n}$, where m is the number of wavelength sampling points and n is the number of metasurface units with distinct spectral response ($n = 36$ in this work). According to the definition of the Pearson correlation coefficient, the correlation coefficient between the spectral response of ith and jth metasurface unit can be calculated as

$$r_{ij}^{(\mathrm{col})} = \frac{\mathrm{cov}(T_{:,i}, T_{:,j})}{\sigma(T_{:,i})\sigma(T_{:,j})} \quad (2)$$

The cov represents the sample covariance, and σ represents the standard deviations. Based on this definition, the cross-correlation coefficient matrix of the amorphous Sb_2Se_3 metasurface array is calculated, as shown in Supplementary Figure 15.

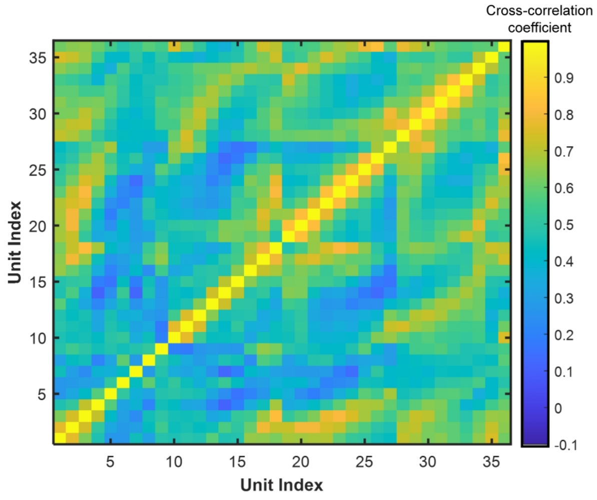

Supplementary Figure 15. Cross-correlation coefficient matrix of the 6×6 amorphous Sb_2Se_3 metasurface array.

It is noteworthy that the conventional method of calculating cross-correlation coefficients between spectral responses of metasurface units cannot comprehensively evaluate the potential of transmission matrices in spectral reconstruction. Specifically, an ideal narrowband filter array constructed with varying center wavelengths spanning a broad spectral range would exhibit minimal cross-correlation coefficients. However, such transmission matrices demonstrate competent spectral reconstruction capabilities only at specific wavelengths, failing to achieve broadband spectral recovery. From the operational principle perspective, spectral reconstruction

fundamentally requires transmission matrices to effectively sample target spectra. When considering broadband spectral reconstruction, an ideal transmission matrix should provide rich spectral responses at any wavelength within the operational range. This implies that unlike classical cross-correlation coefficient calculations, the spectral correlation coefficients between different wavelength positions could more directly reflect the potential of constructed transmission matrices for broadband spectral reconstruction. This concept is hereby defined as the spectral cross-correlation coefficient in the current work, given as

$$r_{ij}^{(\mathrm{row})} = \frac{\mathrm{cov}\left(T_{i,:}, T_{j,:}\right)}{\sigma(T_{i,:})\sigma(T_{j,:})} \tag{3}$$

In Figure 5b of the main text, we present the average spectral cross-correlation coefficients between wavelengths, calculated over the spectral range from 1050 nm to 1650 nm with a step size of 0.2 nm. Two strategies for increasing the complexity of the transmission matrix are compared: one involves progressively increasing the number of metasurface units with varying structural dimensions of meta-atom, while the other introduces phase-change induced spectral modulation. The results show that incorporating phase-change modulation substantially reduces the correlation, outperforming the approach of simply adding more units, and indicating greater potential for achieving higher spectral reconstruction accuracy.

Supplementary references

[1] Yang X, Lu L, Li Y, et al. Non-volatile optical switch element enabled by low-loss phase change material[J]. Advanced Functional Materials, 2023, 33(42): 2304601.

[2] Zhang Y, Fowler C, Liang J, et al. Electrically reconfigurable non-volatile metasurface using low-loss optical phase-change material[J]. Nature Nanotechnology, 2021, 16(6): 661-666.

[3] Gao K, Du K, Tian S, et al. Intermediate phase‐change states with improved cycling durability of Sb_2S_3 by femtosecond multi‐pulse laser irradiation[J]. Advanced Functional Materials, 2021, 31(35): 2103327.

[4] Li Y R, Li Y, Zeng S, et al. Lossless Phase-Change Material Enabled Wideband High-Efficiency Spatial Light Phase Modulation at Near-Infrared[J]. Laser & Photonics Reviews, 2024, 18(11): 2400293.

[5] Kafaie Shirmanesh G, Sokhoyan R, Pala R A, et al. Dual-gated active metasurface at 1550 nm with wide (> 300) phase tunability[J]. Nano letters, 2018, 18(5): 2957-2963.

[6] Wen J, Hao L, Gao C, et al. Deep learning-based miniaturized all-dielectric ultracompact film spectrometer[J]. Acs Photonics, 2022, 10(1): 225-233.

[7] Zhang H, Li Q, Zhao H, et al. Snapshot computational spectroscopy enabled by deep learning[J]. Nanophotonics, 2024, 13(22): 4159-4168.

[8] Bian L, Wang Z, Zhang Y, et al. A broadband hyperspectral image sensor with high spatio-temporal resolution[J]. Nature, 2024, 635(8037): 73-81.
