# Supplementary material for: Pixelated electrically driven Sb2Se3 phase-change metasurfaces
Source: Nat Commun. 2026 May 21;17:7298. doi: 10.1038/s41467-026-73199-6 (PMC13402750; doi:10.1038/s41467-026-73199-6)
Supplement: Supplementary file 2 — Transparent Peer Review file [file 41467_2026_73199_MOESM2_ESM.pdf]

nature portfolio

Peer Review File

Pixelated Electrically Driven Sb_2Se_3 Phase-Change Metasurfaces

Corresponding Author: Professor Yan Li

This file contains all reviewer reports in order by version, followed by all author rebuttals in order by version.

Version 0:

Reviewer comments:

Reviewer #1

(Remarks to the Author)
In their work, Zeng et al. demonstrate for the first time electrically driven, which they apply for amplitude and phase modulation of light in the near-infrared range as well as its spectral reconstruction. The work is a sound addition to the rapidly developing area of phase change tunable photonic structures. From that standpoint, I find the results appealing to a broad audience and meeting the high standards of Nature Communications. Still, there are a few critical points that in my opinion need to be addressed before the paper is accepted for publication.

The results need to be better positioned against the latest research on electrically controlled PCM optical devices. In particular, there is the recent paper by Popescu et al. Advanced Materials (2024) https://doi.org/10.1002/adma.202400627, which reports on a device with similar functionality based on GSST that also operates in the near-infrared range. In this context, I believe that the claim "this work establishes a novel paradigm for dynamic optical devices…" should be suspended from the abstract as not being true. Furthermore, while some of perks of using lower loss Sb2Se3 are rightfully mentioned by the authors, there are also additional advantages that can be highlighted (from what I can tell, lower switching voltages/energy consumption and no phase separation issues due to binary nature of the compound) and some potential tradeoffs that needs to be discussed in more detail.
The authors take additional effort to highlight the importance of decoupling of the heater electrodes with the "optical" part of the device (line 83; line 136). While I understand that the direct contact of titanium with the Sb2Se3 metasurface might deteriorate the optical performance, why did the authors not consider using gold-only electrodes? Does the use of titanium allow to optimize the spatial distribution of the power loss? This needs to be explained more thoroughly.
I found Figure 2 quite confusing, as it contains data that covers 3 different spectral ranges, making it quite difficult to compare and analyze. First, I did not immediately find the part where the authors explicitly address the much lower off-resonance reflectivity amplitude of the re-amorphized device (Fig. 2b, experiment); I believe that the only place that mentions it is line 216, which should be expanded further to explain the difference. Second, I was puzzled why panel g shows the reproducibility of amplitude modulation at 1100~nm (I assume, from the same design as in panels e,f)? The authors should show the reproducibility of a parameter critical for one of the two proposed designs - either amplitude at 1057~nm or phase for 1500~nm.
While overall the paper is well written, I found several confusing points that need addressing and a more clear explanations:
In lines 153-155, the authors discuss the drawbacks of using column/row electrode configuration. Is there a research that illustrates that the issues mentioned by the authors are indeed critical? For example, would the time delays induced by the grid contact configuration be of any importance taking into account the relatively low switching speed of the device?
I did not follow the explanation in lines 237-239. Why a more volatile and fast amorphization process ends up being more controllable?
Could the authors specify what they mean by "large-sized" metasurface pixels in lines 315-317?
In the last section of the paper, the authors demonstrate the spectral reconstruction performance in two configuration with static and dynamic operation of the device. Here, the flow of the discussion makes it hard to follow how the original metasurface pixel design was chosen. In particular, the figure captions are too concise and miss some relevant information. I believe that the pixel design is fixed by what is presented in Fig. 4c for static configuration (the vertical axis - radius - is, I assume, the radius of the holes in Sb2Se3 metasurface, but is not specified anywhere) and in Fig. 5a right (here, the vertical axis is index, which is even more confusing). Was these designs optimized somehow or just chosen for uniform coverage of the region of interest with spectral features of the metasurface? Why the particular metasurfaces in Fig. 5a were chosen to realize the tunable spectral reconstruction? Finally, does the data in Fig. 5b mean that using an 18-unit static metasurface

arrangement shows exactly the same performance as a tunable arrangement of the same size, and the gain only happens when the number of units is increased?

(Remarks on code availability)

Reviewer #2

(Remarks to the Author)

The manuscript addresses one of the key challenges that currently stand in the way of taking phase-change metasurfaces from the laboratory to real-world practical application, namely how to carry out selective (pixelated) in-situ switching. While several previous works have shown the use of integrated microheaters, both metal and ITO, to successfully switch phase-change metasurfaces, very few, if any, to date have shown the full implementation of pixelated metal heaters - though it is noted that the company Bodle Technologies Ltd developed and patented a pixelated heater array for phase-change display purposes (see e.g. patent application US 2025 0155734 A1). The current work is therefore novel and brings significant value to the community. However, the technical achievements presented in the paper do have some significant limitations, as recognised in most cases by the authors, including a small number of pixels (36), a relatively low fill factor (phase-change cell size/total pixel area) and relatively slow switching speeds which, in the best case, are in the 10s kHz range and limited by the time required for crystallization and the maximum chip duty cycle. Nonetheless, in spite of such limitations and in light of the importance of the manuscript's subject matter (in-situ pixelated switching of pcm metasurfaces) and the quality of results presented, the paper would, in my opinion, be suitable for publication in Nature Communications subject to the following amendments, clarifications and modifications.

Introduction section
1. In the introduction the references jump from #30 to #48, and ref 48 does not contain examples of the electrical switching discussed in the manuscript text (it features optical switching of integrated photonic devices). The authors should check that reference numbering is correct throughout the manuscript and reference in a systematic way.

In the section "Design and characterization of electrically-driven Sb2Se3 metasurfaces"

2. In the discussion on direct vs indirect heating, it seems that by 'direct' heating the authors mean Joule heating by electrical current injection into the phase-change material; if this is the case, the authors should specify this (i.e. specify the link between direct heating and current injection).

3. When discussing the use of microheaters to switch phase-change metasurfaces, the authors should cite all the relevant papers, not just ref #49 as currently (see e.g., Y. Wang et al, Nature Nanotechnology, vol. 16, no. 6, pp. 667–672, 6 2021; C. C. Popescu et al, Advanced Materials, 2400627 2024; O. Hemmatyar et al, Conference on Lasers and Electro-Optics (2021), paper FTh4K.1, 5 2021.)

4. In Fig. 1, the pulse durations used for annealing and for melt-quench are the wrong way round. Also, why is the top surface of the phase-change layer in the stack shown as crystalline, with the rest of the layer as amorphous? I doubt that such a configuration is achieved during switching, so to show it here is likely to be confusing to the reader.

5. Why was the choice made to limit to 6x6 pixel array? Was the size just limited by their fabrication facilities, or is it a more fundamental limit due to the array design? It would be helpful to include further discussion on the limitations of their array design in terms of scalability (how many pixels might be possible, how small the individual pixels might be made, how the fill factor could be improved).

6. The fill factor of the array is relatively low, at 50%. This, I presume, means that half the light incident on the array is not affected/modulated by the metasurface. Does this therefore mean that modulation efficiencies of 60% quoted by the authors are really only 30%, since half the light incident on the array is not affected by the metasurface?

7. 500 switching cycles are shown in Fig. 2g, but what happens after 500 cycles, for example did the array stop working, or did the authors just stop testing? What is the expected maximum number of cycles that might be achieved, and what limits this maximum number? Note also that the number 500 on the axis of this figure has been cropped and so reads as 50.

8. Were the results shown in Fig 2 obtained by simultaneously switching the whole array, i.e., all 36 pixels, or are they from a single or subset of pixels? This information should be clear to the reader.

9. On page 6, last paragraph, the authors say ….the low phase-change threshold of the negative structure array ….helps to deliver a low driving voltage threshold. It is not clear what is meant by a low phase-change threshold – if this is the amorphization or crystallization temperature, then surely this just depends on the material and not on whether it is a negative or positive pattern (holes or cylinders). Some additional explanation/clarification is needed here.

10. In the same paragraph, the authors talk about working in the 'weakly crystallized range' – further clarification of the

meaning of this is needed. For example, do they mean crystallizing up to something like the 34% crystallization fraction mentioned earlier in the paragraph, but no further? And how does restricting the crystallization fraction (if indeed that is what is done here) ensure that random crystallization does not occur?

11. Bearing in mind the comment above, are the results shown as for the ‘crystalline’ state in Fig. 2 for a fully, or weakly, crystallized state? This information should be made clear to the reader.

12. Ref #45 discusses Sb2S3 materials, not the Sb2Se3 material used by the authors. Are the findings for the behaviour of Sb2S3 in ref. #45 transferrable to Sb2Se3? If so, the authors should say so and state why.

13. On page 7 and elsewhere the authors talk about ‘on-chip photonic devices” – here I think they are referring to integrated photonic devices. Indeed, since their own device is a form of on-chip photonic device, the authors should be clearer as to what type of device they are referring to wen they say ‘on-chip’.

14. Towards the bottom of page 8 the authors extract a crystallization time of sub-8.77 microseconds from the results shown in Fig. 3a. However, as the authors state, during the heating/switching process both volatile and non-volatile changes are occurring, and one effect can mask the other. The untangling of these two processes to extract a specific crystallization time does not seem straightforward, and the explanation given by the authors (relating to the plateau region) does not seem very convincing – certainly it would not seem reasonable to extract such a precise time of 8.77 microseconds using their arguments. The authors should strengthen their argument here if they do indeed believe the figure of 8.77 microseconds can be extracted from the result shown, or else they should be less precise and, for example, simply state that the actual crystallization time is likely to be significantly smaller than 50 microseconds.

15. On page 9 the authors compare the speed of their device to conventional lcd modulator devices. However, digital micromirror devices are also capable of modulation, and these have speeds into the 10s of kHz currently. Some mention of these (as competitor type devices) should be made.

16. Also on page 9, the reason for limited crystallization levels is given as local hot spots restricting crystallization. Do the authors have any quantitative evidence for this, for example FE thermal simulations of the heating of their structure should be relatively straightforward to obtain to validate the hot spot mechanism.

Section on phase-change metasurface array for spatial spectral reconstruction

17. In Fig 4c, the authors should make clear if the transmission matrix shown is experimental or simulation.

18. Why is the duty cycle limited to 50%? Is it because the electrode leads are damaged for higher duty cycles, and if this is the case, would the duty cycle not depend on the voltage applied (and current flowing)?

19. On page 12 the authors mention ‘second order phase change control’ – what do they mean here, is it just partial/weak crystallization again, or something different? Please clarify.
Conclusions

20. The authors claim their work demonstrates for the first time an electrically controlled two-dimensional phase-change metasurface array. However, other researchers are also currently developing electrically controlled 2d metasurface arrays - for example Hu et al at MIT recently (September 2025) demonstrated a pixelated Sb2S3 array device for mid-infrared spatial light modulation at the ECPOS 2025 conference in Marseille. So, perhaps the claim of being ‘first’ is open to discussion and should be downplayed?

(Remarks on code availability)
I accessed the code but did not run it due to limited time.

Version 1:

Reviewer comments:

Reviewer #1

(Remarks to the Author)
The authors addressed all the comments convincingly. I recommend the paper for publication.

(Remarks on code availability)

Reviewer #2

(Remarks to the Author)
The authors have addressed all of the points I rasied in the first review stage in a comprehensive and satisfactory fashion.
I am happy therefore to support the publication of the revised manuscript in Nature Communications.

(Remarks on code availability)
N/A

Open Access This Peer Review File is licensed under a Creative Commons Attribution 4.0 International License, which permits use, sharing, adaptation, distribution and reproduction in any medium or format, as long as you give appropriate credit to the original author(s) and the source, provide a link to the Creative Commons license, and indicate if changes were made.
In cases where reviewers are anonymous, credit should be given to 'Anonymous Referee' and the source.
The images or other third party material in this Peer Review File are included in the article's Creative Commons license, unless indicated otherwise in a credit line to the material. If material is not included in the article's Creative Commons license and your intended use is not permitted by statutory regulation or exceeds the permitted use, you will need to obtain permission directly from the copyright holder.
To view a copy of this license, visit https://creativecommons.org/licenses/by/4.0/

Reviewer#1,:

Overall comment:

In their work, Zeng et al. demonstrate for the first time electrically driven, which they apply for amplitude and phase modulation of light in the near-infrared range as well as its spectral reconstruction. The work is a sound addition to the rapidly developing area of phase change tunable photonic structures. From that standpoint, I find the results appealing to a broad audience and meeting the high standards of Nature Communications. Still, there are a few critical points that in my opinion need to be addressed before the paper is accepted for publication.

Author response:

We are grateful to the reviewer for the thoughtful evaluation of our manuscript and for the encouraging remarks regarding the significance and quality of our work. We equally appreciate the critical points raised, which have helped us strengthen the paper. A detailed response to each comment is provided in this letter. We have thoroughly revised the manuscript to reflect all necessary clarifications and improvements as suggested.

Reviewer#1, Comment # 1:

The results need to be better positioned against the latest research on electrically controlled PCM optical devices. In particular, there is the recent paper by Popescu et al. Advanced Materials (2024)https://doi.org/10.1002/adma.202400627, which reports on a device with similar functionality based on GSST that also operates in the near-infrared range. In this context, I believe that the claim "this work establishes a novel paradigm for dynamic optical devices…" should be suspended from the abstract as not being true. Furthermore, while some of perks of using lower loss Sb2Se3 are rightfully mentioned by the authors, there are also additional advantages that can be highlighted (from what I can tell, lower switching voltages/energy consumption and no phase separation issues due to binary nature of the compound) and some potential tradeoffs that needs to be discussed in more detail.

Author response:

We sincerely thank the reviewer for this constructive suggestion and for bringing the highly relevant work by Popescu et al. to our attention. We agree that a clearer positioning of our results within the latest advancements in electrically controlled phase-change metasurfaces is essential. In response, we have revised the manuscript accordingly. Specifically, the claim noted by the reviewer, "*this work establishes a novel paradigm for dynamic optical devices…*", has been removed from the abstract to ensure precision. Furthermore, the introduction has been expanded to provide a more thorough comparison with recent works, including the cited study, acknowledging the shared design paradigm of integrating micro-heaters with phase-change metasurfaces for electrical tuning.

Beyond this common framework, our work introduces distinct advances. We have elaborated on the benefits of using binary Sb_2Se_3, which, compared to materials like GSST, offers lower optical loss in the near-infrared and avoids potential phase separation issues. Our experimental results confirm that the designed micro-heater structure enables switching with relatively low voltages (below 10 V for crystallization and below 20 V for amorphization), highlighting advantages in energy consumption. The core innovation lies in scaling the device architecture from a single

element to a fully addressable two-dimensional array. We demonstrate a 6×6 pixelated Sb_2Se_3-based metasurface with independent electrical control, moving beyond single-pixel modulation. Finally, we utilize this array to experimentally demonstrate a functional application in computational spectral reconstruction, validating its potential for integrated photonic systems. We believe these aspects solidify our contribution in advancing scalable, low-loss phase-change metasurface arrays, and we thank the reviewer for helping us articulate this more clearly. All corresponding revisions have been made in the manuscript.

Corrections:---

(line 38, Abstract): Modified

This work establishes ~~a novel~~ an effective paradigm for dynamic optical devices that synergistically combine electrical reconfigurability with phase-change metasurface properties.

(line 66-75, Introduction): Modified

To overcome this, indirect electrical control using integrated micro-heaters ~~emerged, successfully demonstrating phase switching in materials like GST and GSST with larger pixel sizes reaching 0.4×0.4 mm² [49].~~ was developed. This approach has been successfully applied to various phase-change materials, including GST [41-42] and GSST [43-44], and validated for both transmissive [43] and reflective spatial light modulation [41-42,44]. Very recently, this indirect electrical control approach has been further extended to two-dimensional, addressable phase-change metasurface arrays based on GSST, providing a promising new route for pixelated spatial light modulation [45]. Despite these advances in electrical control methodologies, a critical gap remains.~~: while low-loss PCMs such as Sb_2Se_3 and Sb_2S_3 are highly promising for efficient optical modulation [40-42], their effective integration into electrically driven phase-change metasurfaces has not yet been demonstrated.~~ Low-loss PCMs such as Sb_2S_3 and Sb_2Se_3 have attracted significant attention due to their uniquely low optical loss in both amorphous and crystalline states, along with the inherent advantage of their binary composition, which suppresses phase separation. Although early studies indicated that their crystallization could suffer from spatial randomness, posing a challenge for large-area, uniform modulation, recent work on spatially confined, nucleation-guided crystallization has demonstrated more uniform phase switching in Sb_2S_3, renewing interest in these materials [46]. Nevertheless, their integration into electrically driven phase-change metasurfaces has thus far been relatively unexplored, and a practical, scalable platform for dynamically controlling Sb_2S_3 or Sb_2Se_3 metasurfaces via integrated electronics has not yet been demonstrated. Although Sb_2Se_3 phase switching via PIN heaters in integrated photonics and recently in metasurfaces as a tunable dielectric environment has been reported ~~[51-52]~~[47-48], these approaches introduce high-resistance microheaters, micrometer-scale pixel constraints, and necessitate complex optical designs to accommodate the high-index Si substrate, significantly limiting Sb_2Se_3/Sb_2S_3 metasurface scope and performance.

Reviewer#1, Comment # 2:

The authors take additional effort to highlight the importance of decoupling of the heater electrodes with the "optical" part of the device (line 83; line 136). While I understand that the direct contact of titanium with the Sb2Se3 metasurface might deteriorate the optical performance, why did the authors not consider using gold-only electrodes? Does the use of titanium allow to optimize the spatial distribution of the power loss? This needs to be explained more thoroughly.

Author response:

We sincerely thank the reviewer for this valuable question and the opportunity to clarify the rationale behind our electrode design. The choice to employ a titanium heating electrode beneath the gold reflector in our hybrid metasurface structure was based on two primary considerations: thermal stability and the need for localized power dissipation.

Firstly, regarding thermal stability, titanium has a melting point (~1668 °C) significantly higher than that of gold (~1064 °C). The re-amorphization ("reset") process for Sb_2Se_3 requires the heating electrode to momentarily deliver temperatures exceeding 600 °C (Advanced Functional Materials, 2023, 33(42): 2304601). The superior thermal stability of titanium compared to gold ensures greater reliability during repeated high-temperature phase-change cycles.

Secondly, and more critically, this design aims to spatially concentrate the electrical power dissipation precisely within the electrode area. As the reviewer rightly inferred, we leverage the significant difference in resistivity between titanium and gold. In our layout, the narrow, micron-scale connecting wires are made of low-resistivity gold, while the broader, tens-of-microns square heating pad is made of higher-resistivity titanium. This design ensures that the majority of the Joule heating occurs within the titanium pad where it is needed to heat the phase-change metasurface above it. A pure gold design, where both the wires and the pad share the same low resistivity, would lead to substantial and wasteful heating along the much longer and narrower wire traces, reducing heating efficiency at the target site.

To substantiate this point, we have added supporting simulations in the revised Supporting Information. These simulations directly compare the temperature distribution for our Ti/Au design versus a pure Au electrode design under identical driving conditions. The results clearly demonstrate that our Ti/Au design effectively confines the heat generation to the titanium pad region during both crystallization and amorphization pulses, thereby minimizing wasted power and validating our design approach. We have revised the main text to include a reference to this explanation.

Corrections:--

(line 127, Main article): Added

The upper hybrid phase-change metasurface consists of a gold reflective layer, an Al_2O_3 dielectric layer, and an Sb_2Se_3 micropore array. Instead of utilizing the top gold optical reflector directly as a heating element, in our design, we integrated a dedicated Ti/Au micro-heater beneath it. This architectural choice was driven by two key considerations: thermal stability and the need for spatially localized power dissipation. First, the thermal stability requirement stems from the high transient temperatures needed for the phase-change cycle. Titanium, with its melting point (~1668 °C) significantly higher than that of gold (~1064 °C), offers superior robustness. This is critical for the re-amorphization ("reset") process of Sb_2Se_3, which demands that the heater briefly

exceed 600 °C [49]. The use of Ti thus ensures reliable operation over repeated switching cycles. Second, and more fundamentally, this bilayer design enables efficient spatial confinement of Joule heating. We leverage the pronounced resistivity contrast between Ti and Au: the narrow, micron-scale interconnects are made of low-resistivity Au, while the broader, tens-of-micron heating pad is made of high-resistivity Ti. This configuration ensures that the majority of the heat is generated within the Ti pad directly beneath the phase-change metasurface, where it is needed. A monolithic Au heater would suffer from significant parasitic heating along the elongated, narrow interconnect traces, drastically reducing the heating efficiency at the target pixel. Supporting finite-element simulations, provided in the Supporting Information S1 confirm this rationale. They demonstrate that our Ti/Au design effectively confines the temperature rise to the Ti pad region during both crystallization and amorphization pulses, minimizing wasted power and validating the chosen approach.

(Section S1, Supporting Information): Added

Section S1. Thermal Simulation Comparison between All-Au and Ti/Au Heater Designs

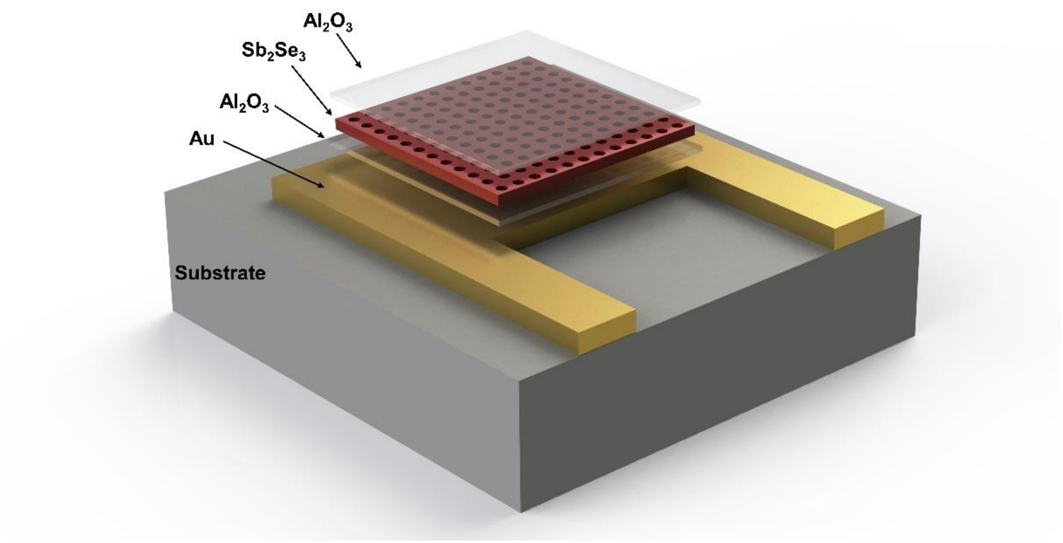

Figure S1. 3D schematic of the all-Au micro-heater integrated with the Sb_2Se_3 metasurface pixel.

In this section, we present a comparative electro-thermal analysis of the Au/Ti bilayer micro-heater employed in our work and a conventional all-Au heater. Finite-element simulations were performed using the COMSOL Multiphysics software suite to evaluate the temperature distribution within a single, representative pixel during electrical crystallization and amorphization pulses. While the simulation models an individual pixel rather than the full array, it provides sufficient insight into the fundamental heating efficiency and thermal profiles of the two designs. For a fair comparison, the pixel dimensions were kept consistent: a central rectangular heater area of 85 μm × 150 μm, with 32-μm-wide extending leads, supporting a 75 μm × 75 μm Sb_2Se_3 metasurface atop its central region (structural schematic in Figure S1).

Figure S2 displays the simulated temperature distributions under applied electrical pulses. For the

Au/Ti bilayer design (Figure S2a, b), the significant resistivity contrast between the Au leads and the Ti pad effectively confines Joule heating to the central Ti region directly beneath the metasurface. The lead regions exhibit negligible temperature rise, confirming that electrical power is localized efficiently to drive the phase change. In contrast, for the all-Au heater (Figure S2c, d), the narrow lead width relative to the broad heater pad results in substantial Joule heating along the interconnect traces rather than within the target region beneath the metasurface. As a result, a significant portion of the electrical power is dissipated wastefully in the leads. Although simulations confirm that a 2.5 V, 50 μs pulse can elevate the metasurface region to the crystallization temperature (~473.15 K), the lead temperature simultaneously approaches the melting point of gold (~1064 °C), posing a serious risk of thermal damage during operation. This issue becomes even more pronounced during the amorphization process: the simulation reveals that the lead temperature already nears the gold melting threshold before the metasurface region reaches the required amorphization temperature (~893.15 K) [1]. Consequently, the all-Au design fails to provide a reliable and efficient pathway for reversible phase switching, particularly for the amorphization operation. These findings demonstrate that the all-Au heater suffers from both prohibitive power inefficiency and fundamental limitations in achieving controlled amorphization. Therefore, the Au–Ti bilayer architecture was adopted in this work to ensure effective thermal localization, improved energy efficiency, and reliable phase-change switching.

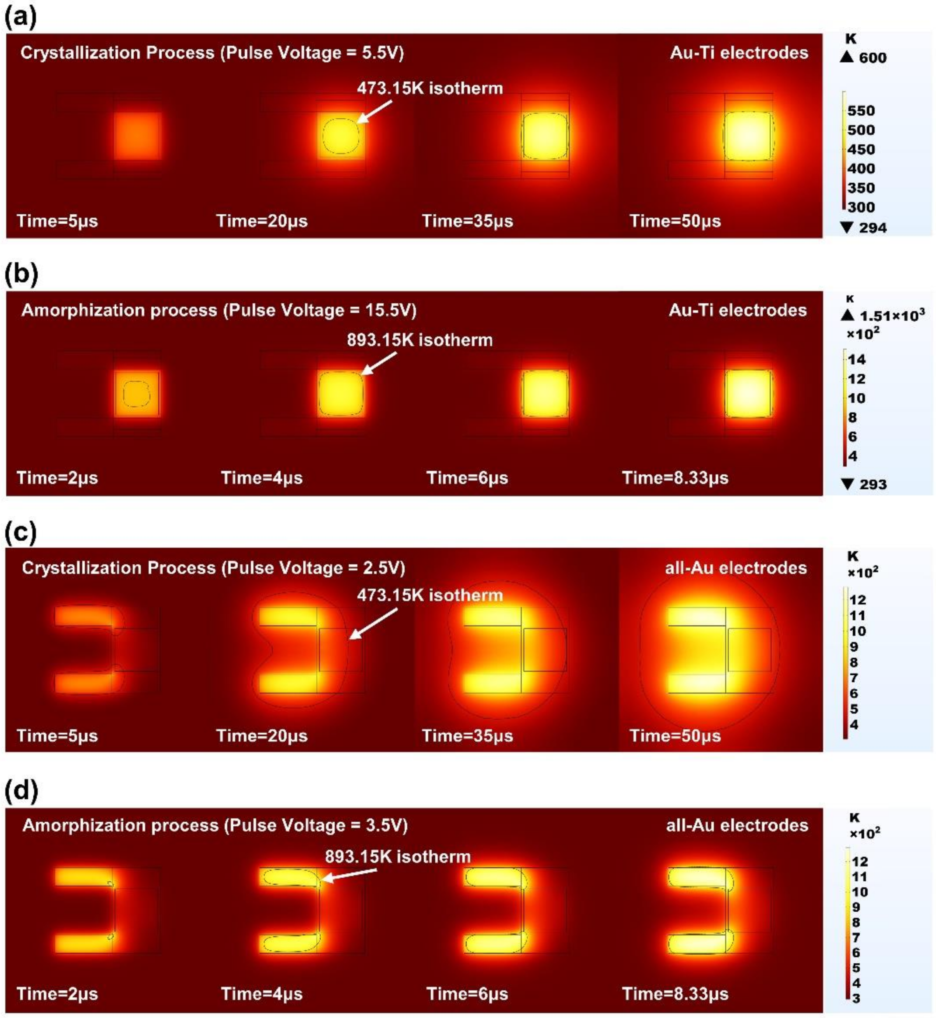

Figure S2. Comparative thermal simulations of the Ti/Au and all-Au micro-heaters. Temperature distributions during (a) crystallization and (b) amorphization for the Ti/Au design, and during (c) crystallization and (d) amorphization for the all-Au design.

It is also important to note a key limitation of our current Au/Ti design: significant thermal non-uniformity, or a central "hot spot," is observed within the Ti pad (Figure S2a, b). This inhomogeneity is particularly pronounced during the high-power, short-duration amorphization pulse. For instance, under a 15.5 V, 8.33 μs amorphization pulse, while the entire Ti pad exceeds the required amorphization temperature of Sb_2Se_3 (~893.15 K), the central region reaches nearly 1500 K. This thermal gradient is a critical factor limiting the uniformity and controllability of the phase transition in the Sb_2Se_3 metasurface. Prior studies have proposed geometric optimization of heater shapes to improve thermal uniformity [2], which represents a promising direction for future refinement of our platform.

Reviewer#1, Comment # 3:

I found Figure 2 quite confusing, as it contains data that covers 3 different spectral ranges, making it quite difficult to compare and analyze. First, I did not immediately find the part where the authors explicitly address the much lower off-resonance reflectivity amplitude of the re-amorphized device (Fig. 2b, experiment); I believe that the only place that mentions it is line 216, which should be expanded further to explain the difference. Second, I was puzzled why panel g shows the reproducibility of amplitude modulation at 1100~nm (I assume, from the same design as in panels e,f)? The authors should show the reproducibility of a parameter critical for one of the two proposed designs - either amplitude at 1057~nm or phase for 1500~nm.

Author response:

We thank the reviewer for pointing out the lack of clarity in the original Figure 2 and for the specific suggestions to improve it. We sincerely apologize for any confusion caused by the initial presentation.

In response to this comment, we have updated the experimental data in Figure 2 based on a re-designed Sb_2Se_3 metasurface unit cell that supports an under-coupled resonance in the near-infrared band. By controlling the phase transition of the material, we shift the resonance frequency, thereby enabling effective amplitude modulation within the same near-infrared range where multi-level phase modulation is also demonstrated. This consolidated presentation in a unified spectral window facilitates a clearer comparison and interpretation of both modulation mechanisms.

Regarding the lower off-resonance reflectivity of the re-amorphized state (formerly Figure 2b), we have expanded the discussion in the revised manuscript. We attribute this difference primarily to incomplete re-amorphization, where residual crystalline nuclei within the Sb_2Se_3 film lead to increased random scattering, which can be equivalently viewed as an effective increase in the material's intrinsic absorption. This phenomenon is supported by our prior work on Sb_2Se_3 (Laser & Photonics Reviews, 2024, 2400293), where we demonstrated that the discrepancy between simulated and experimental reflection spectra after cycling could be effectively modeled by introducing an additional extinction coefficient in the material's dielectric constant. We have now incorporated this explanation and the relevant citation into the revised main text to provide a more thorough justification.

Furthermore, following the reviewer’s suggestion, we have updated the cycling endurance test results in the revised figure. The test was performed on the same metasurface pixel used for the amplitude modulation experiments, and the cycling behavior is characterized by monitoring the reflectance change at the same wavelength of 1577 nm, ensuring a consistent metric for assessing phase-switching reversibility.

We believe the updated figure and the expanded explanations have significantly improved the clarity and logical flow of this section, and we thank the reviewer again for this constructive feedback.

Corrections:--

(Figure 2, Main article): Modified

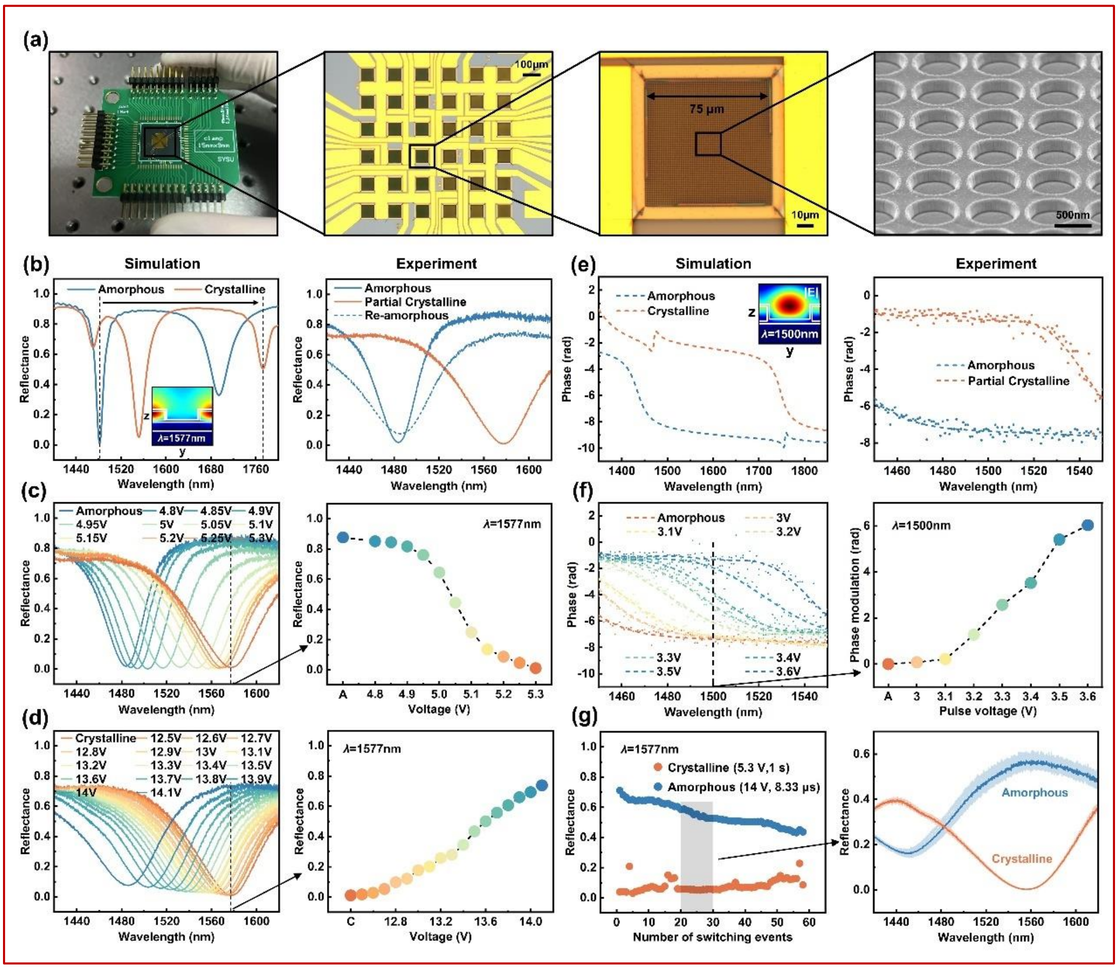

Figure 2. Single-pixel ~~D~~dynamic optical modulation enabled by electrically-driven phase transition of Sb_2Se_3 metasurfaces. a, Microscope image of the fabricated electrically-driven Sb_2Se_3 phase-change metasurface array and scanning electron microscope (SEM) image of the micropore array within the metasurface. b, ~~Simulated and experimentally measured reflectance spectrum of the under-coupled resonant metasurface~~ Simulated (amorphous and fully crystalline) and experimentally measured (amorphous and electrically switched, partially crystallized) reflectance spectra of the under-coupled resonant metasurface designed based on the Sb_2Se_3 micropore array ~~at amorphous and crystalline states~~. The inset shows the simulated electric field distribution in the amorphous micropore structure supporting the under-coupled resonance mode. c, Experimentally measured reflectance spectrum of the Sb_2Se_3 metasurface under crystallization pulses with different voltages, demonstrating the multi-stage amplitude modulation effect at ~~1057 nm~~ 1577 nm due to multi-stage crystallization. d, Experimentally measured reflectance spectrum of the Sb_2Se_3 metasurface under amorphization pulses with different voltages, demonstrating the multi-stage amplitude modulation effect at ~~1057 nm~~ 1577 nm based on multi-stage amorphization. e, ~~Simulated and experimentally measured reflective phase spectrum of the over-coupled resonant metasurface~~ Simulated (amorphous and fully crystalline) and experimentally measured (amorphous and electrically switched, partially crystallized) reflective phase spectra of the over-coupled resonant metasurface designed based on the Sb_2Se_3 micropore array~~ at amorphous and crystalline~~

states. The inset shows the simulated electric field distribution in the amorphous micropore structure when the over-coupled resonance mode is excited. f, Experimentally measured reflective phase spectrum of the Sb_2Se_3 micropore array under crystallization pulses with different voltages, with the reflective phase modulation at 1500 nm during the multi-stage crystallization process highlighted. g, Experimentally measured changes in the reflectance during 500 approximately 60 phase-transition cycles, with the varying spectrum during 30 10 selected phase-change cycles shown in detail. Crystallization was driven by a 5.3 V, 1 s pulse, and amorphization was driven by a 14 V, 8.33 μs pulse.

(line 213-221, Main article): Modified

A single 3.25 V pulse lasting 2 s 5.3 V pulse lasting 1 s can achieve approximately 34% 40 % crystallization (please refer to Supporting Information S2 S4 for crystallization level estimation); whereas a single 8.6 V 14.1 V pulse lasting 8.33 μs can achieve nearly complete amorphization, restoring the reflectance spectrum to a state close to the initial amorphous state. A noticeable decrease in off-resonance reflectivity can be observed in the re-amorphized state compared with the initial amorphous state in the experimental spectra of Figure 2b. In this work, this reduction is primarily attributed to incomplete re-amorphization, in which residual crystalline nuclei within the Sb_2Se_3 film enhance random scattering. This scattering effect can be equivalently described as an effective increase in the material's intrinsic absorption. Such spectral deviation is consistent with our earlier study on Sb_2Se_3 metasurfaces, where the discrepancy between simulated and experimental reflection spectra was accurately reproduced by introducing an additional extinction coefficient into the material's dielectric constant [54]. Clearly, by increasing the voltage or duration of the crystallization pulse, a higher level of crystallization can be achieved. However, during the crystallization process of the one-dimensional chain-like crystalline material such as Sb_2Se_3, random lattice growth is inevitable, leading to spatially non-uniform refractive index distribution in the highly crystallized state, which affects the optical response of the metasurface [45].

(line 231-234, Main article): Modified

As shown in Figures 2c and 2d, the center wavelength of the resonance mode gradually changes with the varying voltage of the crystallization or amorphization pulse, manifesting as multi-stage controllable amplitude modulation at the target wavelength (achieving 7-stage 12-stage amplitude modulation during crystallization and 14-stage 18-stage amplitude modulation during amorphization).

(Section S3, Supporting Information): Modified

Section S2 S4. Design and simulation of the phase-change meta-atom

Figure S2S6a and S2S6b illustrate the simulated reflection and phase spectra of the undercoupled phase-change metasurface based on Sb_2Se_3 micropore arrays during the phase transition process. In the amorphous state, the Sb_2Se_3 micropore structure exhibits significant resonant absorption near 1050 nm 1480 nm, accompanied by a phase jump, indicating that the current resonant mode is in an undercoupled state. As the crystallization ratio increases, the resonant mode experiences significant redshift in frequency, and the reflectance at the resonance center wavelength remains close to zero, indicating that the resonant mode maintains an undercoupled state near critical coupling throughout the phase transition process. This phenomenon is attributed to the strong confinement of the resonant

mode's electric field within the gaps of the adjacent micropore structures, as shown in Figure ~~S2~~ S4c and ~~S2~~S4d. Due to the intrinsic absorption characteristics of the underlying gold reflective layer in the near-infrared band, the internal dissipation rate γ_d remains exceeds radiation coupling rate γ_s. The frequency shift of the undercoupled resonant mode introduces amplitude modulation across the scanned wavelength range. However, it is important to note that, due to the significant optical contrast of the phase-change material, adjacent higher-order resonant modes also sweep through the operating wavelength during the phase transition, resulting in the nonlinear amplitude modulation shown in Figure ~~S2~~S4e. To address this issue, during the experimental process, the phase-change material was intentionally maintained within a weakly crystallized range by controlling the crystallization pulse voltage to avoid the influence of adjacent resonant modes. Moreover, prior studies have shown that a lower degree of crystallization also helps to improve the durability of the phase-change material[2]. By comparing the center wavelengths of the resonant modes after electrically driven crystallization of the metasurface, it can be found that, as shown in Figure ~~S2~~S4f, the crystallization ratio m reaches approximately ~~0.34~~ 0.4.

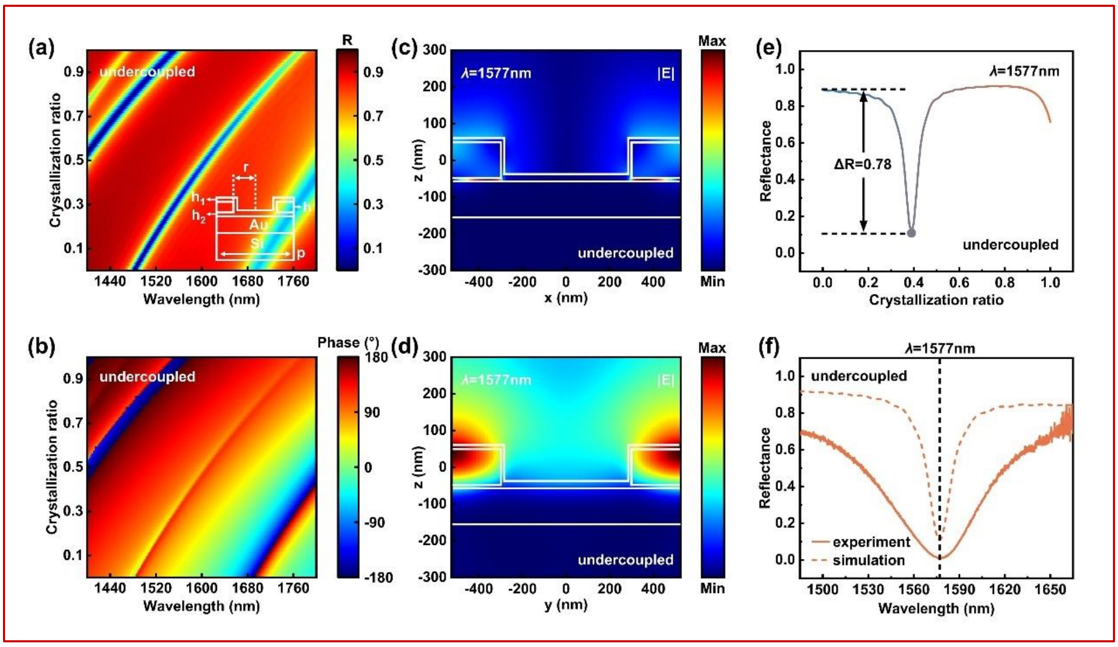

Figure ~~S2~~S6. Simulated optical response of the undercoupled Sb_2Se_3 metasurface. a, Simulated reflection spectra of the undercoupled Sb_2Se_3 metasurface at different crystallization ratios. The inset illustrates the geometric parameters of the meta-atom. Here, ~~$r = 245$ nm~~ $r = 300$ nm denotes the diameter of the micropore, ~~$h = 35$ nm~~ $h = 100$ nm is the height of the micropore, $h_1 = 20$ nm is the thickness of the Al_2O_3 protective layer on top of the micropore, $h_2 = 5$ nm is the thickness of the Al_2O_3 isolation layer between the phase-change micropore and the gold reflective layer, and ~~$p = 800$ nm~~ $p = 1050$ nm is the period of the micropore array. b, Simulated reflection phase spectra of the undercoupled metasurface as a function of crystallization ratio. c, Distribution of the electric field intensity of the resonance mode within the x-z cross-section of the meta-atom at the resonant center wavelength. d, Distribution of the electric field intensity of the resonance mode within the y-z cross-section of the meta-atom. e, Simulated amplitude modulation at the wavelength of ~~1057 nm~~ 1577 nm as a function of crystallization ratio. f, Comparison between the simulated reflection spectrum of the metasurface at ~~$m = 0.34$~~ $m = 0.4$ and the experimentally measured reflection spectrum under weakly crystallized conditions.

Reviewer#1, Comment # 4:

While overall the paper is well written, I found several confusing points that need addressing and a more clear explanations:

Author response:

We sincerely thank the reviewer for the overall positive assessment of our manuscript and for highlighting specific points that required clarification. We appreciate the careful reading and the constructive feedback, which has helped us improve the paper's clarity.

We have thoroughly reviewed the manuscript in response to your general comment and have revised it to address any potentially confusing explanations. Specifically, we have added more detailed descriptions and clarifications at key points to ensure that the methodology, results, and discussions are presented as clearly and accessibly as possible. We believe these revisions have significantly enhanced the readability and completeness of the work.

Reviewer#1, Comment # 5:

In lines 153-155, the authors discuss the drawbacks of using column/row electrode configuration. Is there a research that illustrates that the issues mentioned by the authors are indeed critical? For example, would the time delays induced by the grid contact configuration be of any importance taking into account the relatively low switching speed of the device?

Author response:

We sincerely thank the reviewer for raising this important point regarding our discussion of row-column addressing schemes, and we agree that our initial statement regarding its drawbacks (lines 153-155) was not sufficiently nuanced. We apologize for any lack of clarity and have revised the manuscript to provide a more balanced and substantiated discussion.

In the revised manuscript, we have expanded the discussion by referencing prior work on electrically addressed vanadium oxide pixel arrays (Nature Nanotechnology, 2024, 19(11): 1635-1643) to more rigorously evaluate the feasibility of a row-column electrode configuration for phase-change metasurface arrays. As illustrated in Figure R1, we designed a conceptual stack employing a Ti/Au scheme analogous to our current work: Ti as the heating pad and Au as the interconnect lines. This row-column architecture consists of a bottom Ti electrode layer, an intermediate row/column electrode layer (insulated by a 10-nm Al_2O_3 layer between the row and column electrode layer), and a top Al_2O_3 insulation layer, over which the hybrid Sb_2Se_3 metasurface is placed.

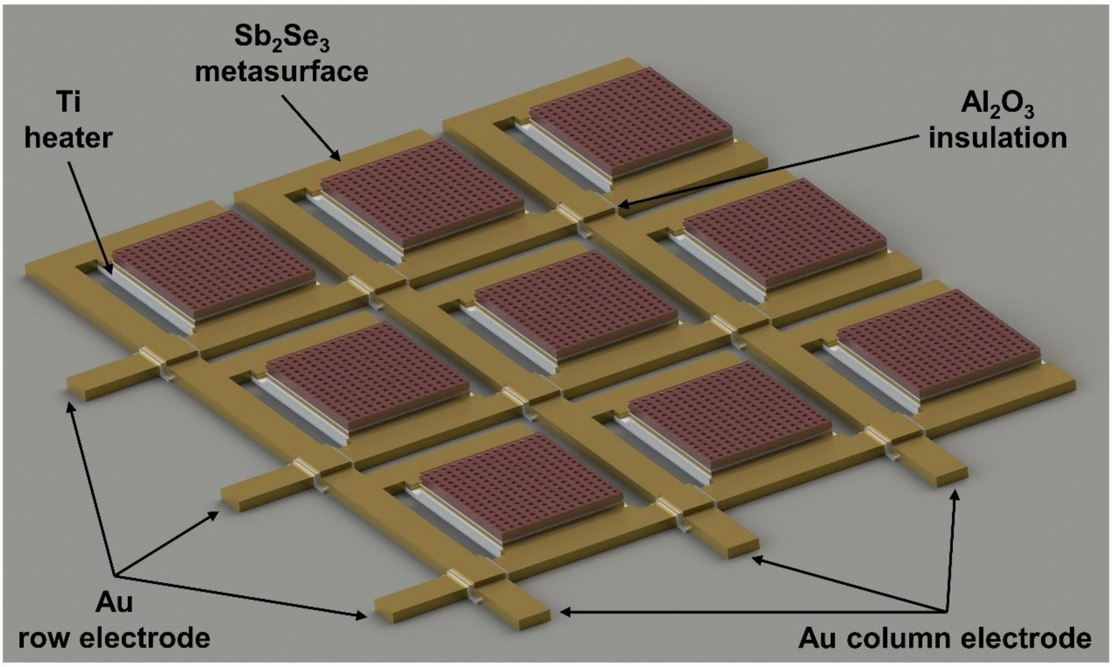

Figure R1. Schematic of the stacked architecture: a row–column addressed micro-heater integrated with a Sb_2Se_3 metasurface array.

To assess the feasibility of the proposed vertically stacked, row-column addressing scheme for electrically driving Sb_2Se_3 metasurface arrays, we performed numerical electro-thermal simulations using COMSOL Multiphysics. Although a modest 3×3 array was modeled for computational

efficiency, the architecture inherently supports straightforward scaling to larger formats. The simulation investigated both a central and a corner pixel to account for potential positional variations in thermal response.

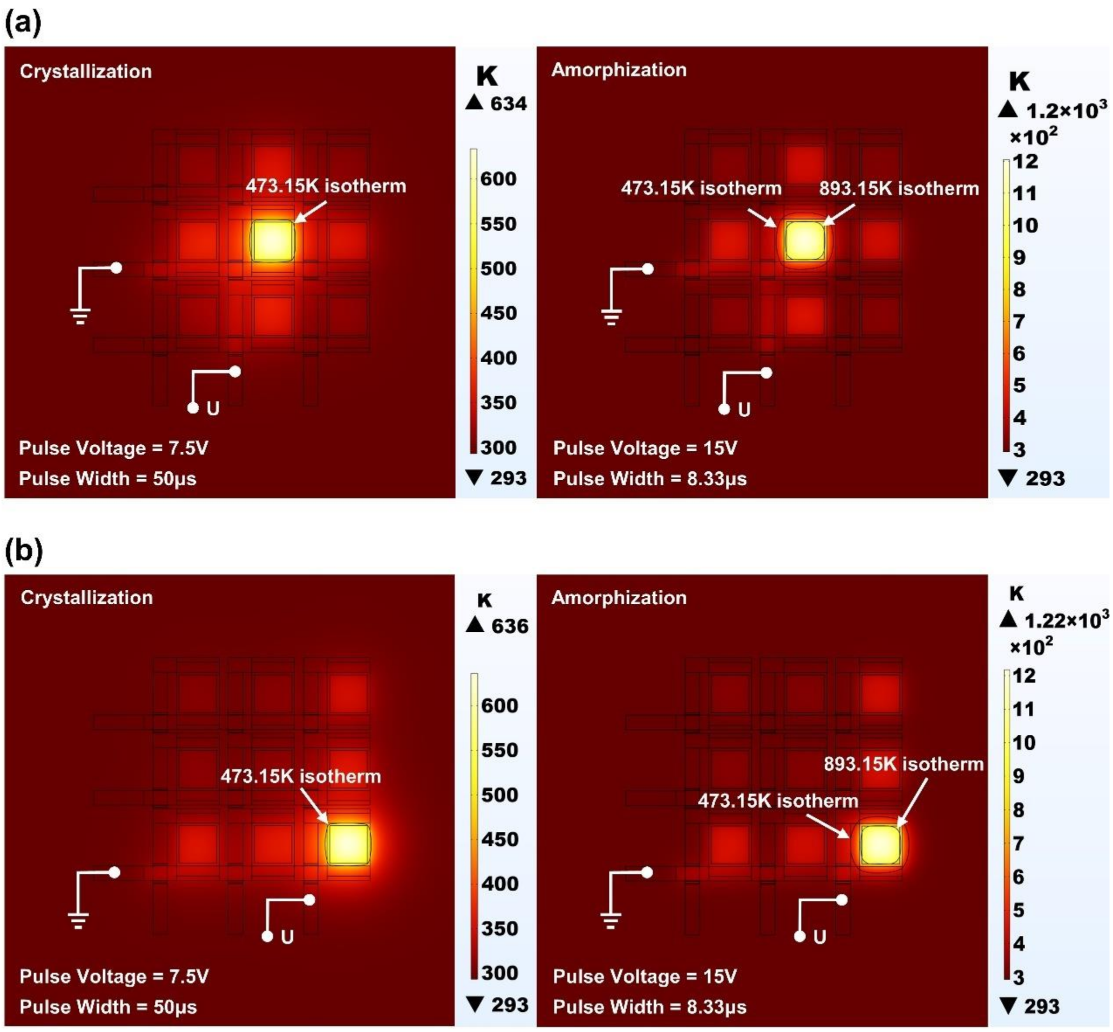

Figure R2. Independent thermal addressing of pixels in a 3×3 row-column array. Simulated temperature profiles when applying (a) crystallization (7.5 V, 50 µs) and amorphization (15 V, 8.33 µs) pulses to the center pixel, and (b) the same pulses applied to a corner pixel.

As shown in Figure R2, when appropriate row and column lines are selected and driven with optimized voltage pulses (e.g., 7.5 V, 50 µs for crystallization; 15 V, 8.33 µs for amorphization), the temperature of the targeted pixel, whether central or corner, rises sufficiently to reach the required crystallization (~473.15 K) and amorphization (~893.15 K) thresholds, with heating highly localized to the addressed site. The temperature increase in neighboring pixels arises primarily from thermal diffusion and remains well below the crystallization point in both switching regimes. Specifically, during crystallization pulses, adjacent pixel temperatures stay significantly below 473.15 K. During the higher-power amorphization pulses, although neighboring pixels experience some heating via thermal spreading, their temperatures also remain below the crystallization threshold, thereby preserving their phase states. These results confirm that the row-column addressed heater can selectively switch individual pixels without unintentionally affecting their

neighbors, demonstrating its fundamental suitability for pixel-independent phase control.

Moreover, compared to the planar-wiring scheme used in our current experimental array, the row-column architecture decouples fill factor from array size. In our simulated design, a fill factor of ~50% was readily achieved without dedicated optimization, and further improvement is attainable through layout refinement. This addresses the critical trade-off between fill factor and scalability inherent in planar routing. Lastly, as the reviewer astutely notes, the switching delays intrinsic to a row-column grid are not a critical issue given the relatively slow switching speeds of our current devices (crystallization pulse ~50 μs, amorphization pulse ~8.3 μs).

Overall, our simulations validate the row-column addressing scheme as a promising route toward scalable, high-fill-factor, and independently controllable phase-change metasurface arrays. However, we also acknowledge that the row-column configuration involves a more complex, multi-layer fabrication process (requiring multiple lithography and alignment steps) compared to the single-layer planar wiring used in our present proof-of-concept demonstration. Therefore, while the row-column approach holds great promise for future high-density, large-scale arrays, we employed the more readily fabricated planar wiring in this initial work to successfully demonstrate the core principle of randomly accessible, electrically driven phase-change metasurfaces.

We have incorporated this more comprehensive analysis into the revised manuscript and Supporting Information to provide a fairer assessment of different addressing schemes and their trade-offs, and we thank the reviewer again for prompting this valuable clarification.

Corrections:---

(line 150-158, Main article): Modified

In recent works, similar arraying schemes have also been used for pixelated electrical control of vanadium oxide active structures [54]. ~~However, in comparison, Sb_2Se_3, GST, and other nonvolatile phase-change materials are current-driven active materials with high thermal thresholds, especially for the amorphization process. During the arraying process, the row and column electrode addressing scheme causes the driving current to follow a right-angle trajectory at the corresponding pixel, resulting in inevitable temperature non-uniformity within the pixel. Meanwhile, the addressing process of the array also introduces unavoidable time delays. Therefore, a phase-change metasurface array with random access capability for each pixel is necessary to fully exploit the high-speed response potential of phase-change materials.~~ However, the row-column addressing scheme for driving non-volatile phase-change materials such as Sb_2Se_3, Sb_2S_3, and GST remains largely unexplored. In this work, inspired by previously reported vertically stacked row–column electrode architectures, we numerically validate the feasibility of the proposed scheme for selectively driving Sb_2Se_3 metasurfaces within a small-scale array through electro-thermal simulations (Please refer to Supporting Information S2 for detailed simulation results). Combined with its inherent compatibility with high fill factors and straightforward array scalability, the row-column addressing approach presents a promising route toward realizing electrically controlled phase-change metasurface arrays. Nevertheless, considering the fabrication complexity associated with vertically integrated row–column electrode stacks, the present work ultimately adopts a more readily fabricable alternative based on planar-wired micro-heaters.

(Section S2, Supporting Information): Added

Section S2: Design and Thermal Simulation of the Row-Column Addressed Heater Array

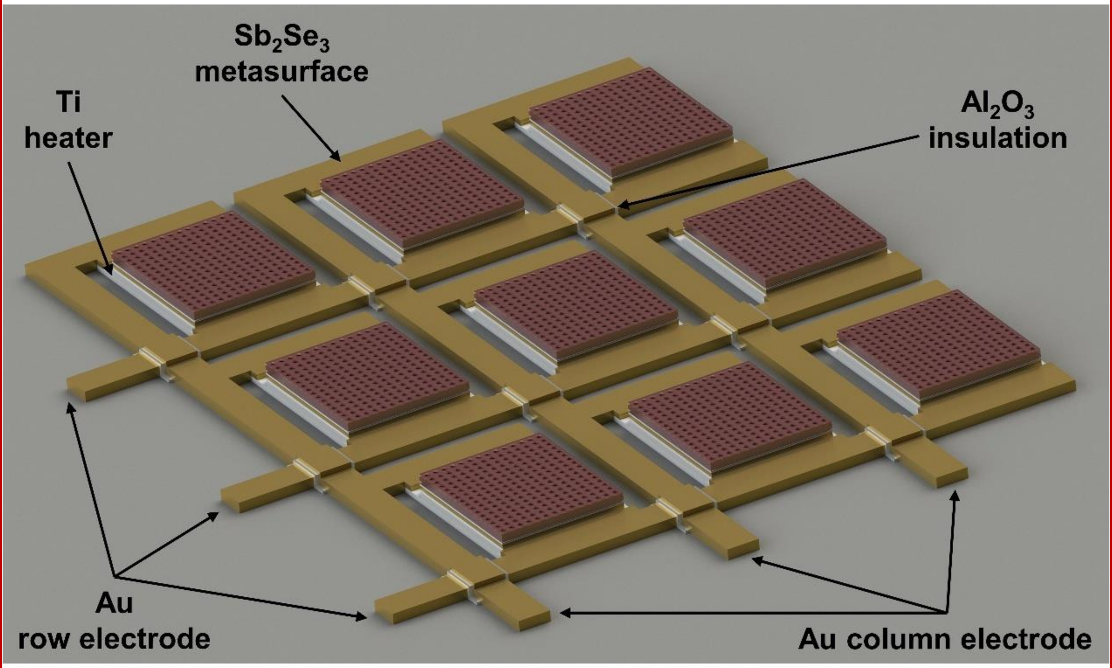

Figure S3. Schematic of the stacked architecture: a row–column addressed micro-heater integrated with a Sb_2Se_3 metasurface array.

As illustrated in Figure S3, we designed a conceptual stack employing a Ti/Au scheme analogous to our current work: Ti as the heating pad and Au as the interconnect lines. This row-column architecture consists of a bottom Ti electrode layer, an intermediate row/column electrode layer (insulated by a 10-nm Al_2O_3 layer between the row and column electrode layer), and a top Al_2O_3 insulation layer, over which the hybrid Sb_2Se_3 metasurface is placed.

To assess the feasibility of the proposed vertically stacked, row-column addressing scheme for electrically driving Sb_2Se_3 metasurface arrays, we performed numerical electro-thermal simulations using COMSOL Multiphysics. Although a modest 3×3 array was modeled for computational efficiency, the architecture inherently supports straightforward scaling to larger formats. The simulation investigated both a central and a corner pixel to account for potential positional variations in thermal response.

As shown in Figure S4, when appropriate row and column lines are selected and driven with optimized voltage pulses (e.g., 7.5 V, 50 μs for crystallization; 15 V, 8.33 μs for amorphization), the temperature of the targeted pixel, whether central or corner, rises sufficiently to reach the required crystallization (~473.15 K) and amorphization (~893.15 K) thresholds, with heating highly localized to the addressed site [1]. The temperature increase in neighboring pixels arises primarily from thermal diffusion and remains well below the crystallization point in both switching regimes. Specifically, during crystallization pulses, adjacent pixel temperatures stay significantly below 473.15 K. During the higher-power amorphization pulses, although neighboring pixels experience some heating via thermal spreading, their temperatures also remain below the crystallization threshold, thereby preserving their phase states. These results confirm that the row-column addressed heater can selectively switch individual pixels without unintentionally affecting their

neighbors, demonstrating its fundamental suitability for pixel-independent phase control.

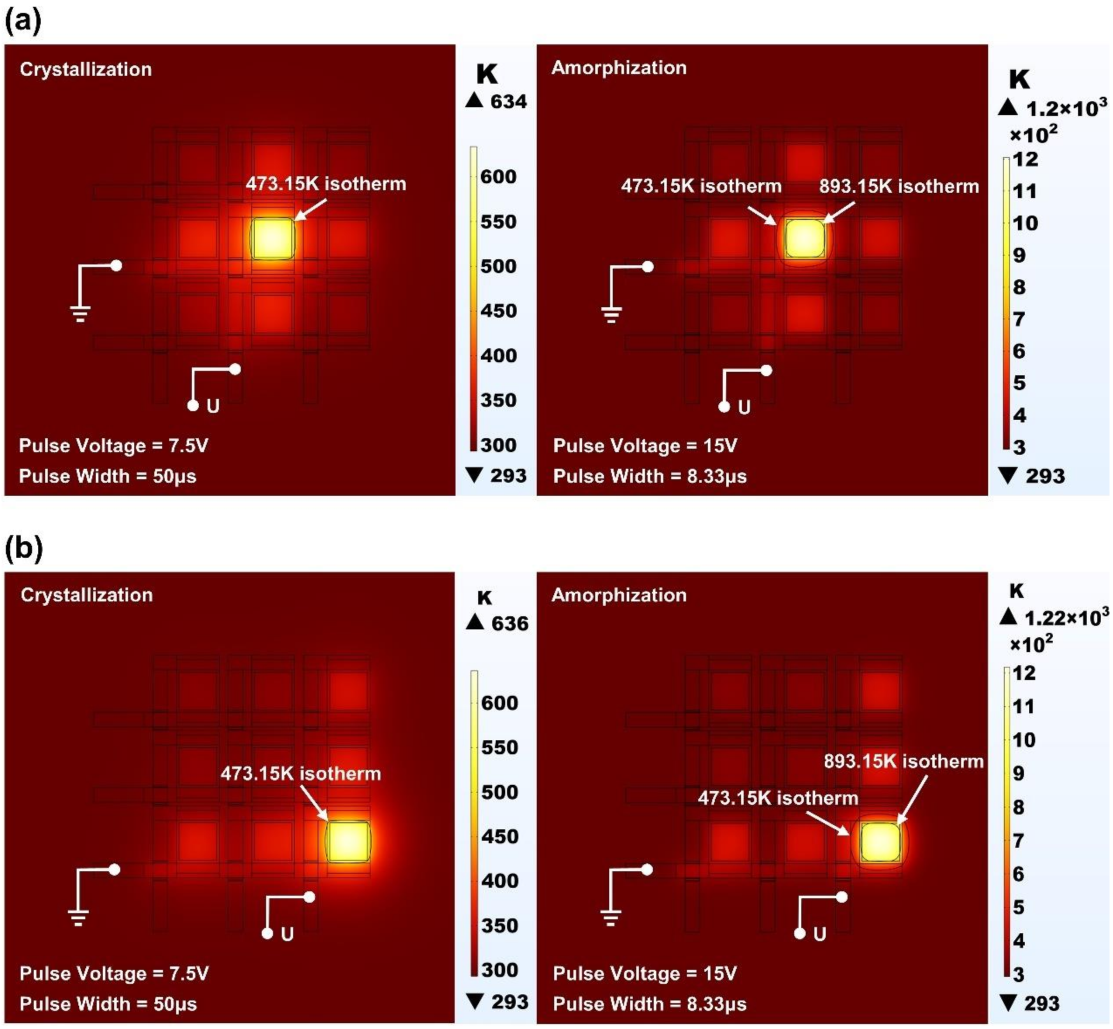

Figure S4. Independent thermal addressing of pixels in a 3×3 row-column array. Simulated temperature profiles when applying (a) crystallization (7.5 V, 50 μs) and amorphization (15 V, 8.33 μs) pulses to the center pixel, and (b) the same pulses applied to a corner pixel.

Moreover, compared to the planar-wiring scheme used in our current experimental array, the row-column architecture decouples fill factor from array size. In our simulated design, a fill factor of ~50% was readily achieved without dedicated optimization, and further improvement is attainable through layout refinement. This addresses the critical trade-off between fill factor and scalability inherent in planar routing.

Overall, our simulations validate the row-column addressing scheme as a promising route toward scalable, high-fill-factor, and independently controllable phase-change metasurface arrays. However, we also acknowledge that the row-column configuration involves a more complex, multi-layer fabrication process (requiring multiple lithography and alignment steps) compared to the single-layer planar wiring used in our present proof-of-concept demonstration. Therefore, while the row-column approach holds great promise for future high-density, large-scale arrays, we employed the more readily fabricated planar wiring in this initial work to successfully demonstrate the core

principle of randomly accessible, electrically driven phase-change metasurfaces.

Reviewer#1, Comment # 6:

I did not follow the explanation in lines 237-239. Why a more volatile and fast amorphization process ends up being more controllable?

Author response:

We sincerely thank the reviewer for this insightful question, which points to a key nuance in the discussion of intermediate phase control. We apologize for the lack of clarity and completeness in our original explanation surrounding lines 237-239.

While chalcogenide phase-change materials like Sb_2Se_3 are indeed capable of switching between multiple intermediate states, achieving precise and deterministic control over these states remains a significant challenge. As the reviewer correctly notes, the crystallization process is generally more gradual, and prior studies have demonstrated multi-level crystallization by tuning pulse amplitude or width (Nature Communications, 2024, 15(1): 2786). However, due to the nucleation-and-growth mechanism characteristic of Sb_2Se_3, the stochastic nature of nucleation introduces inherent randomness, making stable and repeatable access to specific intermediate crystalline states difficult (Annual Review of Materials Research, 2025, 55). In contrast, the amorphization ("reset") process, though more volatile, possesses a sharper thermal threshold - it requires the material to briefly exceed its melting point. This well-defined threshold, combined with the lack of a stochastic nucleation step, can provide a different pathway for multi-level control. Previous works (Advanced Functional Materials, 2023, 33(42): 2304601) have shown that by carefully increasing the amorphization pulse amplitude, multi-level re-amorphization can be achieved, sometimes even exhibiting a larger number of distinct levels than during crystallization. Our own experimental data in this work also demonstrates multi-level amorphization with a finer gradation than achieved during crystallization under our specific driving conditions.

Crucially, however, neither our results nor prior literature conclusively prove that amorphization is inherently more controllable than crystallization. The observed behavior can be highly dependent on the specific material system, device geometry, and driving scheme. The prevailing view in the field is that achieving truly precise and arbitrary intermediate states will likely require active feedback mechanisms (e.g., in-situ monitoring) to complement and guide both crystallization and amorphization pulses (Advanced Functional Materials, 2023, 33(42): 2304601).

We have revised the relevant section in the manuscript to provide this more balanced and detailed discussion. We now clarify the distinct physical mechanisms, cite supporting literature, and emphasize that the controllability is complex and scheme-dependent, removing the previous oversimplified statement. Thank you again for prompting this important clarification, which has strengthened our discussion.

Corrections:---

(line 235-239, Main article): Modified

It is worth noting that current results indicate a more easily achieved multi-level amorphization process compared to the crystallization process. ~~We attribute this behavior to the distinct temperature threshold characteristic of PCM amorphization. Unlike lattice growth during crystallization, this process requires high-temperature melt quenching followed by rapid cooling, enabling precise multi-stage control through voltage pulse tuning.~~ This observation, however, does

not imply that amorphization is inherently more controllable than crystallization. In fact, crystallization typically proceeds more gradually, and extensive prior work has successfully demonstrated multi-level crystallization in Sb_2Se_3 by carefully tuning electrical pulse parameters [47]. Yet, the characteristic nucleation-and-growth mechanism of Sb_2Se_3 introduces inherent spatial stochasticity, making precise and repeatable control over intermediate crystalline states challenging [55]. In contrast, the amorphization ("reset") process, although more abrupt, is governed by a sharper thermal threshold, requiring the material to briefly exceed its melting point. The absence of a stochastic nucleation step in this regime offers a distinct pathway for multi-level control. Previous studies have shown that by progressively increasing the amorphization pulse amplitude, multiple distinct re-amorphization levels can be achieved, sometimes yielding finer gradation than crystallization under similar conditions [49]. Our own experimental data align with this trend, demonstrating multi-level amorphization with a finer apparent gradation than the crystallization levels obtained under our specific driving scheme. Nevertheless, it is important to emphasize that neither our results nor existing literature conclusively prove that one process is fundamentally more controllable than the other. The observed behavior is highly dependent on material composition, device geometry, and driving conditions. The prevailing view in the field maintains that achieving truly precise and deterministic intermediate states will likely require active feedback mechanisms, such as in situ optical or electrical monitoring, to complement and guide both crystallization and amorphization pulses in a coordinated manner [49].

Reviewer#1, Comment # 7:

Could the authors specify what they mean by "large-sized" metasurface pixels in lines 315-317?

Author response:

We thank the reviewer for raising this point for clarification. By "large-sized" metasurface pixels (lines 315-317), we refer to the relatively large area of each independently controlled pixel within the electrically addressed Sb_2Se_3 metasurface array, not to the dimensions of the individual nanoscale voids constituting the metasurface.

In the current work, each optically functional metasurface pixel measures 75 μm×75 μm. To ensure effective and uniform heating coverage, the underlying Ti heating electrode for each pixel is designed with an area of 85 μm×100 μm, slightly larger than the optical pixel. However, as is characteristic for such large-area square heaters, achieving perfectly uniform heating is challenging; a localized "hot spot" tends to form near the center of the electrode. The electro-thermal simulations of our Au/Ti micro-heater under driving pulses, included in the revised manuscript, further confirm the presence of a localized hot spot and significant thermal gradient. This heating non-uniformity is identified as a key factor limiting the controllability and uniformity of the electrically induced phase transition in the current design. Prior studies have shown that optimizing the geometry of the heater's active region can effectively improve thermal uniformity, which represents an important direction for our subsequent device optimization (Nature Nanotechnology, 2021, 16(6): 661-666).

To avoid any ambiguity, we have revised the manuscript to explicitly state the pixel dimensions and have clarified the intended meaning of "large-sized" in this context.

Corrections:---

(line 313-317, Main article): Modified

It is believed in this study that the main reason for the limited crystallization level during rapid crystallization is the inevitable non-uniform heating of the underlying thermal electrode during the heating process. ~~Especially when dealing with large-sized metasurface pixels, crystallization pulses with narrow pulse width and high voltage tend to create significant local hotspots, which in turn restrict the overall crystallization of the metasurface pixels.~~ Especially for metasurface pixels built on large-footprint heaters, crystallization pulses with narrow width and high voltage tend to create pronounced local hot spots, which in turn limit the overall crystallization uniformity across the pixel. This thermal gradient stems directly from the extended area of the Au/Ti micro-heater and is clearly illustrated in the accompanying electro-thermal simulations (Please refer to Supporting Information S1). We regard such heater-induced temperature non-uniformity as a key factor constraining the controllability of the phase transition in the current geometry. Previous works have shown that optimizing the heater morphology can effectively mitigate this issue, pointing toward a promising direction for further improving the performance of electrically driven phase-change metasurfaces [44].

Reviewer#1, Comment # 8:

In the last section of the paper, the authors demonstrate the spectral reconstruction performance in two configuration with static and dynamic operation of the device. Here, the flow of the discussion makes it hard to follow how the original metasurface pixel design was chosen. In particular, the figure captions are too concise and miss some relevant information. I believe that the pixel design is fixed by what is presented in Fig. 4c for static configuration (the vertical axis - radius - is, I assume, the radius of the holes in Sb2Se3 metasurface, but is not specified anywhere) and in Fig. 5a right (here, the vertical axis is index, which is even more confusing). Was these designs optimized somehow or just chosen for uniform coverage of the region of interest with spectral features of the metasurface? Why the particular metasurfaces in Fig. 5a were chosen to realize the tunable spectral reconstruction? Finally, does the data in Fig. 5b mean that using an 18-unit static metasurface arrangement shows exactly the same performance as a tunable arrangement of the same size, and the gain only happens when the number of units is increased?

Author response:

We sincerely thank the reviewer for this important question regarding the selection of metasurface unit designs in the spectral reconstruction section, which helps us clarify a key aspect of our work. We apologize for the lack of clarity in our original description and for the overly concise figure captions. In the revised manuscript, we have thoroughly rewritten the relevant section and updated the figure captions with the missing critical information (e.g., specifying that "radius" refers to the hole radius in the Sb_2Se_3 metasurface and clarifying "index" as "metasurface pixel index") to guide the reader more effectively.

As we mentioned in the manuscript, the core of computational spectral reconstruction lies in constructing a set of distinct spectral response vectors to form a transmission matrix for sampling an unknown spectrum. While prior works often use large arrays of dozens to hundreds of static metasurfaces to ensure sampling precision, this approach sacrifices spatial resolution (Advanced Materials, 2024, 36(23): 2313357). Our work explores a complementary pathway: using a small-scale, tunable phase-change metasurface array to expand the effective transmission matrix without increasing the physical pixel count, thereby maintaining high spatial resolution.

To demonstrate the effectiveness of this concept, we designed a comparative experiment between static and dynamic (phase-change) arrays of the same physical scale. First, a static 6×6 array was fabricated by varying the period and hole radius of the Sb_2Se_3 metasurface units, yielding 36 distinct amorphous-state spectral responses (Figure 4c). The specific geometric parameters were chosen simply to ensure their resonance wavelengths differed and collectively covered the band of interest, and no extensive optimization was performed. This static array successfully demonstrated spectral reconstruction as shown in Figure 4d, e and f in the manuscript, validating the baseline matrix

Regarding the construction of the dynamic (phase-change) transmission matrix for comparison, the 18 metasurface pixels used in Figure 5a were selected from the original 36-pixel static array based on a clear criterion: to ensure comprehensive spectral coverage across the target band (1100–1590 nm). We specifically chose pixels whose amorphous-state resonances were located in the short-to-middle wavelength regions (Zones B and C). The rationale is that when these pixels are switched to the crystalline state, their resonance peaks exhibit a significant redshift. This shift

effectively covers the middle-to-long wavelength regions of the band that their amorphous states did not cover. Thus, the combination of amorphous and crystalline spectra from this subset of 18 pixels provides relatively complete spectral sampling over the entire band of interest, despite using half the physical pixels. We acknowledge that this selection was not exhaustively optimized (e.g., for ultimate mutual correlation minimization) but was deliberately designed to directly validate our core concept: that phase-change tuning can effectively expand the transmission matrix.

To quantitatively compare the spectral sampling performance of the two 36-channel transmission matrices - one expanded by adding new static pixels and the other by leveraging phase-change tuning - we employed the mutual correlation coefficient of the matrices as a key metric. This analysis directly addresses the core of the comparison. Specifically, we treated the 18 amorphous-state spectra common to both matrices as a shared baseline. Starting from this baseline (size = 18), we then simulated the expansion of the transmission matrix in the two distinct ways and tracked the corresponding evolution of the mutual correlation coefficient. This methodological choice is precisely why the horizontal axis in Figure 5b begins at 18.

The results of this comparative analysis are presented in Figure 5b. The blue curve shows the trend when the matrix is expanded by sequentially incorporating the spectral responses of new static metasurface designs from the remaining pool. The red curve illustrates the trend when the matrix is expanded by instead incorporating the new spectral responses obtained from the phase change (crystalline state) of the same 18 selected pixels. The data indicates that both methods can expand the matrix while maintaining a relatively low mutual correlation. Notably, in our experimental setup, the phase-change-based expansion appears to yield a slightly lower mutual correlation coefficient as the matrix size increases, suggesting a potentially more efficient spectral sampling capability. The subsequent spectral reconstruction experiments (Figure 5e) corroborate this observation, showing a minor reduction in the mean squared error for the reconstruction using the phase-change-based matrix.

We emphasize that the central purpose of this comparative analysis is not to claim the universal superiority of one expansion method over the other. Rather, it robustly demonstrates that utilizing the dynamic spectral modulation of phase-change metasurfaces provides an effective and viable pathway to expand a transmission matrix. This capability is pivotal because it enables high-precision spectral reconstruction with a compact, small-scale physical array, thereby preserving spatial resolution. Our experimental results confirm that a strategically designed, tunable array can achieve a sampling capacity and reconstruction accuracy comparable to those of a larger static array. This work thus validates a promising approach toward miniaturized and high-performance spectroscopic systems, where phase-change tuning unlocks additional dimensionality in the design of compact photonic sensors.

We have incorporated this detailed explanation and discussion into the revised manuscript to ensure our methodology and conclusions are clearly presented. We thank the reviewer again for prompting this significant clarification.

Corrections:---

(line 350-361, Main article): Modified

Spatial-spectral reconstruction via metasurface arrays fundamentally relies on constructing a high-dimensional spectral transmission matrix with distinct spectral responses. ~~Conventional techniques demand large-scale arrays to ensure reconstruction accuracy, which severely degrades spatial~~

resolution and hinders applications in spectral imaging [58-59]. Active metasurfaces address this limitation by dynamically enriching the transmission matrix dimensionality. Using the giant optical contrast of Sb_2Se_3 PCM, we reconfigure spectral responses across phase states to compress the required array size. We validate this approach by comparing two 36-dimensional transmission matrices: one from the static 6×6 array, another from just 18 Sb_2Se_3 units electrically switched between two phase states. This demonstrates how dynamic response tuning enables high-precision reconstruction with compact arrays. Following a baseline demonstration using static metasurfaces, we subsequently incorporate electrical phase switching to establish the feasibility of miniaturized high-accuracy reconstruction systems. While conventional approaches require large-scale static arrays to ensure sampling accuracy, this inevitably compromises spatial resolution and impedes applications in spectral imaging [58-59]. To overcome this limitation, we propose and implement a strategy that utilizes the proposed tunable phase-change metasurfaces array. The core concept is to expand the effective dimensionality of the transmission matrix not by increasing the number of physical pixels, but by leveraging the giant optical contrast between the amorphous and crystalline states of a single pixel, thereby preserving a compact form factor and high spatial resolution.

To systematically evaluate the potential of active tuning, we established a comparative framework designed to decouple the contributions of physical array scale from those of spectral reconfigurability. This framework involves constructing two transmission matrices of identical order (36 spectral responses) via fundamentally different strategies, one static and one dynamic, and then comparing their performance in spectral reconstruction. The static transmission matrix is constructed directly from the amorphous-state spectral responses of all 36 pixels in our fabricated 6×6 Sb_2Se_3 metasurface array. The geometric parameters (period and hole radius) of these units were varied such that their resonance wavelengths differ and collectively span the target near-infrared band. It is important to note that this set of 36 designs was not extensively optimized for ultimate reconstruction performance; its primary purpose is to provide a baseline matrix generated purely through spatial multiplexing of fixed spectral features. The dynamic transmission matrix is built using only 18 physically distinct pixels selected from the same 6×6 array. The selection criterion was to ensure comprehensive spectral coverage: we chose pixels whose amorphous-state resonances are located primarily in the short-to-middle wavelength regions. When electrically switched to their crystalline state, these same pixels exhibit substantial resonance redshifts, thereby covering the middle-to-long wavelength regions of the band. Thus, by utilizing both the amorphous and crystalline states of this subset, we generate a complete set of 36 distinct spectral responses. This selection was not exhaustively optimized for parameters such as mutual correlation but was deliberately designed to validate the core premise: that phase-change-induced spectral shifting can functionally expand the dimensionality of a transmission matrix without increasing the number of physical pixels.

(line 363, Main article): Added

Our experimental demonstration begins by validating the spectral reconstruction performance using a static metasurface array. The detailed spectral reconstruction process is shown in Figure 4a.

(line 408-416, Main article): Modified

It is worth noting that the significant optical contrast between the amorphous and crystalline states

~~of Sb_2Se_3 enables a substantial difference in the spectral responses of the metasurfaces. This characteristic can be leveraged to enrich the spectral transmission matrix by incorporating the active spectral response of the phase-change metasurface, thereby further enhancing the non-correlation of the spectral transmission matrix. In other words, by integrating phase-change modulation of the metasurface pixels, the current work can reduce the required number of metasurface pixels for spectral reconstruction while maintaining broadband spectral sampling capabilities. Here, taking second-order phase change control as an example, the current work further reduces the required number of metasurface pixels for high-precision broadband spectral reconstruction to 18.~~ Following the validation of the 36-channel transmission matrix constructed from the static metasurface array, which demonstrates effective spectral reconstruction across the 1100–1590 nm band, we next use its performance as a baseline for comparison. We then evaluate the reconstruction capability of a second 36-channel matrix built from only 18 physical pixels by leveraging both their amorphous and crystalline states. This direct comparison within the identical spectral window allows us to assess the effectiveness of using phase-change tuning to expand the dimensionality and sampling capacity of a compact metasurface array. Figure 5a shows the selected 18 metasurface pixels, and a ~~36-order~~ 36-channel spectral transmission matrix is constructed using their reflection spectra at both the amorphous and crystalline states.

(line 421-441, Main article): Modified

~~Here, we compare the cross-correlation of the 36-order spectral transmission matrix constructed using 36 metasurface pixels in previous work with that of the matrix constructed here using second-order phase change to qualitatively assess the effectiveness of enriching the spectral transmission matrix through phase-change spectral modulation. To facilitate the quantitative characterization of the complexity of the transmission matrix, we propose a novel method for calculating the cross-correlation coefficient, which we refer to as the spectral cross-correlation coefficient.~~ Here, we introduce and apply a spectral cross-correlation coefficient as a quantitative metric to evaluate and compare the spectral sampling performance of the two 36-channel transmission matrices. In essence, the spectral cross-correlation coefficient evaluates the broadband sampling capability by measuring the average changes in the transmission matrix at different wavelengths within the target spectral range (Detailed calculation process is provided in the Supporting Information ~~S6~~S9). Figure 5b shows the variation of spectral cross-correlation with the expansion of the matrix size during the construction of the two types of spectral transmission matrices. Specifically, we treated the 18 amorphous-state spectra common to both matrices as a shared baseline. Starting from this baseline (size = 18), we then simulated the expansion of the transmission matrix in the two distinct ways and tracked the corresponding evolution of the spectral cross-correlation coefficient. This methodological choice is precisely why the horizontal axis in Figure 5b begins at 18. The blue curve shows the trend when the matrix is expanded by sequentially incorporating the spectral responses of new static metasurface designs from the remaining pool. The red curve illustrates the trend when the matrix is expanded by instead incorporating the new spectral responses obtained from the phase change (crystalline state) of the same 18 selected pixels. The data indicates that both methods can expand the matrix while maintaining a relatively low mutual correlation. Notably, the phase-change-based expansion appears to yield a slightly lower mutual correlation coefficient as the matrix size increases, suggesting a potentially more efficient spectral sampling capability. ~~Evidently, the introduction of phase-change spectral modulation markedly increases the complexity of the~~

~~transmission matrix, outperforming the direct augmentation of the number of metasurface pixels. This enhancement suggests the potential for higher-precision spectral reconstruction.~~ As illustrated in Figures 5c, 5d, and 5e, the current work experimentally validates high-precision single-wavelength identification and broadband spectral reconstruction in the short-wave infrared band using the phase-change spectral transmission matrix. The average error for single-wavelength identification is as low as 0.325 nm, while the MSE for broadband spectral reconstruction is maintained below 0.004. Both metrics surpass the reconstruction accuracy achievable with a static spectral transmission matrix, thereby underscoring the distinct advantages of the phase-change metasurface array in spatial spectral reconstruction.

The central purpose of this comparative experiment is not to prove that expanding the matrix via phase-change modulation necessarily yields superior reconstruction performance compared to incorporating additional, distinct metasurface units. Rather, it robustly demonstrates that utilizing the dynamic spectral modulation of phase-change metasurfaces provides an effective and viable pathway to expand a transmission matrix. This validation underscores the significant potential of tunable metasurface arrays for achieving high-performance spectral reconstruction with a compact, small-scale physical footprint.

(Figure 4, Main article): Modified

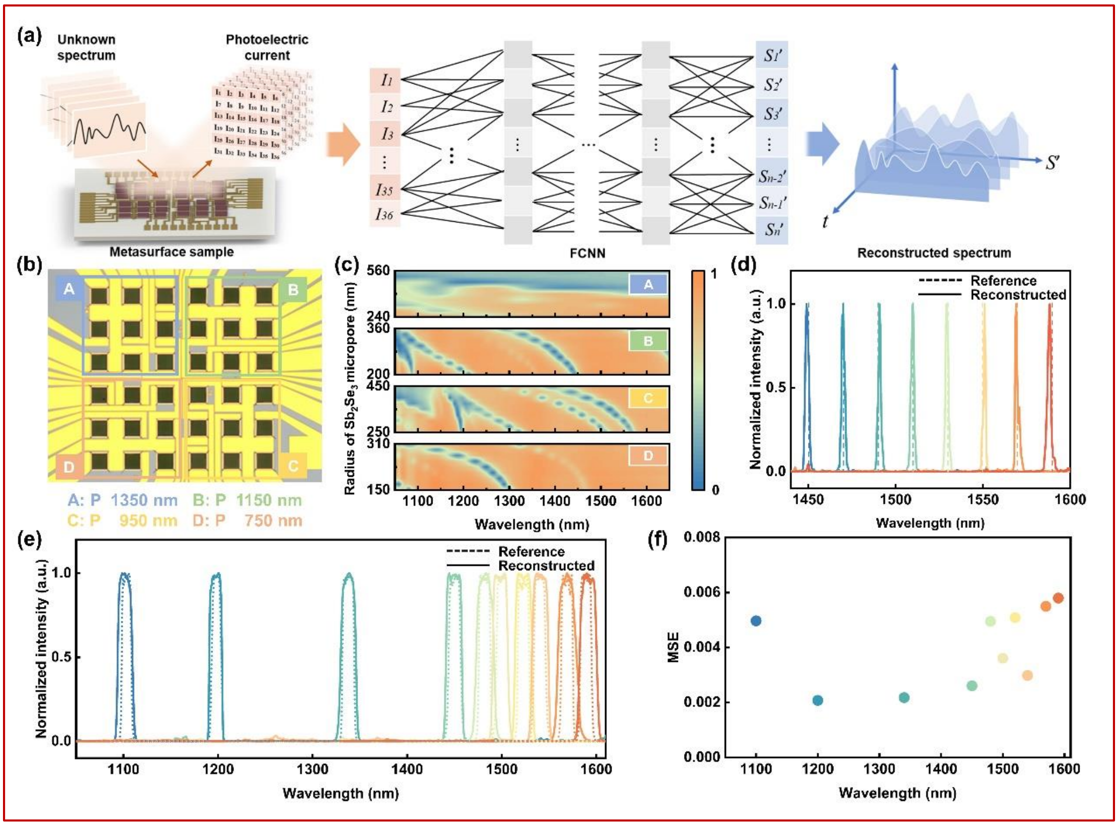

Figure 4. Experimental validation of spatial spectral reconstruction using the 6×6 Sb_2Se_3 metasurface array. a, Schematic illustration of the spatial spectral reconstruction process based on the metasurface array. b, Microscopic image of the fabricated metasurface array for spatial spectral reconstruction. c, Experimentally measured ~~S~~spectral transmission matrix constructed from the reflection spectra of the metasurface array. d, Performance validation of single-wavelength spectral

reconstruction within the wavelength range of 1450–1590 nm. e, Reconstruction of the transmission spectra of narrowband filters within the wavelength range of 1100–1590 nm. f, Mean squared error (MSE) of the reconstructed transmission spectra of narrowband filters with different central wavelengths.

(Figure 5, Main article): Modified

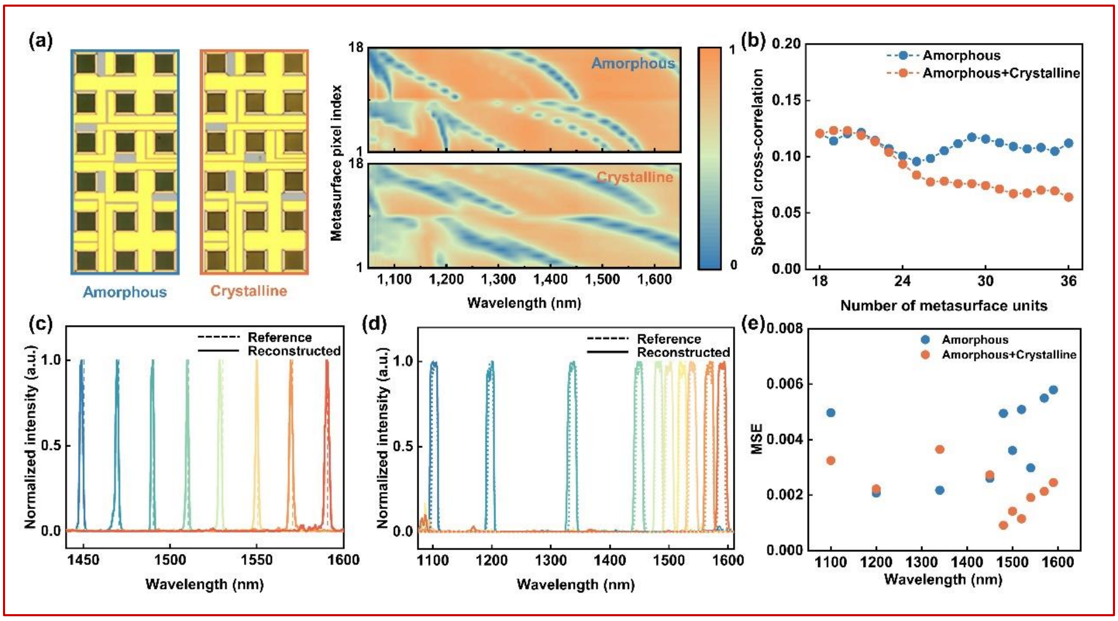

Figure 5. Validation of spatial spectral reconstruction performance using a reduced-scale array considering phase-change spectral modulation. a, Microscope images of the selected 18 Sb_2Se_3 metasurface pixels before and after crystallization, and the ~~36-order~~ 36-channel spectral transmission matrix constructed using the reflectance spectra at amorphous and crystalline states. b, Evolution of the spectral cross-correlation coefficients of the transmission matrices for static and phase-change metasurface arrays with the increase of the size of the spectral transmission matrix. c, Single-wavelength detection performance within the 1450-1590 nm wavelength range using the phase-change spectral transmission matrix. d, Spectral reconstruction of transmission spectra for band-pass filters within the 1100-1590 nm range using the phase-change spectral transmission matrix. e, Comparison of the MSE for broadband spectral reconstruction between static and phase-change metasurface arrays.

Reviewer#2:

The manuscript addresses one of the key challenges that currently stand in the way of taking phase-change metasurfaces from the laboratory to real-world practical application, namely how to carry out selective (pixelated) in-situ switching. While several previous works have shown the use of integrated microheaters, both metal and ITO, to successfully switch phase-change metasurfaces, very few, if any, to date have shown the full implementation of pixelated metal heaters - though it is noted that the company Bodle Technologies Ltd developed and patented a pixelated heater array for phase-change display purposes (see e.g. patent application US 2025 0155734 A1). The current work is therefore novel and brings significant value to the community. However, the technical achievements presented in the paper do have some significant limitations, as recognised in most cases by the authors, including a small number of pixels (36), a relatively low fill factor (phase-change cell size/total pixel area) and relatively slow switching speeds which, in the best case, are in the 10s kHz range and limited by the time required for crystallization and the maximum chip duty cycle. Nonetheless, in spite of such limitations and in light of the importance of the manuscript' s subject matter (in-situ pixelated switching of pcm metasurfaces) and the quality of results presented, the paper would, in my opinion, be suitable for publication in Nature Communications subject to the following amendments, clarifications and modifications.

Author response:

We sincerely thank the reviewer for their positive assessment of the novelty and academic value of our work.

We fully acknowledge that several previous studies have successfully demonstrated electrically switched phase-change metasurfaces using on-chip microheaters. The central contribution of this work lies in the experimental realization of a pixelated, electrically addressable Sb_2Se_3 phase-change metasurface array and the initial exploration of its application potential for integrated spatial-spectral reconstruction.

We also agree with the reviewer that the present planar-wiring-based design inherently limits the array scale and fill factor. In response, we have enriched the discussion in the revised manuscript by introducing a vertically stacked, row-column addressing scheme supported by numerical simulations, which verifies the feasibility of independent pixel control. This proposed architecture offers a tangible pathway to overcome the current limitations in scalability and fill factor and represents a key focus of our ongoing research.

Furthermore, we have diligently addressed all specific questions and suggestions raised by the reviewer in the revised manuscript, with corresponding clarifications and modifications incorporated throughout the text. We believe these revisions have significantly improved the clarity and completeness of the presentation.

Once again, we are grateful for the reviewer's insightful and constructive feedback.

Corrections:---

(Section S2, Supporting Information): Added

Section S2: Design and Thermal Simulation of the Row-Column Addressed Heater Array

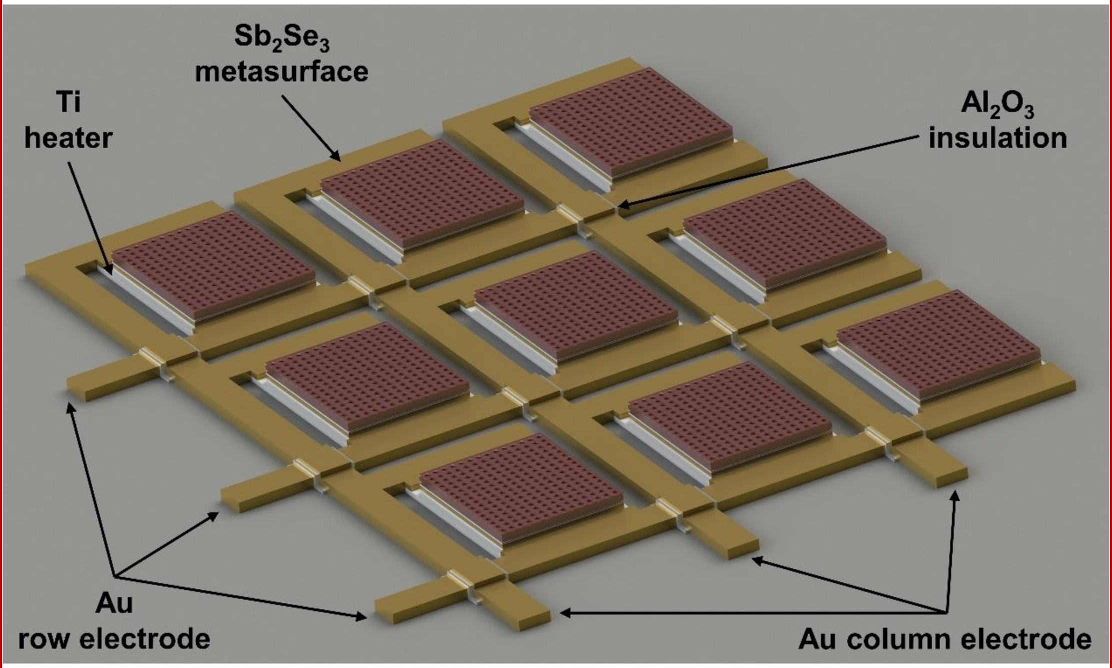

Figure S3. Schematic of the stacked architecture: a row–column addressed micro-heater integrated with a Sb_2Se_3 metasurface array.

As illustrated in Figure S3, we designed a conceptual stack employing a Ti/Au scheme analogous to our current work: Ti as the heating pad and Au as the interconnect lines. This row-column architecture consists of a bottom Ti electrode layer, an intermediate row/column electrode layer (insulated by a 10-nm Al_2O_3 layer between the row and column electrode layer), and a top Al_2O_3 insulation layer, over which the hybrid Sb_2Se_3 metasurface is placed.

To assess the feasibility of the proposed vertically stacked, row-column addressing scheme for electrically driving Sb_2Se_3 metasurface arrays, we performed numerical electro-thermal simulations using COMSOL Multiphysics. Although a modest 3×3 array was modeled for computational efficiency, the architecture inherently supports straightforward scaling to larger formats. The simulation investigated both a central and a corner pixel to account for potential positional variations in thermal response.

As shown in Figure S4, when appropriate row and column lines are selected and driven with optimized voltage pulses (e.g., 7.5 V, 50 μs for crystallization; 15 V, 8.33 μs for amorphization), the temperature of the targeted pixel, whether central or corner, rises sufficiently to reach the required crystallization (~473.15 K) and amorphization (~893.15 K) thresholds, with heating highly localized to the addressed site [1]. The temperature increase in neighboring pixels arises primarily from thermal diffusion and remains well below the crystallization point in both switching regimes. Specifically, during crystallization pulses, adjacent pixel temperatures stay significantly below 473.15 K. During the higher-power amorphization pulses, although neighboring pixels experience some heating via thermal spreading, their temperatures also remain below the crystallization threshold, thereby preserving their phase states. These results confirm that the row-column addressed heater can selectively switch individual pixels without unintentionally affecting their

neighbors, demonstrating its fundamental suitability for pixel-independent phase control.

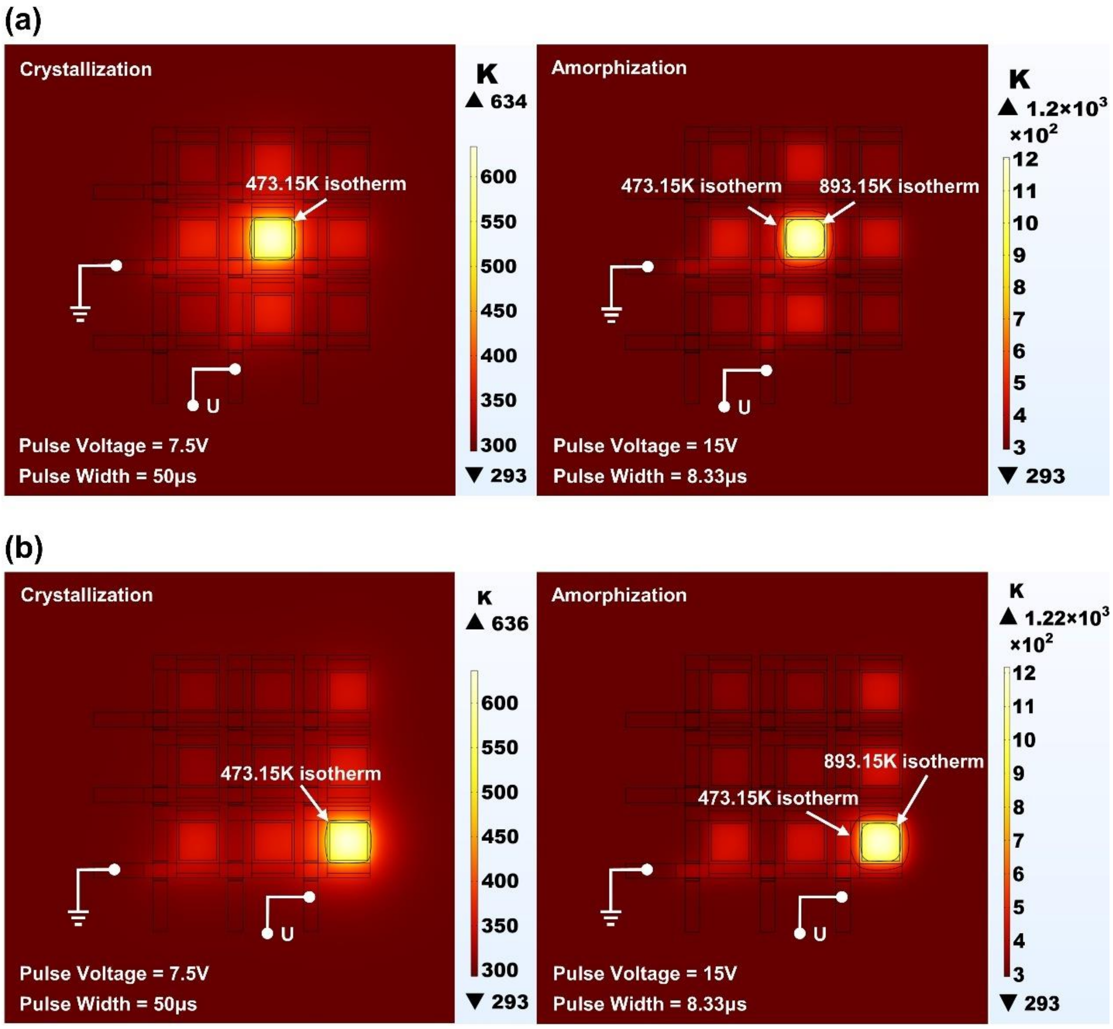

Figure S4. Independent thermal addressing of pixels in a 3×3 row-column array. Simulated temperature profiles when applying (a) crystallization (7.5 V, 50 μs) and amorphization (15 V, 8.33 μs) pulses to the center pixel, and (b) the same pulses applied to a corner pixel.

Moreover, compared to the planar-wiring scheme used in our current experimental array, the row-column architecture decouples fill factor from array size. In our simulated design, a fill factor of ~50% was readily achieved without dedicated optimization, and further improvement is attainable through layout refinement. This addresses the critical trade-off between fill factor and scalability inherent in planar routing.

Overall, our simulations validate the row-column addressing scheme as a promising route toward scalable, high-fill-factor, and independently controllable phase-change metasurface arrays. However, we also acknowledge that the row-column configuration involves a more complex, multi-layer fabrication process (requiring multiple lithography and alignment steps) compared to the single-layer planar wiring used in our present proof-of-concept demonstration. Therefore, while the row-column approach holds great promise for future high-density, large-scale arrays, we employed the more readily fabricated planar wiring in this initial work to successfully demonstrate the core

principle of randomly accessible, electrically driven phase-change metasurfaces.

Reviewer#2, Comment # 1:

Introduction section

1. In the introduction the references jump from #30 to #48, and ref 48 does not contain examples of the electrical switching discussed in the manuscript text (it features optical switching of integrated photonic devices). The authors should check that reference numbering is correct throughout the manuscript and reference in a systematic way.

Author response:

We thank the reviewer for bringing the inconsistencies in the reference numbering and citations to our attention. We sincerely apologize for this oversight. In the revised manuscript, we have carefully checked and corrected the numbering sequence of all references throughout the text. Specifically, the reference previously numbered as #48, which did not match the description of electrical switching, has been replaced with an appropriate citation (Nature, 2014, 511(7508): 206-211). We have also systematically reviewed all other references to ensure accuracy and consistency. We appreciate the reviewer's careful reading in helping us improve the manuscript.

Corrections:---

(Reference, Main article): Modified

[40] HOSSEINI P, WRIGHT C D, BHASKARAN H. An optoelectronic framework enabled by low-dimensional phase-change films[J]. Nature, 2014, 511(7508): 206-211.

[41] WANG Y, LANDREMAN P, SCHOEN D, et al. Electrical tuning of phase-change antennas and metasurfaces[J]. Nature Nanotechnology, 2021, 16(6): 667-672.

[42] HEMMATYAR O, ABDOLLAHRAMEZANI S, TAGHINEJAD H, et al. Electrically tunable phase-change metasurfaces using transparent conductive oxide microheaters[C]. CLEO: QELS_Fundamental Science. Optica Publishing Group, 2021: FTh4K. 1.

[43] POPESCU C C, ARYANA K, GARUD P, et al. Electrically reconfigurable phase-change transmissive metasurface[J]. Advanced Materials, 2024, 36(36): 2400627.

[44] ZHANG Y, FOWLER C, LIANG J, et al. Electrically reconfigurable non-volatile metasurface using low-loss optical phase-change material[J]. Nature Nanotechnology, 2021, 16(6): 661-666.

[45] POPESCU C C, PETERS M R A, MAKSIMOV O, et al. 2D Addressable Mid-infrared Metasurface Spatial Light Modulator[J]. arXiv preprint arXiv:2511.03583, 2025.

[46] BENTATA F, TAUTE A, LAPRAIS C, et al. Spatially-Controlled Planar Guided Crystallization of Low-Loss Phase Change Materials for Programmable Photonics[J]. Advanced Materials, 2025: e06609.

[47] WEI M, XU K, TANG B, et al. Monolithic back-end-of-line integration of phase change materials into foundry-manufactured silicon photonics[J]. Nature Communications, 2024, 15(1): 2786.

[48] FANG Z, CHEN R, FRÖCH J E, et al. Nonvolatile phase-only transmissive spatial light modulator with electrical addressability of individual pixels[J]. ACS nano, 2024, 18(17): 11245-11256.

[49] YANG X, LU L, LI Y, et al. Non-volatile optical switch element enabled by low-loss phase

change material[J]. Advanced Functional Materials, 2023, 33(42): 2304601.

[50] LI S Q, XU X, MARUTHIYODAN VEETIL R, et al. Phase-only transmissive spatial light modulator based on tunable dielectric metasurface[J]. Science, 2019, 364(6445): 1087-1090.

[51] GUO T, ZHANG Z, LIN Z, et al. Durable and programmable ultrafast nanophotonic matrix of spectral pixels[J]. Nature Nanotechnology, 2024: 1-9.

[52] KIM J Y, PARK J, HOLDMAN G R, et al. Full 2π tunable phase modulation using avoided crossing of resonances [J]. Nature Communications, 2022, 13(1): 2103.

[53] ZHANG J C, WU G-B, CHEN M K, et al. Electromagnetic wave tailoring: from one dimension to multiple dimensions [J]. Electromagnetic Science, 2023, 1(3): 1-21.

[54] LI Y R, LI Y, ZENG S, et al. Lossless Phase-Change Material Enabled Wideband High-Efficiency Spatial Light Phase Modulation at Near-Infrared [J]. Laser & Photonics Reviews, 2024, 18(11).

[55] HUANG Y S, LEE C Y, TAKEUCHI I, et al. Optical Phase Change Materials[J]. Annual Review of Materials Research, 2025, 55.

[56] WEI M, LI J, CHEN Z, et al. Electrically programmable phase-change photonic memory for optical neural networks with nanoseconds in situ training capability[J]. Advanced Photonics, 2023, 5(4): 046004.

[57] ALAM M S, LAING R, BOLATBEK Z, et al. Fast Cycling Speed with Multimillion Cycling Endurance of Ultra-Low Loss Phase Change Material (Sb2Se3) by Engineered Laser Pulse Irradiation[J]. Advanced Functional Materials, 2024, 34(14): 2310306.

[58] WEN J, HAO L, GAO C, et al. Deep learning-based miniaturized all-dielectric ultracompact film spectrometer[J]. Acs Photonics, 2022, 10(1): 225-233.

[59] ZHANG H, LI Q, ZHAO H, et al. Snapshot computational spectroscopy enabled by deep learning[J]. Nanophotonics, 2024, 13(22): 4159-4168.

Reviewer#2, Comment # 2:

In the section "Design and characterization of electrically-driven Sb2Se3 metasurfaces"
2. In the discussion on direct vs indirect heating, it seems that by 'direct' heating the authors mean Joule heating by electrical current injection into the phase-change material; if this is the case, the authors should specify this (i.e. specify the link between direct heating and current injection).

Author response:

We thank the reviewer for raising this point, which allows us to clarify an important definition. The reviewer's understanding is accurate: in our manuscript, the term "direct heating" specifically refers to the method where an electrical current is passed directly through the phase-change material itself, relying on Joule heating within the PCM to drive the phase transition. This approach is commonly used in phase-change memory to exploit the material's intrinsic fast switching properties (Science, 2012, 336(6088): 1566-1569). However, as the reviewer has inferred, applying such direct current injection to the larger, patterned PCM structures within a metasurface presents significant challenges for achieving uniform and controlled switching. Therefore, in our current work, we have adopted the more versatile indirect heating strategy, utilizing on-chip micro-heaters to thermally drive the phase transition of the Sb_2Se_3 metasurface.

In the revised manuscript, we have revised the relevant section to explicitly define "direct heating" in relation to current injection, thereby clarifying this distinction for the reader. We appreciate the suggestion to improve the precision of our terminology.

Corrections:---

(Line 115-117, Main article): Modified

~~Direct heating relies on the Joule heating effect of the phase-change material itself and is commonly used in GST-based phase-change materials, which is evidently not suitable for the near-insulating Sb_2Se_3 [50].~~ Direct heating operates by passing an electrical current directly through the phase-change material itself via external electrodes, where the resulting Joule heating within the material drives the phase transition. This method is commonly employed for conductive phase-change compounds such as GST, but is evidently unsuitable for near-insulating materials like Sb_2Se_3 [40].

Reviewer#2, Comment # 3:

3. When discussing the use of microheaters to switch phase-change metasurfaces, the authors should cite all the relevant papers, not just ref #49 as currently (see e.g., Y. Wang et al, Nature Nanotechnology, vol. 16, no. 6, pp. 667 – 672, 6 2021; C. C. Popescu et al, Advanced Materials, 2400627 2024; O. Hemmatyar et al, Conference on Lasers and Electro-Optics (2021), paper FTh4K.1, 5 2021.)

Author response:

We appreciate the reviewer's suggestion regarding the citation of relevant literature on microheater-driven phase-change metasurfaces. In response, we have incorporated the recommended references (Y. Wang et al., Nature Nanotechnology 16, 667 – 672, 2021; C. C. Popescu et al., Advanced Materials 36, 2400627, 2024; O. Hemmatyar et al., Conference on Lasers and Electro-Optics, FTh4K.1, 2021) into the revised manuscript. These additions provide a more comprehensive background and better contextualize our work within the field. Thank you for this helpful guidance.

Corrections:---

(line 66-75, Introduction): Modified

To overcome this, indirect electrical control using integrated micro-heaters ~~emerged, successfully demonstrating phase switching in materials like GST and GSST with larger pixel sizes reaching 0.4×0.4 mm² [49].~~ was developed. This approach has been successfully applied to various phase-change materials, including GST [41-42] and GSST [43-44], and validated for both transmissive [43] and reflective spatial light modulation [41-42,44].

Reviewer#2, Comment #4:

4. In Fig. 1, the pulse durations used for annealing and for melt-quench are the wrong way round. Also, why is the top surface of the phase-change layer in the stack shown as crystalline, with the rest of the layer as amorphous? I doubt that such a configuration is achieved during switching, so to show it here is likely to be confusing to the reader.

Author response:

We thank the reviewer for carefully reviewing the manuscript and pointing out these issues in Figure 1.

Regarding the pulse durations, we acknowledge and have corrected the inadvertent reversal of the crystallization (annealing) and amorphization (melt-quench) pulse labels in the revised version.

Concerning the coloring in the schematic illustration, we apologize for the confusion. The figure was intended to illustrate the three-dimensional shape of the metasurface structure, but during the drafting process, the color chosen for the top surface of the Sb_2Se_3 unit coincidentally matched the color scheme we used elsewhere to represent the crystalline phase. This was an unintended visual artifact and was not meant to imply that only the top surface was crystallized - a configuration that does not reflect the actual switching process. To eliminate any potential misinterpretation, we have adjusted the coloring in the revised figure.

We appreciate the reviewer's attention to detail, which has helped us improve the clarity and accuracy of the manuscript.

Corrections:--

(Figure 1, Main article): Modified

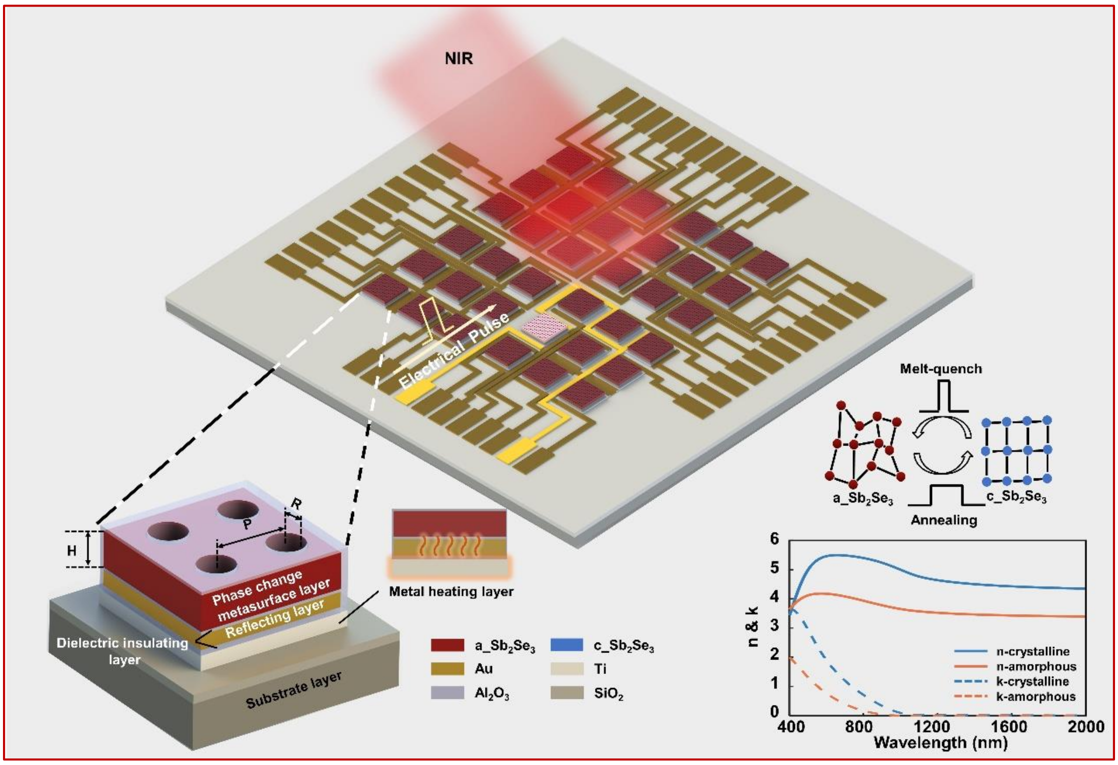

Figure 1. Schematic diagram of the 6×6 Sb_2Se_3 phase-change metasurface array.

Reviewer#2, Comment # 5:

5. Why was the choice made to limit to 6x6 pixel array? Was the size just limited by their fabrication facilities, or is it a more fundamental limit due to the array design? It would be helpful to include further discussion on the limitations of their array design in terms of scalability (how many pixels might be possible, how small the individual pixels might be made, how the fill factor could be improved).

Author response:

We thank the reviewer for this important question, which allows us to clarify the considerations behind the array scale and to discuss its scalability more thoroughly.

The choice of a 6×6 array in this work was primarily made to meet the requirement for a transmission matrix of sufficient size to demonstrate broadband spatial-spectral reconstruction, while remaining practical for design, fabrication, and characterization in this proof-of-concept study. It does not represent a fundamental physical limit of the phase-change material or the metasurface concept itself.

The primary limitation on scalability in the current implementation stems from the planar wiring scheme used for individual pixel addressing. In this design, the metal leads from each micro-heater must be routed through the gaps between pixels to the periphery of the array for wire bonding. As the number of pixels increases, the routing complexity grows rapidly. More critically, the need to allocate space for these inter-pixel leads forces a trade-off, causing the fill factor to decrease significantly with array size.

To address this fundamental challenge, we have expanded the discussion in the revised manuscript by introducing and analyzing an alternative vertically stacked, row-column addressing architecture. Numerical simulations (now included in the Supporting Information) confirm the feasibility of independent pixel control with this design. While our simulation example uses a 3×3 array for computational manageability, the row-column approach is inherently more scalable. It can, in principle, be extended to arrays comprising hundreds or even thousands of pixels without a proportional increase in routing complexity.

Furthermore, this architecture offers a clear path to a high fill factor. In a row-column design, the space between adjacent pixels needs to accommodate only a pair of narrow row/column lines (fabricated from low-resistivity Au). Since the heating occurs primarily in the higher-resistivity Ti pads above the line intersections, the Au lines can be made much narrower than the pixel pitch without significant parasitic heating, thereby maximizing the active area. Simulation results indicate a fill factor of ~50% can be easily achieved without dedicated optimization, and further improvement is attainable through layout refinement. While it is difficult to specify a precise maximum pixel count or optimal fill factor without a specific application target, we believe the proposed row-column addressing scheme provides a viable and promising technical pathway toward large-scale, high-density phase-change metasurface arrays. This constitutes a major focus for our future research.

We have incorporated this discussion on scalability and design trade-offs into the revised manuscript to provide a clearer perspective on the evolution of this technology.

Corrections:---

(line 150-158, Main article): Modified

In recent works, similar arraying schemes have also been used for pixelated electrical control of vanadium oxide active structures [54][51]. However, in comparison, Sb_2Se_3, GST, and other nonvolatile phase-change materials are current-driven active materials with high thermal thresholds, especially for the amorphization process. During the arraying process, the row and column electrode addressing scheme causes the driving current to follow a right-angle trajectory at the corresponding pixel, resulting in inevitable temperature non-uniformity within the pixel. Meanwhile, the addressing process of the array also introduces unavoidable time delays. Therefore, a phase-change metasurface array with random access capability for each pixel is necessary to fully exploit the high-speed response potential of phase-change materials. However, the row-column addressing scheme for driving non-volatile phase-change materials such as Sb_2Se_3, Sb_2S_3, and GST remains largely unexplored. In this work, inspired by previously reported vertically stacked row–column electrode architectures, we numerically validate the feasibility of the proposed scheme for selectively driving Sb_2Se_3 metasurfaces within a small-scale array through electro-thermal simulations (Please refer to Supporting Information S2 for detailed simulation results). Combined with its inherent compatibility with high fill factors and straightforward array scalability, the row-column addressing approach presents a promising route toward realizing electrically controlled phase-change metasurface arrays. Nevertheless, considering the fabrication complexity associated with vertically integrated row–column electrode stacks, the present work ultimately adopts a more readily fabricable alternative based on planar-wired micro-heaters.

(line 163-166, Main article): Modified

To address this, this work effectively reduces the overall number of required leads by sharing the ground line among pixels and optimizes the lead layout, demonstrating a two-dimensional random-access phase-change metasurface array with a pixel effective size of 75 μm and a pixel pitch of 150 μm, achieving an effective fill factor of nearly 50%.

We emphasize that the choice of a 6×6 array represents a deliberate practical balance for this proof-of-concept study. This scale provides a transmission matrix with sufficient dimensionality to validate the principle of broadband spatial-spectral reconstruction, while remaining manageable for the fabrication and characterization processes. It does not reflect a fundamental physical or architectural limit of the phase-change material or metasurface paradigm itself. The primary limitation on scalability in the current implementation stems from the planar wiring scheme used for individual pixel addressing. As noted, this design entails an inherent compromise between array size and fill factor: scaling the pixel count necessitates more interconnecting leads, which in turn reduces the usable active area. In contrast, the row-column addressed electrode architecture discussed in the Supporting Information S2 is designed to be compatible with both a high fill factor and straightforward array scaling. By vertically stacking the addressing lines and sharing them across rows and columns, this approach presents a promising and practical pathway for future research toward large-scale, densely integrated phase-change metasurface arrays.

Reviewer#2, Comment # 6:

6. The fill factor of the array is relatively low, at 50%. This, I presume, means that half the light incident on the array is not affected/modulated by the metasurface. Does this therefore mean that modulation efficiencies of 60% quoted by the authors are really only 30%, since half the light incident on the array is not affected by the metasurface?

Author response:

We thank the reviewer for this thoughtful question regarding the relationship between the array fill factor and the reported modulation efficiency. This allows us to clarify a potentially important point about the scope of our measurements.

The reviewer is correct that the overall array fill factor in our demonstration is approximately 50%, meaning roughly half of the light incident on the entire array area falls on the inter-pixel gaps. However, the modulation efficiencies of 60% quoted in the manuscript (e.g., in the context of Figure 2) refer to measurements performed on a single, isolated metasurface pixel. For these single-pixel optical characterizations (amplitude and phase modulation), the laser spot was carefully aligned to fully cover only the active metasurface area of an individual pixel, excluding the surrounding gaps and wiring. Therefore, the calculated modulation depth is intrinsic to the pixel's optical performance and is not diminished by the array's fill factor.

We acknowledge that this distinction could lead to misunderstanding. In the revised manuscript, we have added a clarifying note in the figure caption to explicitly state that the modulation efficiencies reported are based on single-pixel measurements, ensuring no ambiguity for the reader. We appreciate the reviewer's attention to this detail, which has helped us improve the precision of our data presentation.

Corrections:---

(Figure 2 Caption, Main article): Modified

Figure 2. Single-pixel ~~D~~dynamic optical modulation enabled by electrically-driven phase transition of Sb_2Se_3 metasurfaces. a, Microscope image of the fabricated electrically-driven Sb_2Se_3 phase-change metasurface array and scanning electron microscope (SEM) image of the micropore array within the metasurface. b, ~~Simulated and experimentally measured reflectance spectrum of the under-coupled resonant metasurface~~ Simulated (amorphous and fully crystalline) and experimentally measured (amorphous and electrically switched, partially crystallized) reflectance spectra of the under-coupled resonant metasurface designed based on the Sb_2Se_3 micropore array ~~at amorphous and crystalline states~~. The inset shows the simulated electric field distribution in the amorphous micropore structure supporting the under-coupled resonance mode. c, Experimentally measured reflectance spectrum of the Sb_2Se_3 metasurface under crystallization pulses with different voltages, demonstrating the multi-stage amplitude modulation effect at ~~1057 nm~~ 1577 nm due to multi-stage crystallization. d, Experimentally measured reflectance spectrum of the Sb_2Se_3 metasurface under amorphization pulses with different voltages, demonstrating the multi-stage amplitude modulation effect at ~~1057 nm~~ 1577 nm based on multi-stage amorphization. e, ~~Simulated and experimentally measured reflective phase spectrum of the over-coupled resonant metasurface~~ Simulated (amorphous and fully crystalline) and experimentally measured (amorphous

and electrically switched, partially crystallized) reflective phase spectra of the over-coupled resonant metasurface designed based on the Sb_2Se_3 micropore array ~~at amorphous and crystalline states~~. The inset shows the simulated electric field distribution in the amorphous micropore structure when the over-coupled resonance mode is excited. f, Experimentally measured reflective phase spectrum of the Sb_2Se_3 micropore array under crystallization pulses with different voltages, with the reflective phase modulation at 1500 nm during the multi-stage crystallization process highlighted. g, Experimentally measured changes in the reflectance during ~~500~~ approximately 60 phase-transition cycles, with the varying spectrum during ~~30~~ 10 selected phase-change cycles shown in detail. Crystallization was driven by a 5.3 V, 1 s pulse, and amorphization was driven by a 14 V, 8.33 μs pulse.

Reviewer#2, Comment # 7:

7. 500 switching cycles are shown in Fig. 2g, but what happens after 500 cycles, for example did the array stop working, or did the authors just stop testing? What is the expected maximum number of cycles that might be achieved, and what limits this maximum number? Note also that the number 500 on the axis of this figure has been cropped and so reads as 50.

Author response:

We sincerely thank the reviewer for raising these critical questions regarding the endurance characteristics and limitations of our electrically switched phase-change metasurface. They allow us to provide a more detailed discussion on device reliability, which is crucial for assessing practical potential. We also appreciate the note regarding the cropped axis label in the original figure, which we have corrected in the revised manuscript.

In response, we have conducted a more comprehensive investigation into the cycling performance. The initial test was stopped at 500 cycles as a practical checkpoint for intermediate characterization, not because of a sudden device failure. Our updated and extended tests reveal that the endurance is governed by two distinct factors, depending on the operational conditions.

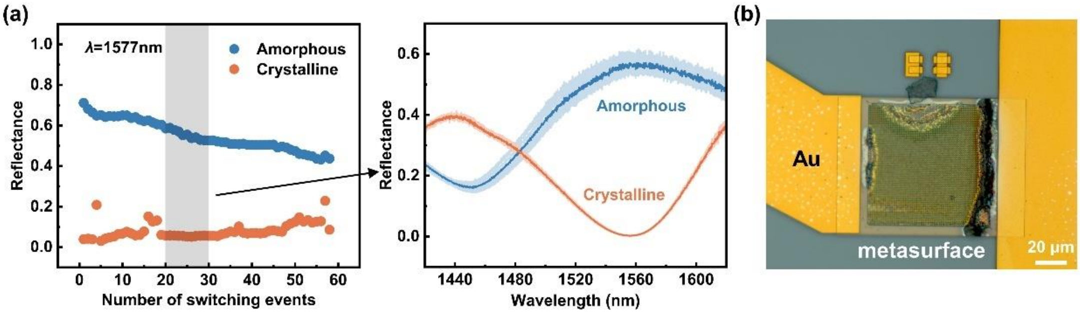

Figure R3. Cyclic optical response and electrode fracture under high-voltage switching. (a) Reflectance changes over approximately 60 phase-change cycles driven by a 5.3 V, 1 s crystallization pulse and a 14 V, 8.33 μs amorphization pulse. (b) Optical micrograph of the fractured micro-heater after approximately 60 cycles.

Under relatively higher driving voltages (e.g., 5.3 V for crystallization, 14 V for amorphization), the device can achieve effective switching for approximately 60 cycles before failure as shown in Figure R3. Post-failure analysis indicates that this failure is primarily mechanical, originating from the Au-Ti electrode interface. The inevitable step at this interface, combined with the localized heating in the Ti pad and the significant difference in thermal expansion coefficients between Au and Ti, creates substantial thermomechanical stress during high-voltage pulses, ultimately leading to electrode fracture. Importantly, we verified via laser-assisted phase switching that the Sb_2Se_3 metasurface itself retains its phase-change capability even after this electrode failure as given in Figure R4, confirming that the material is not the limiting factor in this regime.

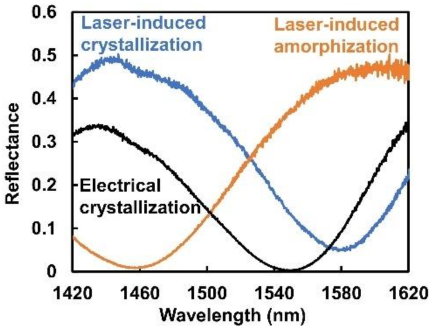

Figure R4. Phase-change functionality before and after electrode fracture. Reflection spectra after: electrical crystallization (before fracture, black); laser-induced amorphization (immediately after fracture, orange); and subsequent laser-induced crystallization (blue), demonstrating retained metasurface operability.

By employing slightly lower driving voltages (4.7 V and 13 V), we circumvent this mechanical failure and observe a different, gradual degradation mode. As shown in Figure R5, in this regime, the device sustains over 850 cycles without electrode damage. However, the optical modulation depth progressively decays. Detailed spectral analysis shows that this decay stems primarily from a diminishing amorphization efficiency - the re-amorphized state's resonance gradually redshifts with cycling, while the crystalline state remains stable. We attribute this not to gross morphological changes in the metasurface (which would also affect the crystalline spectrum), but to a cumulative effect of the intrinsic thermal non-uniformity of our large-area micro-heater. The localized "hot spot" likely alters the local phase-change kinetics over many cycles, progressively reducing the completeness of amorphization under a fixed voltage pulse.

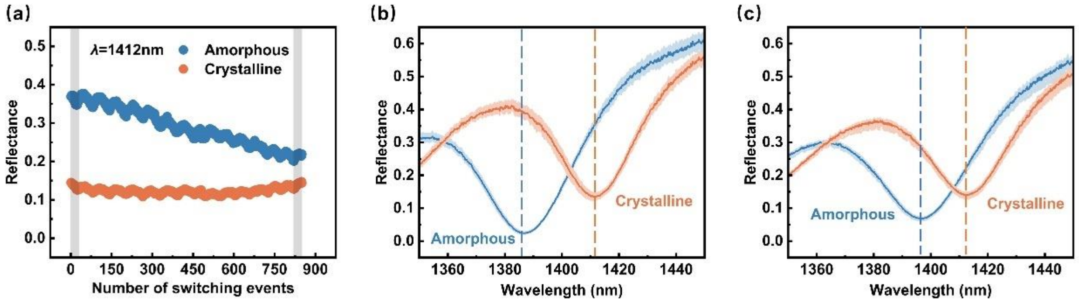

Figure R5. Cycling endurance of the Sb_2Se_3 metasurface under low-voltage electrical driving. (a) Evolution of reflectance at 1412 nm over 850 switching cycles driven by 4.7 V, 1 s (crystallization) and 13 V, 8.33 μs (amorphization) pulses. Corresponding reflection spectra for (b) the first 30 cycles and (c) the last 30 cycles.

Therefore, the expected maximum number of cycles and its limiting factors are highly condition-dependent in the current device. It is important to note that the ultimate endurance limit for the Sb_2Se_3 material itself is known to be far higher than the numbers reported here. Prior work in optimized waveguide platforms utilizing electrothermal driving has already demonstrated stable cycling exceeding 10^4 times (Advanced Functional Materials, 2023, 33(42): 2304601). This key insight confirms that the core phase-change medium is not the fundamental bottleneck. Consequently, the primary challenge for our present electrical metasurface architecture lies in the heater design. By systematically improving the mechanical robustness of the electrode interfaces to prevent fracture and optimizing the thermal uniformity of the micro-heater to ensure consistent

phase transitions, we can reasonably expect future iterations of our electrically controlled Sb_2Se_3 metasurface to approach the excellent intrinsic endurance of the material.

We have added this nuanced discussion on cycling performance and its underlying mechanisms to the revised manuscript, clarifying the distinction between electrode-driven and material/thermal-process-driven limitations. We thank the reviewer again for prompting this valuable deeper analysis.

Corrections:--

(line 261-266, Main article): Modified

~~It should be noted that in this work, a relatively wide pulse with a duration of 2 s was selected for the crystallization process. Although the same crystallization effect can be achieved using high-voltage, narrow-width electrical pulses, a mild crystallization process is expected to enhance the durability of the Sb_2Se_3 metasurface pixel. Utilizing a combination of 2-s crystallization pulses and 8.33-μs amorphization pulses, we demonstrated 500 stable phase-change cycles, as shown in Figure 2g.~~ In addition, we systematically evaluated the cycling endurance of the electrically driven Sb_2Se_3 metasurface. Under higher driving voltages (5.3 V, 1 s for crystallization; 14 V, 8.33 μs for amorphization), stable phase switching was achieved for approximately 60 cycles before failure (see Figure 2g). This failure was primarily mechanical, resulting from thermomechanical stress at the Au-Ti electrode interface, as evidenced by post-cycling microscopy (Supporting Information S6). Post-failure laser-assisted switching confirmed that the phase-change capability of the metasurface material itself remained intact, indicating that the material was not the fundamental limit. Subsequently, we employed lower driving voltages (4.7 V and 13 V). Under these conditions, the device sustained over 850 switching cycles without electrode damage, although a gradual decay in optical modulation depth was observed (Please refer to Supporting Information S6). Spectral analysis attributes this decay primarily to reduced amorphization efficiency, which we link to cumulative thermal non-uniformity (localized hot spots) in the micro-heater over repeated cycling. It is important to note that these cycle counts do not represent a fundamental limit of the Sb_2Se_3 material. Prior work utilizing integrated photonic platforms has demonstrated stable electrothermal cycling exceeding 10^4 times for Sb_2Se_3 [49]. Therefore, we anticipate that the cycling endurance of our electrically controlled metasurface can be significantly enhanced by future engineering optimizations focused on improving the mechanical robustness of the heater interface and the thermal uniformity of the micro-heater design.

(Section S6, Supporting Information): Added

Section S6. Extended Analysis of Cycling Endurance in Electrically Driven Sb_2Se_3 Metasurfaces

This section provides a detailed investigation into the cycling endurance of our electrically driven Sb_2Se_3 metasurface, extending the discussion from the initial results presented in Figure 2g of the main text. We examine the mechanisms limiting cycle life, beginning with the failure observed under high-voltage driving. As shown in Figure S9, optical microscopy of the device after approximately 60 cycles under high driving voltages (5.3 V for crystallization, 14 V for amorphization) reveals a physical fracture at the Au-Ti electrode interface. This mechanical failure is attributed to thermomechanical stress induced by the high-power pulses.

To confirm that this failure was confined to the heater and did not reflect degradation of the phase-

change material itself, we performed post-failure optical characterization using a laser-assisted switching platform, with the method detailed in our previous work [4]. Figure S10 shows the reflection spectra from the same metasurface pixel with the black curve corresponds to the electrically crystallized state obtained prior to heater fracture, while the orange and blue curves represent the states achieved via subsequent laser-induced amorphization and re-crystallization. The successful reversal of the phase transition confirms that the Sb_2Se_3 metasurface retains its intrinsic functionality, isolating the heater as the point of failure.

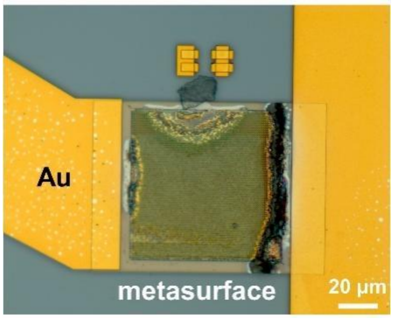

Figure S9. Optical micrograph of the fractured micro-heater after approximately 60 cycles.

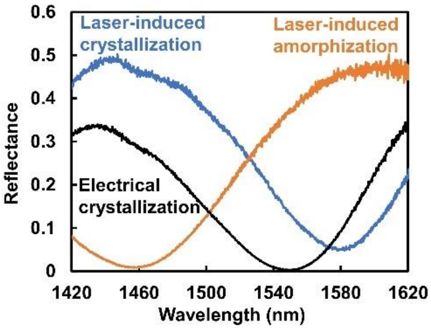

Figure S10. Phase-change functionality before and after electrode fracture. Reflection spectra after: electrical crystallization (before fracture, black); laser-induced amorphization (immediately after fracture, orange); and subsequent laser-induced crystallization (blue), demonstrating retained metasurface operability.

To probe the intrinsic cycling limits while avoiding mechanical failure, we evaluated a modified device under lower driving voltages (4.7 V for crystallization, 13 V for amorphization). The reflectance at 1412 nm was monitored over more than 850 switching cycles, as plotted in Figure S11a. The device maintained reversibility without electrode damage, indicating a significantly improved operational lifetime under these conditions. However, a gradual decay in the optical modulation depth was observed. Further analysis of the spectral evolution offers insight into this decay. Figure S11b and c compare the reflection spectra from the first 30 and the final 30 cycles, respectively. The data show a progressive redshift of the resonance in the re-amorphized state, while the crystalline-state resonance remains stable. This indicates a gradual reduction in amorphization efficiency. Such a decay in optical contrast is commonly observed in various phase-change photonic devices and has often been attributed to cumulative morphological changes in the active material during cycling [1]. However, we note that in our case, morphological change is unlikely to be the primary cause. The remarkable stability of the crystalline-state resonance throughout the test suggests that the overall metasurface geometry is preserved. The degradation is isolated to the

amorphization process. This observation, combined with our electro-thermal simulations presented in Supporting Information S2, which reveal pronounced heating non-uniformity specifically under the short, high-power pulses required for amorphization, leads us to attribute the gradual decay primarily to increasingly incomplete re-amorphization. We hypothesize that localized hotspots progressively alter the phase-change kinetics over many cycles, reducing the completeness and uniformity of the amorphized state formed under a fixed electrical pulse.

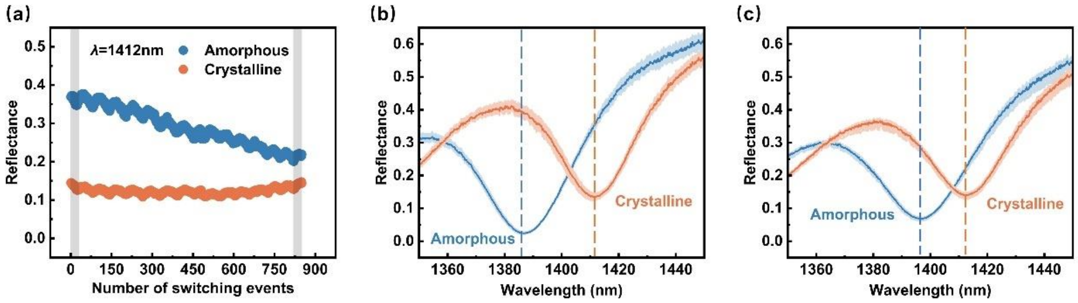

Figure S11. Cycling endurance of the Sb_2Se_3 metasurface under low-voltage electrical driving. (a) Evolution of reflectance at 1412 nm over 850 switching cycles driven by 4.7 V, 1 s (crystallization) and 13 V, 8.33 μs (amorphization) pulses. Corresponding reflection spectra for (b) the first 30 cycles and (c) the last 30 cycles.

The cycle counts reported here are not fundamental limits of the Sb_2Se_3 material, as prior work in integrated photonic platforms has demonstrated stable electrothermal cycling exceeding 10^4 times for similar phase-change systems. The performance of our present platform is therefore constrained primarily by heater reliability and thermal management. Future optimizations of the heater geometry for improved mechanical robustness and temperature uniformity are expected to enable cycling performance that approaches the intrinsic endurance of the phase-change material.

Reviewer#2, Comment # 8:

8. Were the results shown in Fig 2 obtained by simultaneously switching the whole array, i.e., all 36 pixels, or are they from a single or subset of pixels? This information should be clear to the reader.

Author response:

We thank the reviewer for raising this point for clarification. The results presented in Figure 2 were obtained by testing a single, individual pixel within the array, not by simultaneously switching all 36 pixels. We will make this condition explicit in the revised manuscript to ensure readers have a clear understanding of the experimental scope.

Corrections:---

(Figure 2 Caption, Main article): Modified

Figure 2. Single-pixel ~~D~~dynamic optical modulation enabled by electrically-driven phase transition of Sb_2Se_3 metasurfaces. a, Microscope image of the fabricated electrically-driven Sb_2Se_3 phase-change metasurface array and scanning electron microscope (SEM) image of the micropore array within the metasurface. b, ~~Simulated and experimentally measured reflectance spectrum of the under-coupled resonant metasurface~~ Simulated (amorphous and fully crystalline) and experimentally measured (amorphous and electrically switched, partially crystallized) reflectance spectra of the under-coupled resonant metasurface designed based on the Sb_2Se_3 micropore array ~~at amorphous and crystalline states~~. The inset shows the simulated electric field distribution in the amorphous micropore structure supporting the under-coupled resonance mode. c, Experimentally measured reflectance spectrum of the Sb_2Se_3 metasurface under crystallization pulses with different voltages, demonstrating the multi-stage amplitude modulation effect at ~~1057 nm~~ 1577 nm due to multi-stage crystallization. d, Experimentally measured reflectance spectrum of the Sb_2Se_3 metasurface under amorphization pulses with different voltages, demonstrating the multi-stage amplitude modulation effect at ~~1057 nm~~ 1577 nm based on multi-stage amorphization. e, ~~Simulated and experimentally measured reflective phase spectrum of the over-coupled resonant metasurface~~ Simulated (amorphous and fully crystalline) and experimentally measured (amorphous and electrically switched, partially crystallized) reflective phase spectra of the over-coupled resonant metasurface designed based on the Sb_2Se_3 micropore array~~ at amorphous and crystalline states~~. The inset shows the simulated electric field distribution in the amorphous micropore structure when the over-coupled resonance mode is excited. f, Experimentally measured reflective phase spectrum of the Sb_2Se_3 micropore array under crystallization pulses with different voltages, with the reflective phase modulation at 1500 nm during the multi-stage crystallization process highlighted. g, Experimentally measured changes in the reflectance during ~~500~~ approximately 60 phase-transition cycles, with the varying spectrum during ~~30~~ 10 selected phase-change cycles shown in detail. Crystallization was driven by a 5.3 V, 1 s pulse, and amorphization was driven by a 14 V, 8.33 μs pulse.

Reviewer#2, Comment # 9:

9. On page 6, last paragraph, the authors say …. the low phase-change threshold of the negative structure array …. helps to deliver a low driving voltage threshold. It is not clear what is meant by a low phase-change threshold – if this is the amorphization or crystallization temperature, then surely this just depends on the material and not on whether it is a negative or positive pattern (holes or cylinders). Some additional explanation/clarification is needed here.

Author response:

We thank the reviewer for this important question regarding the statement about a "low phase-change threshold" associated with our negative-pattern (hole-based) metasurface. We agree that the clarification is necessary and appreciate the opportunity to elaborate on the underlying rationale, which we have revised in the manuscript for greater precision.

In the context of our discussion, the intended meaning of "phase-change threshold" was the practically required thermal energy input to induce a phase transition in the metasurface unit cell, rather than the fundamental crystallization temperature of the bulk Sb_2Se_3 material. We acknowledge that the original wording was imprecise.

Our point relates to the observation that the ease of crystallization in nanoscale Sb_2Se_3 structures can be geometry-dependent. Recent studies on analogous Sb_2S_3 systems have shown that the probability of crystallization under a given thermal anneal decreases significantly as the physical dimensions of isolated phase-change features are reduced (Advanced Materials, 2025: e06609). This is attributed to the nucleation-limited crystallization mechanism: smaller volumes offer fewer stochastic nucleation sites, making crystallization more difficult. Given that Sb_2Se_3 follows a similar nucleation-and-growth mechanism, it is reasonable to expect a comparable size dependence.

This consideration informed our choice of a continuous hole-array ("negative") pattern over isolated pillar ("positive") structures. In our design, the Sb_2Se_3 forms a continuous network, effectively presenting a larger volume for nucleation per optical unit cell compared to an isolated nanopillar of sub-wavelength dimensions. We hypothesized that this could lower the practical thermal budget needed to reliably crystallize the metasurface. Supporting this, prior work on Sb_2Se_3 pillar-based metasurfaces reported requiring annealing at ~270°C for 30 minutes for significant crystallization (Advanced Science, 2023, 10(6): 2204899), which is notably higher than the typical crystallization temperature for bulk Sb_2Se_3 films (~200°C) (Advanced Functional Materials, 2023, 33(42): 2304601).

To ensure rigorous phrasing, we have revised the relevant sentence in the manuscript. We no longer state definitively that the negative structure lowers the threshold. Instead, we reference the size-dependent crystallization effects observed in related systems and present the continuous network as a design choice that may facilitate a more efficient crystallization process from an applied engineering perspective. We thank the reviewer again for prompting this more precise and accurate discussion.

Corrections:---

(line 211-213, Main article): Modified

~~Benefiting from the low resistance of the metallic microheater structure and the low phase-change threshold of the negative structure array, the current metasurface pixel has a relatively low driving~~

voltage threshold. In designing our metasurface, we selected a continuous, hole-based ("negative") pattern over an isolated pillar ("positive") architecture. This choice was motivated by considerations of phase-change kinetics at the nanoscale. Recent studies on similar systems suggest that the probability of crystallization under a given thermal stimulus can depend on feature size, with smaller, isolated volumes presenting fewer nucleation sites [46]. A continuous network, by comparison, offers a larger effective volume for nucleation per optical unit. While this does not alter the fundamental crystallization temperature of Sb_2Se_3, it may reduce the practical thermal energy required to reliably initiate and complete the transition within the metasurface lattice. This design aspect, combined with the low resistance of our integrated micro-heater, contributes to the relatively low driving voltages observed in our platform.

Reviewer#2, Comment # 10:

10. In the same paragraph, the authors talk about working in the 'weakly crystallized range' – further clarification of the meaning of this is needed. For example, do they mean crystallizing up to something like the 34% crystallization fraction mentioned earlier in the paragraph, but no further? And how does restricting the crystallization fraction (if indeed that is what is done here) ensure that random crystallization does not occur?

Author response:

We thank the reviewer for the insightful question regarding the term "weakly crystallized range," which helps us clarify an important aspect of our experimental methodology. We acknowledge that the original phrasing lacked precision.

In our work, "weakly crystallized" specifically refers to inducing and maintaining a low degree of crystallization, exemplified by the intermediate state with approximately 34% crystallization fraction mentioned in the same context. This state is not a fundamental limit of the material or heater but is actively selected by applying a crystallization pulse with a lower amplitude (and thus lower delivered energy). Our intent was to operate within a regime where the progression of crystallization is deliberately restrained.

The rationale is rooted in the spatially stochastic nature of nucleation in Sb_2Se_3 (Advanced functional materials, 2020, 30(36): 2002447). By limiting the overall crystallization fraction, we aim to mitigate the impact of this inherent randomness on the optical response. A lower crystallization fraction generally results in a smaller volume of the material undergoing the amorphous-to-crystalline transition, which can reduce the magnitude of optical deviations caused by non-uniform crystallization across the metasurface unit cell. However, we fully agree with the reviewer that this approach does not prevent random crystallization from occurring; it merely lessens its potential optical consequence at the specific operating point we targeted.

Therefore, the original claim that this method could "avoid the deviation... caused by random crystallization" was an overstatement. In the revised manuscript, we have corrected this description. We now clarify that operating in a weakly crystallized range is a strategy to reduce the sensitivity of the optical response to crystallization non-uniformity, not to eliminate the stochastic nucleation process itself. We appreciate the reviewer's careful attention to this nuance, which has improved the accuracy of our discussion.

Corrections:--

(line 221-225, Main article): Modified

~~Therefore, this work selectively controlled the PCM's working state within the weakly crystallized range to effectively avoid the deviation in the optical response caused by random crystallization and reduce the power consumption required for phase-change driving. Thanks to the significant optical contrast of Sb_2Se_3 before and after phase change, this work still achieved an amplitude modulation efficiency of nearly 60%.~~ Accordingly, this work deliberately operated the phase-change material within a weakly crystallized regime. This approach refers to the active selection and maintenance of a low crystallization fraction, approximately 40% state as mentioned above, by applying electrical pulses with reduced amplitude. While the inherent stochasticity of nucleation in Sb_2Se_3 cannot be eliminated, restricting the overall crystallized volume helps mitigate the optical impact of spatially

non-uniform crystallization within the unit cell. This strategy reduces the sensitivity of the optical response to such randomness at the targeted operating point, while also lowering the energy required per switching event. Leveraging the substantial optical contrast of Sb_2Se_3 between its amorphous and partially crystalline states, this approach enabled an amplitude modulation efficiency of nearly 80%.

Reviewer#2, Comment # 11:

11. Bearing in mind the comment above, are the results shown as for the 'crystalline' state in Fig. 2 for a fully, or weakly, crystallized state? This information should be made clear to the reader.

Author response:

We thank the reviewer for this important question, which highlights a lack of clarity in our original labeling. The reviewer is correct to note that the term "crystalline" in Figure 2 requires more precise definition.

In the revised manuscript, we have clarified this point explicitly. In Figure 2b, the simulation curves labeled "crystalline" correspond to the optical response of the Sb_2Se_3 metasurface in a fully crystallized state. In contrast, the experimental data points labeled "crystalline" in the same panel represent the state achieved under our specific driving condition of a 5.3 V, 1-second electrical pulse. Based on supporting simulations of the crystallization progression (provided in the Supporting Information), we estimate this experimentally achieved state to have a crystallization fraction of approximately 40%.

To prevent any potential misunderstanding, we have updated the caption of Figure 2 and the corresponding text in the results section to explicitly distinguish between these two definitions. We appreciate the reviewer's attention to detail, which has helped us improve the accuracy of our presentation.

Corrections:---

(Figure 2, Main article): Modified

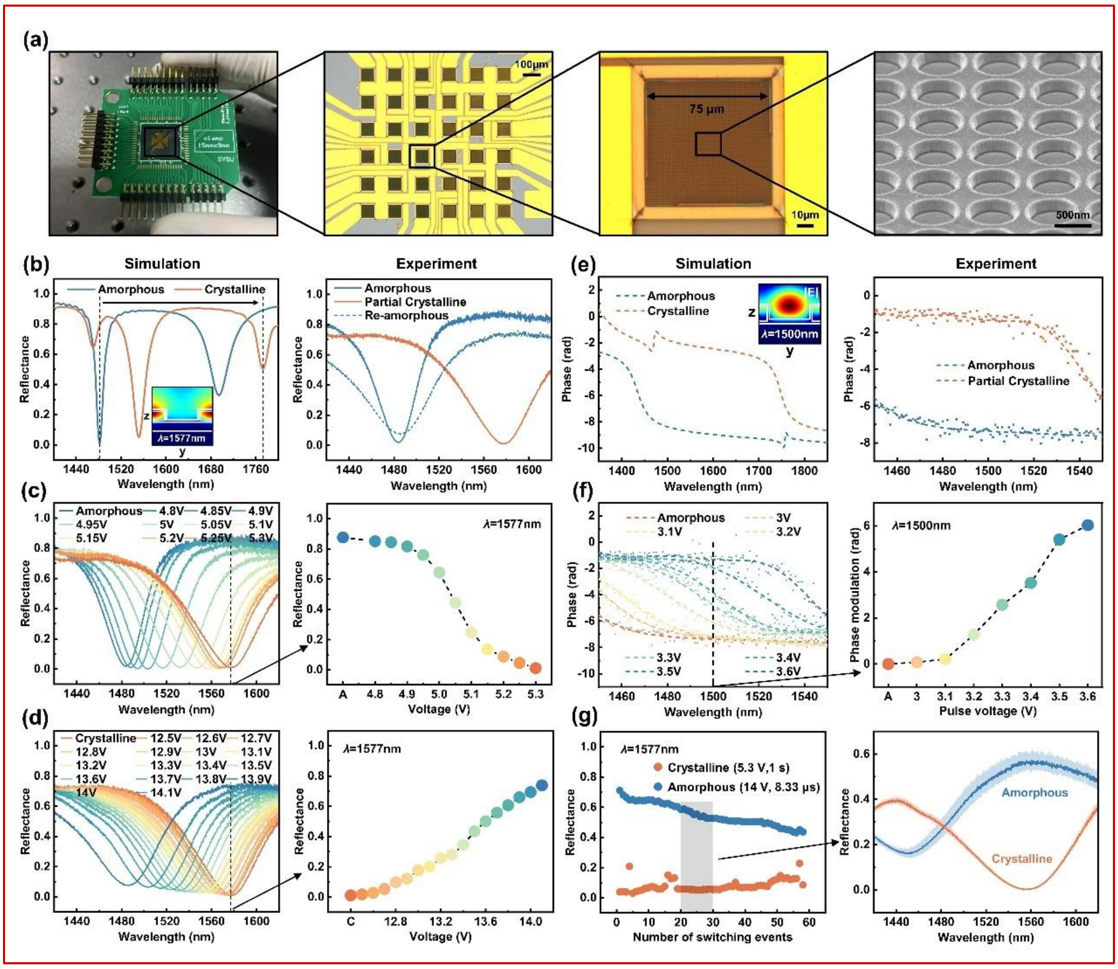

Figure 2. Single-pixel ~~D~~dynamic optical modulation enabled by electrically-driven phase transition of Sb_2Se_3 metasurfaces. a, Microscope image of the fabricated electrically-driven Sb_2Se_3 phase-change metasurface array and scanning electron microscope (SEM) image of the micropore array within the metasurface. b, ~~Simulated and experimentally measured reflectance spectrum of the under-coupled resonant metasurface~~ Simulated (amorphous and fully crystalline) and experimentally measured (amorphous and electrically switched, partially crystallized) reflectance spectra of the under-coupled resonant metasurface designed based on the Sb_2Se_3 micropore array ~~at amorphous and crystalline states~~. The inset shows the simulated electric field distribution in the amorphous micropore structure supporting the under-coupled resonance mode. c, Experimentally measured reflectance spectrum of the Sb_2Se_3 metasurface under crystallization pulses with different voltages, demonstrating the multi-stage amplitude modulation effect at ~~1057 nm~~ 1577 nm due to multi-stage crystallization. d, Experimentally measured reflectance spectrum of the Sb_2Se_3 metasurface under amorphization pulses with different voltages, demonstrating the multi-stage amplitude modulation effect at ~~1057 nm~~ 1577 nm based on multi-stage amorphization. e, ~~Simulated and experimentally measured reflective phase spectrum of the over-coupled resonant metasurface~~ Simulated (amorphous and fully crystalline) and experimentally measured (amorphous and electrically switched, partially crystallized) reflective phase spectra of the over-coupled resonant metasurface designed based on the Sb_2Se_3 micropore array~~ at amorphous and crystalline states~~. The inset shows the simulated electric field distribution in the amorphous micropore structure when the over-coupled resonance mode is excited. f, Experimentally measured reflective phase

spectrum of the Sb_2Se_3 micropore array under crystallization pulses with different voltages, with the reflective phase modulation at 1500 nm during the multi-stage crystallization process highlighted. g, Experimentally measured changes in the reflectance during ~~500~~ approximately 60 phase-transition cycles, with the varying spectrum during ~~30~~ 10 selected phase-change cycles shown in detail. Crystallization was driven by a 5.3 V, 1 s pulse, and amorphization was driven by a 14 V, 8.33 μs pulse.

Reviewer#2, Comment # 12:

12. Ref #45 discusses Sb2S3 materials, not the Sb2Se3 material used by the authors. Are the findings for the behaviour of Sb2S3 in ref. #45 transferrable to Sb2Se3? If so, the authors should say so and state why.

Author response:

We thank the reviewer for raising this point regarding the applicability of reference #45 (which focused on Sb_2S_3) to our work on Sb_2Se_3. This is a valid consideration, and we appreciate the opportunity to clarify the rationale for the citation.

The reference was used to illustrate a general principle regarding crystallization stochasticity in one-dimensional chain-type chalcogenide materials, a class that includes both Sb_2S_3 and Sb_2Se_3. Both materials share highly similar one-dimensional crystalline structures and are governed by a comparable nucleation-and-growth crystallization mechanism (Advanced functional materials, 2020, 30(36): 2002447). Consequently, the phenomenon of nucleation-induced spatial randomness discussed for Sb_2S_3 is directly relevant to Sb_2Se_3 as well.

That said, we agree that for the specific context of our Sb_2Se_3 metasurface, citing direct evidence is more precise. Therefore, in the revised manuscript, we have replaced reference #45 with a more directly applicable study from our own prior work (Laser & Photonics Reviews, 2024, 18(11): 2400293), which provides a detailed discussion on the scattering losses in Sb_2Se_3 metasurfaces arising from the stochastic nature of its crystallization. We believe this change strengthens the argument and eliminates any potential ambiguity for the reader. Thank you for this suggestion, which has enhanced the accuracy of our referencing.

Corrections:--

(line 217-221, Main article): Modified

A noticeable decrease in off-resonance reflectivity can be observed in the re-amorphized state compared with the initial amorphous state in the experimental spectra of Figure 2b. In this work, this reduction is primarily attributed to incomplete re-amorphization, in which residual crystalline nuclei within the Sb_2Se_3 film enhance random scattering. This scattering effect can be equivalently described as an effective increase in the material's intrinsic absorption. Such spectral deviation is consistent with our earlier study on Sb_2Se_3 metasurfaces, where the discrepancy between simulated and experimental reflection spectra was accurately reproduced by introducing an additional extinction coefficient into the material's dielectric constant [54]. ~~Clearly, by increasing the voltage or duration of the crystallization pulse, a higher level of crystallization can be achieved. However, during the crystallization process of the one-dimensional chain-like crystalline material such as Sb_2Se_3, random lattice growth is inevitable, leading to spatially non-uniform refractive index distribution in the highly crystallized state, which affects the optical response of the metasurface [45]~~.

(Reference, Main article): Modified

[54] LI Y R, LI Y, ZENG S, et al. Lossless Phase-Change Material Enabled Wideband High-Efficiency Spatial Light Phase Modulation at Near-Infrared [J]. Laser & Photonics Reviews, 2024, 18(11).

Reviewer#2, Comment # 13:

13. On page 7 and elsewhere the authors talk about "on-chip photonic devices" – here I think they are referring to integrated photonic devices. Indeed, since their own device is a form of on-chip photonic device, the authors should be clearer as to what type of device they are referring to wen they say 'on-chip'.

Author response:

We thank the reviewer for pointing out this lack of clarity in our terminology. The reviewer is correct that the phrase "on-chip photonic devices" is broad and could encompass our own device. In the context of our original statement, we were indeed referring specifically to integrated photonic devices such as waveguides and resonators, as opposed to free-space metasurface platforms.

To avoid any ambiguity, we have revised the manuscript by replacing the generic term "on-chip" with the more precise descriptor where appropriate. This change ensures a clearer distinction for the reader. We appreciate the suggestion to improve the precision of our language.

Corrections:---

(line 77-78, Main article): Modified

In this work, we demonstrate~~, for the first time,~~ a pixelated electrically-driven Sb_2Se_3 phase-change metasurface array using ~~on-chip~~ integrated metal microthermal electrodes.

(line 91-94, Main article): Modified

Combined with neural network algorithms, we successfully demonstrate ~~on-chip~~ integrated broadband spatial spectral reconstruction over a 600 nm effective wavelength range in the short-wave infrared band, showcasing the significant potential of electrically controlled Sb_2Se_3 metasurface arrays for advanced integrated photonics.

(line 227-228, Main article): Modified

In addition, Sb_2Se_3 is considered to have the advantage of quasi-continuous phase change control and has been widely validated in ~~on-chip~~ waveguide-based photonic devices ~~[51]~~[47].

(line 276-279, Main article): Modified

Notably, prior research leveraging ~~on-chip~~ waveguide-based photonic device platforms has achieved electrically driven Sb_2Se_3 phase transitions using ultrashort electrical pulses with a width of 500 ns, validating the feasibility of sub-microsecond switching in integrated optoelectronic systems ~~[57]~~[56].

(line 319-322, Main article): Modified

From the trend of pulse voltage variation, it can be intuitively inferred that by further increasing the pulse voltage, the required pulse width for crystallization could be further reduced, potentially approaching the high-speed driving effect at the 500 ns level demonstrated by ~~on-chip~~ waveguide-based integrated devices.

Reviewer#2, Comment # 14:

14. Towards the bottom of page 8 the authors extract a crystallization time of sub-8.77 microseconds from the results shown in Fig. 3a. However, as the authors state, during the heating/switching process both volatile and non-volatile changes are occurring, and one effect can mask the other. The untangling of these two processes to extract a specific crystallization time does not seem straightforward, and the explanation given by the authors (relating to the plateau region) does not seem very convincing – certainly it would not seem reasonable to extract such a precise time of 8.77 microseconds using their arguments. The authors should strengthen their argument here if they do indeed believe the figure of 8.77 microseconds can be extracted from the result shown, or else they should be less precise and, for example, simply state that the actual crystallization time is likely to be significantly smaller than 50 microseconds.

Author response:

We thank the reviewer for this insightful and critical comment regarding our estimation of the crystallization time. We fully agree with the reviewer's assessment that extracting a precise crystallization time, such as the stated sub-8.77 μs, from the data in Fig. 3a is not sufficiently rigorous given the complexity of the physical processes involved.

As the reviewer correctly points out, the optical response during the electrical heating pulse is a convolution of several effects: the non-volatile change due to the phase transition, the volatile thermo-optic effect of the Sb_2Se_3 (Annual Review of Materials Research, 2025, 55) and surrounding materials, and potential transient thermo-mechanical deformations. Disentangling the exact temporal contribution of the phase transition itself from these concurrent volatile effects is indeed highly challenging with our current measurement methodology. Therefore, we acknowledge that the original analysis, which attributed the observed plateau region solely to the completion of crystallization, over-interpreted the data.

Consequently, we have revised the relevant section in the manuscript. The precise claim of a "sub-8.77 μs crystallization time" has been removed. The text now states more accurately that the onset of crystallization occurs rapidly and that the actual crystallization process is completed significantly faster than the 50 μs duration of the applied electrical pulse. This revision provides a more conservative and technically sound interpretation of the experimental results. We thank the reviewer for prompting this important correction, which has strengthened the accuracy of our discussion.

Corrections:--

(line 291-295, Main article): Modified

Temporal overlap between these two modulation mechanisms complicates individual response time characterization. ~~However, the stabilized photocurrent plateau observed during pulse application suggests that both thermo-optic and phase-transition processes complete within 8.77 μs during the falling edge of the photocurrent trace, indicating sub-8.77 μs crystallization of the Sb_2Se_3 metasurface.~~ Nevertheless, the rapid stabilization of the photocurrent observed during the pulse indicates that the crystallization process completes significantly faster than the 50 μs duration of the applied electrical pulse, consistent with a swift phase-transition kinetics.

Reviewer#2, Comment # 15:

15. On page 9 the authors compare the speed of their device to conventional lcd modulator devices. However, digital micromirror devices are also capable of modulation, and these have speeds into the 10s of kHz currently. Some mention of these (as competitor type devices) should be made.

Author response:

We thank the reviewer for the valuable suggestion to include digital micromirror devices (DMDs) in our discussion of high-speed spatial light modulators. We agree that DMDs are a highly relevant benchmark, currently achieving switching speeds in the tens of kHz range.

We have revised the manuscript to acknowledge DMDs as a leading high-speed commercial technology. In our current electrically driven configuration, the overall speed is indeed constrained by the thermal time constant of the integrated micro-heater, which is on a comparable order to the modulation rates of these conventional devices.

However, it is important to highlight the inherent material potential for much faster operation. Prior research utilizing direct pulsed-laser heating of Sb_2Se_3 has demonstrated complete crystallization-amorphization cycles within ~12 μs (Advanced Functional Materials, 2024, 34(14): 2310306), implying a potential modulation bandwidth in the hundreds of kHz regime for the phase-change mechanism itself. This indicates that the speed of our platform is not fundamentally limited by the Sb_2Se_3 material but by the current electrical driving architecture. Future optimization of the heater design or alternative driving schemes could therefore unlock significantly higher speeds, positioning phase-change metasurfaces as a compelling candidate for applications requiring non-volatile, high-speed, and sub-wavelength optical modulation. This more nuanced comparison and forward-looking perspective have been incorporated into the revised text.

Corrections:---

(line 307-309, Main article): Modified

Apparently, by increasing the pulse voltage to over 5 V, this study has successfully reduced the required pulse width for crystallization to 50 μs~~, indicating high-speed phase-change response superior to that of conventional liquid crystal devices~~. This switching speed is comparable to that of conventional high-speed spatial light modulators, such as liquid crystal devices and digital micromirror devices, with its overall speed primarily constrained by the thermal time constant of the integrated micro-heater. However, the inherent phase-change kinetics of Sb_2Se_3 holds the potential for significantly faster modulation. Prior studies using direct optical excitation have demonstrated full crystallization–amorphization cycles within ~12 μs, suggesting a fundamental material response capable of reaching the hundreds of kHz regime [57]. Therefore, while the present device speed is on par with established technologies, future optimization of the driving architecture could unlock substantially higher switching rates, leveraging the intrinsic fast phase-transition capability of Sb_2Se_3 for applications demanding non-volatile, high-speed optical modulation.

Reviewer#2, Comment # 16:

16. Also on page 9, the reason for limited crystallization levels is given as local hot spots restricting crystallization. Do the authors have any quantitative evidence for this, for example FE thermal simulations of the heating of their structure should be relatively straightforward to obtain to validate the hot spot mechanism.

Author response:

We thank the reviewer for the constructive suggestion regarding the need for quantitative support of the hot-spot mechanism mentioned on page 9. We fully agree that thermal uniformity of the micro-heater is a critical factor influencing the phase-change fidelity in electrically controlled metasurfaces, and it is indeed a common challenge in the field (Nature Nanotechnology, 2021, 16(6): 661-666).

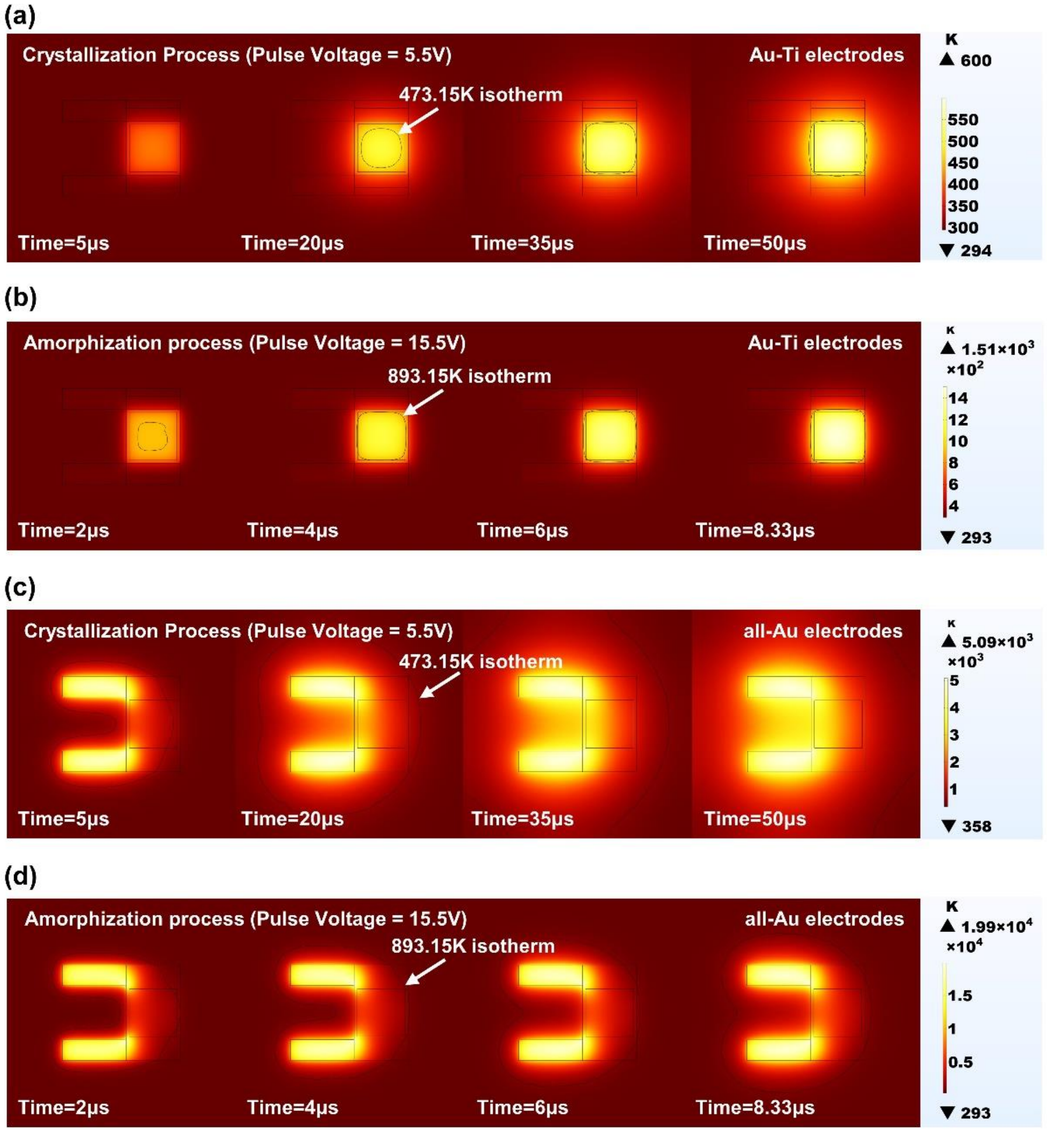

Figure R6. Comparative thermal simulations of the Ti/Au and all-Au micro-heaters. Temperature distributions during (a) crystallization and (b) amorphization for the Ti/Au design, and during (c) crystallization and (d) amorphization for the all-Au design.

In direct response to the reviewer's point, we have performed finite-element thermal simulations using COMSOL Multiphysics to analyze the electro-thermal response of our Au-Ti micro-heater structure. These new simulations have been added to the Supporting Information S1. The results quantitatively validate the presence of significant temperature non-uniformity. As shown in Figure R6, a pronounced hot spot forms in the central region of the Ti pad during both crystallization and amorphization pulses. Importantly, the simulations reveal that this non-uniformity is markedly more severe during the amorphization process. Due to the higher voltage and shorter pulse width required for melting, the temperature gradient is steeper. The simulations indicate that when the periphery of the heater reaches the minimum temperature required for amorphization (~893.15 K) (Advanced Functional Materials, 2023, 33(42): 2304601), the central region can exceed 1400 K. This substantial thermal non-uniformity likely leads to varying phase-change kinetics across the metasurface area, supporting our interpretation that it restricts the controllability and uniformity of the crystallization level.

These results underscore that overcoming intrinsic heater non-uniformity is a key engineering challenge for advancing reliable, large-scale electrically addressed phase-change metasurface arrays. We have revised the discussion on page 9 to reference this new supplementary data, strengthening our argument with the requested quantitative evidence.

Corrections:---

(line 313-317, Main article): Modified

It is believed in this study that the main reason for the limited crystallization level during rapid crystallization is the inevitable non-uniform heating of the underlying thermal electrode during the heating process. ~~Especially when dealing with large-sized metasurface pixels, crystallization pulses with narrow pulse width and high voltage tend to create significant local hotspots, which in turn restrict the overall crystallization of the metasurface pixels.~~ Especially for metasurface pixels built on large-footprint heaters, crystallization pulses with narrow width and high voltage tend to create pronounced local hot spots, which in turn limit the overall crystallization uniformity across the pixel. This thermal gradient stems directly from the extended area of the Au/Ti micro-heater and is clearly illustrated in the accompanying electro-thermal simulations (Please refer to Supporting Information S1). We regard such heater-induced temperature non-uniformity as a key factor constraining the controllability of the phase transition in the current geometry. Previous works have shown that optimizing the heater morphology can effectively mitigate this issue, pointing toward a promising direction for further improving the performance of electrically driven phase-change metasurfaces [44].

(Section S1, Supporting Information): Added

Section S1. Thermal Simulation Comparison between All-Au and Ti/Au Heater Designs

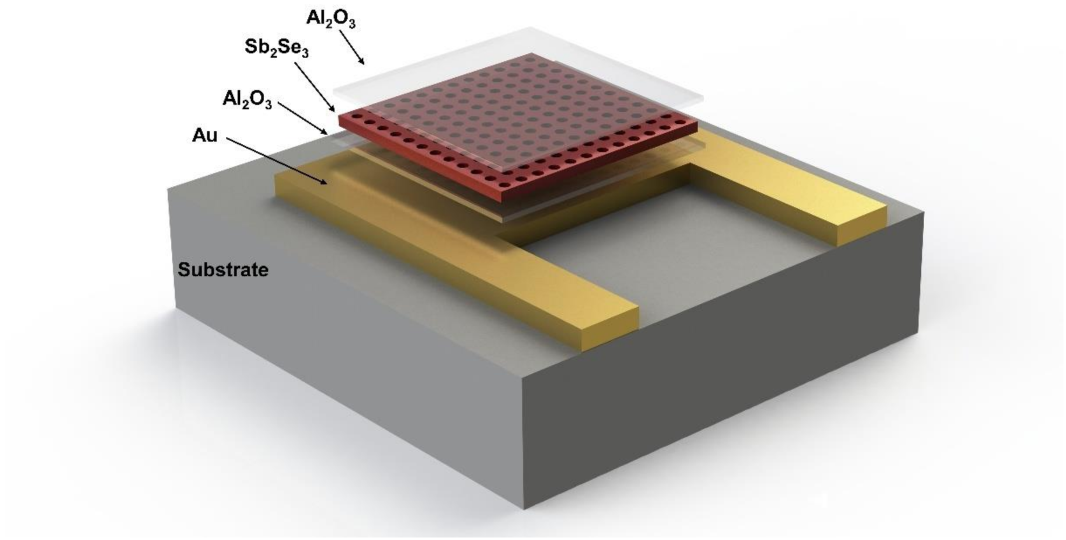

Figure S1. 3D schematic of the all-Au micro-heater integrated with the Sb_2Se_3 metasurface pixel.

In this section, we present a comparative electro-thermal analysis of the Au/Ti bilayer micro-heater employed in our work and a conventional all-Au heater. Finite-element simulations were performed using the COMSOL Multiphysics software suite to evaluate the temperature distribution within a single, representative pixel during electrical crystallization and amorphization pulses. While the simulation models an individual pixel rather than the full array, it provides sufficient insight into the fundamental heating efficiency and thermal profiles of the two designs. For a fair comparison, the pixel dimensions were kept consistent: a central rectangular heater area of 85 μm × 150 μm, with 32-μm-wide extending leads, supporting a 75 μm × 75 μm Sb_2Se_3 metasurface atop its central region (structural schematic in Figure S1).

Figure S2 displays the simulated temperature distributions under applied electrical pulses. For the Au/Ti bilayer design (Figure S2a, b), the significant resistivity contrast between the Au leads and the Ti pad effectively confines Joule heating to the central Ti region directly beneath the metasurface. The lead regions exhibit negligible temperature rise, confirming that electrical power is localized efficiently to drive the phase change. In contrast, for the all-Au heater (Figure S2c, d), the narrow lead width relative to the broad heater pad results in substantial Joule heating along the interconnect traces rather than within the target region beneath the metasurface. As a result, a significant portion of the electrical power is dissipated wastefully in the leads. Although simulations confirm that a 2.5 V, 50 μs pulse can elevate the metasurface region to the crystallization temperature (~473.15 K), the lead temperature simultaneously approaches the melting point of gold (~1064 °C), posing a serious risk of thermal damage during operation. This issue becomes even more pronounced during the amorphization process: the simulation reveals that the lead temperature already nears the gold melting threshold before the metasurface region reaches the required amorphization temperature (~893.15 K) [1]. Consequently, the all-Au design fails to provide a reliable and efficient pathway for reversible phase switching, particularly for the amorphization operation. These findings demonstrate that the all-Au heater suffers from both prohibitive power inefficiency and fundamental

limitations in achieving controlled amorphization. Therefore, the Au–Ti bilayer architecture was adopted in this work to ensure effective thermal localization, improved energy efficiency, and reliable phase-change switching.

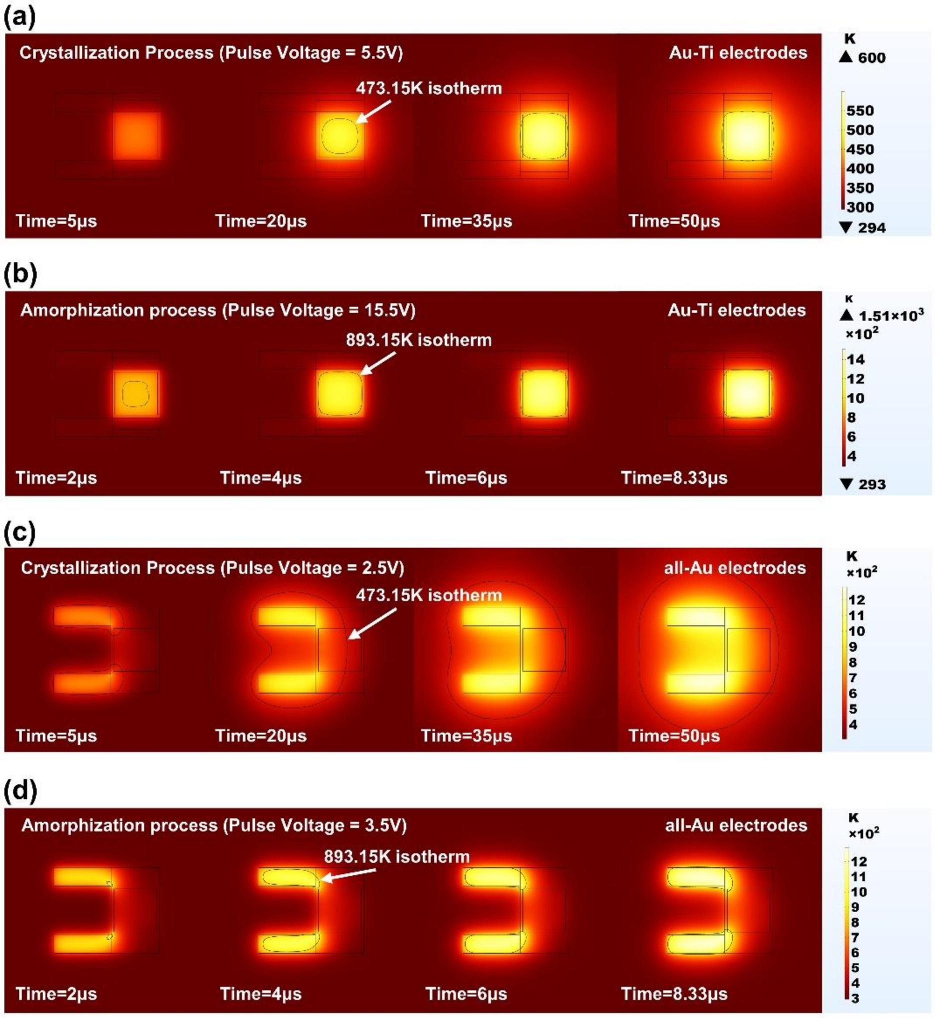

Figure S2. Comparative thermal simulations of the Ti/Au and all-Au micro-heaters. Temperature distributions during (a) crystallization and (b) amorphization for the Ti/Au design, and during (c) crystallization and (d) amorphization for the all-Au design.

It is also important to note a key limitation of our current Au/Ti design: significant thermal non-uniformity, or a central "hot spot," is observed within the Ti pad (Figure S2a, b). This inhomogeneity is particularly pronounced during the high-power, short-duration amorphization pulse. For instance, under a 15.5 V, 8.33 μs amorphization pulse, while the entire Ti pad exceeds the required amorphization temperature of Sb_2Se_3 (~893.15 K), the central region reaches nearly 1500 K. This thermal gradient is a critical factor limiting the uniformity and controllability of the phase transition in the Sb_2Se_3 metasurface. Prior studies have proposed geometric optimization of heater shapes to improve thermal uniformity [2], which represents a promising direction for future refinement of our platform.

Section on phase-change metasurface array for spatial spectral reconstruction
Reviewer#2, Comment # 17:

17. In Fig 4c, the authors should make clear if the transmission matrix shown is experimental or simulation.

Author response:

We thank the reviewer for this query regarding the data presented in Figure 4c. The transmission matrix shown in the figure is constructed from experimentally measured reflection spectra of the 6×6 metasurface array. We apologize for any ambiguity in the original caption. In the revised manuscript, we have explicitly stated in the figure caption that the spectra are experimental data to ensure clarity for the reader.

Corrections:---

(Figure 4, Main article): Modified

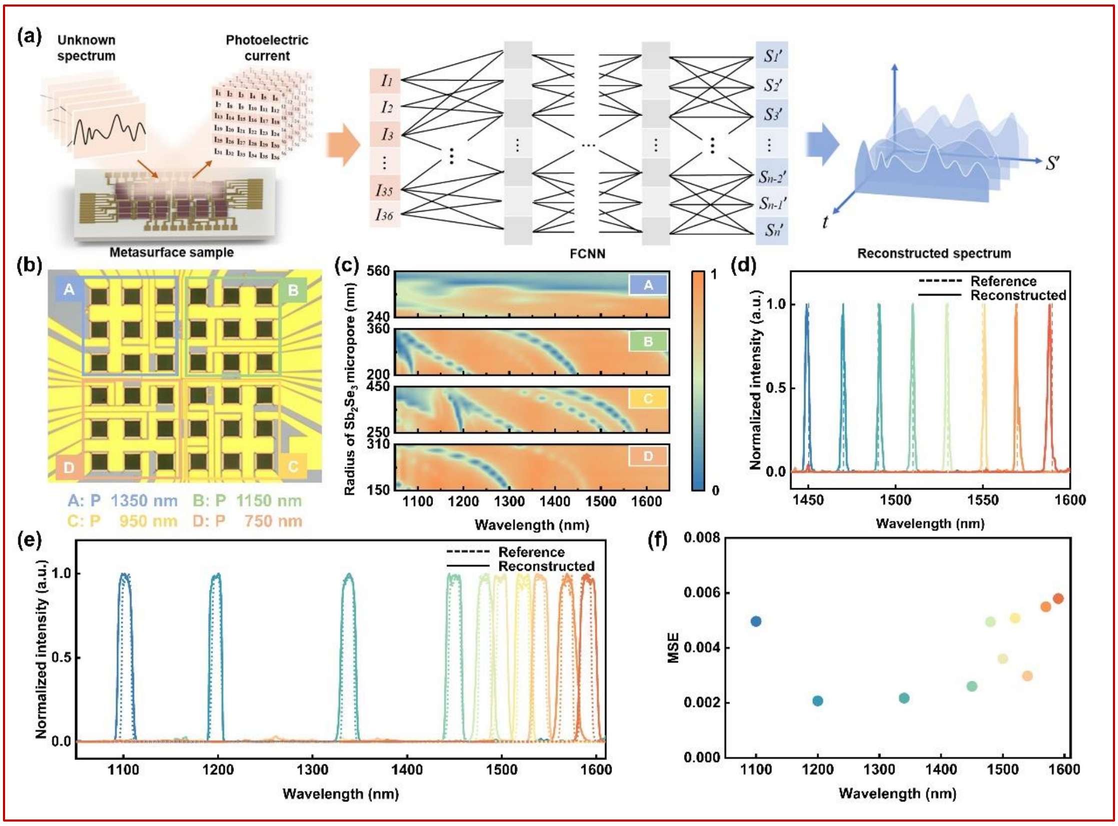

Figure 4. Experimental validation of spatial spectral reconstruction using the 6×6 Sb_2Se_3 metasurface array. a, Schematic illustration of the spatial spectral reconstruction process based on the metasurface array. b, Microscopic image of the fabricated metasurface array for spatial spectral reconstruction. c, Experimentally measured ~~S~~spectral transmission matrix constructed from the reflection spectra of the metasurface array. d, Performance validation of single-wavelength spectral reconstruction within the wavelength range of 1450–1590 nm. e, Reconstruction of the transmission spectra of narrowband filters within the wavelength range of 1100–1590 nm. f, Mean squared error (MSE) of the reconstructed transmission spectra of narrowband filters with different central wavelengths.

Reviewer#2, Comment # 18:

18. Why is the duty cycle limited to 50%? Is it because the electrode leads are damaged for higher duty cycles, and if this is the case, would the duty cycle not depend on the voltage applied (and current flowing)?

Author response:

We thank the reviewer for the insightful question regarding the duty cycle limitation. Indeed, the fill factor in our current implementation is a significant constraint, which we directly attribute to the inherent limitations of the planar wiring scheme used for individually addressing each pixel.

The primary reason for this limited fill factor is the need to allocate sufficient space between pixels for the metal leads that connect each micro-heater to the periphery. As the array size increases, the number of leads routed through the inter-pixel gaps grows, requiring more space. Crucially, the width of these leads cannot be reduced arbitrarily without consequence. Excessively narrow leads would exhibit high electrical resistance, leading to increased parasitic power dissipation and potential reliability issues due to electromigration or overheating. This fundamental trade-off forces an expansion of the inter-pixel spacing at the expense of the fill factor. Therefore, in this architecture, the fill factor is primarily dictated by the routing density required by the wiring topology, not directly by the applied voltage or current.

To directly address this fundamental limitation, we propose a potential solution in the revised manuscript by introducing and analyzing an alternative vertically stacked, row-column addressing architecture. Our numerical simulations demonstrate the feasibility of this approach for independent pixel control. Notably, even without dedicated optimization of the pixel layout or interconnect width, this architecture readily achieves a fill factor of approximately 50%. It is clear that through further optimization of the pixel pitch and the routing of the row/column lines, the fill factor can be substantially increased without impeding the further scaling of the array size. This proposed architectural shift offers a promising pathway to overcome the fill-factor constraints inherent in the current planar wiring scheme, and its implications have been added to the revised manuscript.

Corrections:---

(line 163-166, Main article): Modified

To address this, this work effectively reduces the overall number of required leads by sharing the ground line among pixels and optimizes the lead layout, demonstrating a two-dimensional random-access phase-change metasurface array with a pixel effective size of 75 μm and a pixel pitch of 150 μm, achieving an effective fill factor of nearly 50%.

We emphasize that the choice of a 6×6 array represents a deliberate practical balance for this proof-of-concept study. This scale provides a transmission matrix with sufficient dimensionality to validate the principle of broadband spatial-spectral reconstruction, while remaining manageable for the fabrication and characterization processes. It does not reflect a fundamental physical or architectural limit of the phase-change material or metasurface paradigm itself. The primary limitation on scalability in the current implementation stems from the planar wiring scheme used for individual pixel addressing. As noted, this design entails an inherent compromise between array size and fill factor: scaling the pixel count necessitates more interconnecting leads, which in turn

reduces the usable active area. In contrast, the row-column addressed electrode architecture discussed in the Supporting Information S2 is designed to be compatible with both a high fill factor and straightforward array scaling. By vertically stacking the addressing lines and sharing them across rows and columns, this approach presents a promising and practical pathway for future research toward large-scale, densely integrated phase-change metasurface arrays.

Reviewer#2, Comment # 19:

19. On page 12 the authors mention ‘second order phase change control’ – what do they mean here, is it just partial/weak crystallization again, or something different? Please clarify.

Author response:

We thank the reviewer for requesting clarification on the term "second order phase change control" used on page 12. We acknowledge that this phrasing was ambiguous and could be misleading.

In this context, the term denotes the use of two distinct optical states of the metasurface, the amorphous state and an electrically switched crystalline state, to construct the spectral transmission matrix. To eliminate any confusion, we have revised the manuscript. The term "second order" has been replaced with an explicit explanation that the two levels correspond to the amorphous and a partially crystallized state.

Corrections:---

(line 408-416, Main article): Modified

~~It is worth noting that the significant optical contrast between the amorphous and crystalline states of Sb_2Se_3 enables a substantial difference in the spectral responses of the metasurfaces. This characteristic can be leveraged to enrich the spectral transmission matrix by incorporating the active spectral response of the phase-change metasurface, thereby further enhancing the non-correlation of the spectral transmission matrix. In other words, by integrating phase-change modulation of the metasurface pixels, the current work can reduce the required number of metasurface pixels for spectral reconstruction while maintaining broadband spectral sampling capabilities. Here, taking second-order phase change control as an example, the current work further reduces the required number of metasurface pixels for high-precision broadband spectral reconstruction to 18.~~ Following the validation of the 36-channel transmission matrix constructed from the static metasurface array, which demonstrates effective spectral reconstruction across the 1100–1590 nm band, we next use its performance as a baseline for comparison. We then evaluate the reconstruction capability of a second 36-channel matrix built from only 18 physical pixels by leveraging both their amorphous and crystalline states. This direct comparison within the identical spectral window allows us to assess the effectiveness of using phase-change tuning to expand the dimensionality and sampling capacity of a compact metasurface array. Figure 5a shows the selected 18 metasurface pixels, and a ~~36-order~~ 36-channel spectral transmission matrix is constructed using their reflection spectra at both the amorphous and crystalline states.

Conclusions

Reviewer#2, Comment # 20:

20. The authors claim their work demonstrates for the first time an electrically controlled two-dimensional phase-change metasurface array. However, other researchers are also currently developing electrically controlled 2d metasurface arrays - for example Hu et al at MIT recently (September 2025) demonstrated a pixelated Sb2S3 array device for mid-infrared spatial light modulation at the ECPOS 2025 conference in Marseille. So, perhaps the claim of being 'first' is open to discussion and should be downplayed?

Author response:

We thank the reviewer for bringing this recent and relevant work to our attention. We are aware of the excellent progress in the field, including the work presented by Hu et al. at ECPOS 2025. We agree that in such a rapidly advancing research area, claims of being the "first" can be nuanced and may benefit from a more focused description of the specific advances demonstrated.

In the revised manuscript, we have therefore moderated the language regarding priority. The narrative has been adjusted to emphasize the key specific achievements of our work - namely, the experimental demonstration of a fully addressable, two-dimensional 6×6 active metasurface array based on low-loss Sb_2Se_3 for near-infrared modulation and its functional application in computational spectral reconstruction, rather than making a broad claim of being the first. We believe this presents our contribution more accurately and appropriately within the context of concurrent developments.

We appreciate the reviewer's suggestion, which has helped us refine the framing of our work to be both precise and collegial.

Corrections:---

(line 66-75, Introduction): Modified

To overcome this, indirect electrical control using integrated micro-heaters~~ emerged, successfully demonstrating phase switching in materials like GST and GSST with larger pixel sizes reaching 0.4×0.4 mm^2 [49]. ~~was developed. This approach has been successfully applied to various phase-change materials, including GST [41-42] and GSST [43-44], and validated for both transmissive [43] and reflective spatial light modulation [41-42,44]. Very recently, this indirect electrical control approach has been further extended to two-dimensional, addressable phase-change metasurface arrays based on GSST, providing a promising new route for pixelated spatial light modulation [45]. Despite these advances in electrical control methodologies, a critical gap remains.~~: while low-loss PCMs such as Sb_2Se_3 and Sb_2S_3 are highly promising for efficient optical modulation [40-42], their effective integration into electrically driven phase-change metasurfaces has not yet been demonstrated.~~ Low-loss PCMs such as Sb_2S_3 and Sb_2Se_3 have attracted significant attention due to their uniquely low optical loss in both amorphous and crystalline states, along with the inherent advantage of their binary composition, which suppresses phase separation. Although early studies indicated that their crystallization could suffer from spatial randomness, posing a challenge for large-area, uniform modulation, recent work on spatially confined, nucleation-guided crystallization has demonstrated more uniform phase switching in Sb_2S_3, renewing interest in these materials [46]. Nevertheless, their integration into electrically driven phase-change metasurfaces has thus far been relatively

unexplored, and a practical, scalable platform for dynamically controlling Sb_2S_3 or Sb_2Se_3 metasurfaces via integrated electronics has not yet been demonstrated. Although Sb_2Se_3 phase switching via PIN heaters in integrated photonics and recently in metasurfaces as a tunable dielectric environment has been reported [51-52][47-48], these approaches introduce high-resistance microheaters, micrometer-scale pixel constraints, and necessitate complex optical designs to accommodate the high-index Si substrate, significantly limiting Sb_2Se_3/Sb_2S_3 metasurface scope and performance.

(line 77-78, Main article): Modified

In this work, we demonstrate, for the first time, a pixelated electrically-driven Sb_2Se_3 phase-change metasurface array using on-chip integrated metal microthermal electrodes.

(line 444-474, Main article): Modified

In summary, the current work, leveraging the low-loss phase-change material Sb_2Se_3, experimentally demonstrates for the first time an electrically controlled two-dimensional phase-change metasurface array. To fully exploit the advantages of Sb_2Se_3, including its low loss and high optical contrast in the short-wave infrared band, we propose an electrically controlled metasurface pixel design architecture that combines metallic microheater electrodes with hybrid phase-change metasurfaces. This design achieves complete decoupling between the electrically induced phase change and the optical response of the metasurface. By selectively confining the electromagnetic fields of the metasurface resonance modes within the phase-change microstructures or the surrounding dielectric environment, the current work experimentally demonstrates the excitation of both under-coupled and over-coupled resonance modes. In the short-wave infrared band, we achieve amplitude-dominant modulation with an efficiency of 60%80% and phase-dominant modulation covering nearly 6 radians, thereby verifying the multifunctional characteristics of the electrically controlled metasurface pixels. Moreover, benefiting from the low resistance and high thermal conductivity of the metallic microheater electrodes, the current work showcases low-threshold-voltage (both crystallization and amorphization pulse voltages are less than 10 V) and high-speed (crystallization pulse time of 50 ms μs and amorphization pulse time of 8.33 μs) electrically induced phase change control on 75-μm Sb_2Se_3 metasurface pixels. This indicates the significant potential of the current metasurface pixel design for high-speed spatial light field modulation and other applications. Building on this, the current work further demonstrates a two-dimensional electrically controlled phase-change metasurface array, with an array size of 6×6 and an effective fill factor close to 50%. Using integrated spatial spectral reconstruction as an example, we highlight the multifaceted application value of the two-dimensional electrically controlled phase-change metasurface array. By leveraging the densely packed multi-resonance modes excited by the Sb_2Se_3 hybrid phase-change metasurface and combining phase-change spectral modulation, the current work achieves broadband, high-precision spatial spectral reconstruction in the short-wave infrared band with an effective bandwidth coverage of nearly 500 nm (1100-1590 nm) using only 18 metasurface pixels. As the first demonstration of a two-dimensional phase-change spatial light field modulation array, Overall, our work holds significant importance for promoting the development of integrated phase-change spatial light field modulation technologies and devices, as well as for expanding the applications of phase-change spatial light field modulation technologies in sensing, imaging, computing, and other fields.
